# Virome-wide ubiquitin ligase discovery reveals diverse mechanisms of immune evasion

**DOI:** 10.1126/science.aec6299

**Published:** 2026-08-20

**Authors:** Caleb R. Glassman, Kheewoong Baek, Gaopeng Hou, Qiru Zeng, Christopher Nardone, Kate B. Juergens, Eric Fujimura, Colin N. O’Leary, Mamie Z. Li, Joao A. Paulo, Eric S. Fischer, Siyuan Ding, J. Wade Harper, Stephen J. Elledge

**Affiliations:** 1 Department of Genetics, Harvard Medical School, Boston, MA 02115, USA.; 2 Division of Genetics, Brigham and Women's Hospital, Boston, MA 02115, USA.; 3 Howard Hughes Medical Institute, Boston, MA 02115, USA.; 4 Department of Cancer Biology, Dana–Farber Cancer Institute, Boston, MA 02215, USA.; 5 Department of Biological Chemistry and Molecular Pharmacology, Harvard Medical School, Boston, MA 02115, USA.; 6 Department of Molecular Microbiology, Washington University School of Medicine in St. Louis, St. Louis, MO, USA.; 7 Program in Biological and Biomedical Sciences, Harvard Medical School, Boston, MA 02115, USA.; 8 Program in Virology, Harvard Medical School, Harvard University, Boston, MA 02115, USA.; 9 Program in Chemistry and Chemical Biology, Harvard University, Cambridge, MA 02138, USA.; 10 Department of Cell Biology, Harvard Medical School, Harvard University, Boston, MA 02115, USA.

## Abstract

Viruses are intracellular parasites that reprogram the host proteome to promote replication and evade immune recognition. We applied a virome-wide library of ~10,000 open reading frames to discover viral ubiquitin ligases, mapping their mechanisms of degradation and host substrates using targeted CRISPR screens and proteomics. These viral effectors could be classified as canonical ligases that mimic host E3s, hijackers that redirect host E3s, and non-canonical ligases that rewire Cullin-RING ligase machinery. These diverse strategies of virus-mediated degradation converged on immune-related substrates, including JAK1 and CUL1^β–TrCP^, underscoring immune evasion as a major driver of viral ubiquitin ligase evolution. Our findings elucidate viral strategies for exploiting the ubiquitin–proteasome system with potential for therapeutic targeting.

Viruses are obligate intracellular pathogens that reprogram host cells to facilitate replication and evade immune detection. In response, host cells rapidly activate antiviral signaling pathways including translation inhibition (1), interferon induction (2), and inflammatory cell death (3). To establish a productive infection, viruses must quickly rewire host cellular physiology to suppress the immune response and redirect cellular resources toward viral replication.

Post-translational modifications (PTMs) enable viruses to alter protein function rapidly without the delays associated with transcription, translation, and basal protein turnover. The ubiquitin-proteasome system (UPS) is a modular cascade of E1, E2, and E3 enzymes that tag substrates with ubiquitin, thereby altering function, and, in some cases, leading to rapid protein degradation (4). In this pathway, E3 ubiquitin ligases dictate substrate specificity by bridging substrate to ubiquitin-charged E2. Viruses can co-opt this system either by encoding their own E3s or by binding and redirecting host E3s, allowing the virus to induce rapid changes in protein expression (5–7). We refer to the family of viral proteins that induce degradation of cellular substrates as “degradins”. Viruses exploit the UPS to alter diverse aspects of cell physiology quickly, including preventing apoptosis (8), protecting against viral genome modification (9, 10), and evading adaptive immune detection (11–13).

Despite the importance of this mode of regulation, viral ubiquitin ligases have either been identified using proteomics, as in the case of HIV-Vif (14), or by sequence homology with host ligases, such as Vaccinia virus broad-complex, tramtrack, and bric-a-brac (BTB) domain-containing proteins (15, 16). Nonetheless, many viral ubiquitin ligases lack homology to human E3s and cannot be predicted from sequence alone. Thus, we employed a genetic screening approach to identify viral regulators of ubiquitination systematically, leveraging a recently developed viral open reading frame (vORF) library, which includes genes from diverse human and animal viruses (17). For this screen, each viral protein was fused to a green fluorescent protein (GFP)-specific nanobody (αGFPnb), allowing us to monitor GFP fluorescence as a proxy for degradation activity by fluorescence-activated cell sorting (FACS). We next used CRISPR screens to identify required UPS components and proteomics to determine substrates for individual viral genes. This approach enabled us to mechanistically characterize viral ubiquitin ligases with distinct mechanisms and substrates. Thus, our approach provides a rapid and unbiased method for uncovering and characterizing viral degradins that exploit the UPS, shedding light on both known and emerging viral immune evasion strategies.

## A pooled genetic screen reveals viral regulators of protein stability

Ubiquitin ligases can catalyze the formation of K48-linked ubiquitin chains on substrates, leading to their degradation (4). Previously, nanobody fusions have been used to control post-translational modifications, including phosphorylation (18), glycosylation (19), and ubiquitination (20). Thus, we tested whether fusing a viral ubiquitin ligase to an αGFPnb (21) would result in GFP degradation using a modified Global Protein Stability (GPS) assay in which GFP fluorescence is measured relative to a DsRed control (22). To establish our system, we used Rotavirus A NSP1, which degrades IRF3 in a viral strain-dependent manner (23). While both doxycycline-inducible 3xHA-NSP1 and αGFPnb-NSP1 robustly degraded GFP-IRF3 (Fig. 1A-B), only αGFPnb-NSP1 was able to degrade GFP alone (Fig. 1C-D). A similar approach has been used to identify human proteins capable of inducing degradation (24), highlighting the generality of this strategy.

To identify viral proteins that function as ubiquitin ligases systematically, we expressed a library of viral open reading frames fused to the αGFPnb and performed a FACS-based screen for loss of GFP fluorescence relative to a DsRed control (Fig. 1E). Analysis of this screen revealed known viral regulators of the UPS including HIV-Vpr (25), Rotavirus A NSP1 (23, 26), Fowl adenovirus A ORF8 (27), human adenovirus E4orf6 (28, 29), and African swine fever virus E2 (30) along with many viral proteins without described roles in protein degradation (Fig. 1F, Data S1). Although our screen captured many known viral ubiquitin ligases, it likely missed other bona fide ubiquitin ligases owing to incompatibility with nanobody fusion or failure to degrade GFP as a neo-substrate. Because the nanobody-based screen is intentionally agnostic about both the substrates and mechanism of degradation, we performed follow-up CRISPR screens to identify host cell dependencies and proteomics to identify substrates (Fig. 1E).

## Rotavirus A NSP1 assembles a non-canonical CUL3^ELOB/C^ complex to suppress antiviral RNA sensing

Rotavirus A is a double-stranded RNA virus that can cause severe diarrhea in young children. Vaccination has proved highly effective at limiting hospitalization and deaths associated with Rotavirus infection; however, it remains a public health concern in developing countries (31, 32). The NSP1 gene from Rotavirus A is an interferon antagonist that interacts with multiple components of the host Cullin-RING ligase (CRL) system in a strain-dependent manner (26, 33). However, the mechanism by which NSP1 mediates interferon suppression remains unclear (34). We sought to gain further mechanistic insight into this process while validating our workflow for viral ubiquitin ligase characterization. αGFPnb-NSP1 led to degradation of GFP that was dependent on CRLs (blocked by the NEDD8-activating enzyme inhibitor MLN4924), ubiquitination (blocked by the ubiquitin-activating enzyme E1 inhibitor TAK243), and the proteasome (blocked by the proteasome inhibitor Bortezomib) (Fig. 2A). Given that many E3s are unstable owing to their proximity to the ubiquitination reaction, we performed a CRISPR screen for NSP1-GFP stability targeting ~1,500 ubiquitin-related genes. This screen revealed a critical role for the Cullin 3 complex (CUL3/NEDD8/RBX1) and the proteasome (Fig. 2B, Data S2A-B), consistent with previous reports (33).

Immunoprecipitation-mass spectrometry (IP-MS) of HA-tagged NSP1 confirmed a physical interaction with CUL3 as well as the known substrates IRF3 and SAMD9 (Fig. 2C, Data S3A and S3G) (33, 35). Typically, host ubiquitin ligases interact with CUL3 via a BTB domain (36); however, AlphaFold predicts that NSP1 lacks a BTB domain (37), and we did not observe BTB domain-containing proteins in the IP-MS. Instead, NSP1 IP pulled down Elongin C (ELOC), which is structurally similar to BTB domains and the CUL1 adaptor SKP1 (38, 39) but typically acts as an adaptor for CUL2 and CUL5. Given these structural similarities, we hypothesized that ELOC might function in place of a BTB domain to facilitate CUL3 binding. ELOC (*TCEB1*) and ELOB (*TCEB2*) also scored in the CRISPR screen for host cell dependencies, suggesting this interaction may be important for NSP1 function (Fig. 2B). Consistent with this, NSP1-mediated degradation required both CUL3 and ELOC (fig. S1A), and Cas9-mediated mutation of ELOC resulted in attenuated CUL3 binding (fig. S1B). Furthermore, purified NSP1-CUL3^ELOB/C^ showed robust ubiquitination of IRF3 in vitro (fig. S1C). To characterize the complex structurally, we screened NSP1 genes from distinct Rotavirus A strains for expression and activity, which enabled us to identify a variant with improved expression in mammalian and insect cells (ID: B3SRV2.0.1). We confirmed that NSP1 genes from both strains formed a CUL3^ELOB/C^ complex by performing reciprocal IPs (Fig. 2D). As expected, NSP1 pulled down both FLAG-tagged CUL3 and ALFA-tagged ELOC. Furthermore, we observed an interaction between ALFA-ELOC and FLAG-CUL3 that depended on NSP1 expression, consistent with ternary complex formation.

To elucidate the mechanism of NSP1 complex assembly, we determined a 3.3 Å structure of CUL3-RBX1-ELOB-ELOC-NSP1 (ID: B3SRV2.0.1) by cryo-electron microscopy (cryo-EM) (Fig. 2E, Table S1, fig. S1D). The overall geometry of the NSP1-CUL3^ELOB/C^ complex is similar to host ubiquitin ligases bound to CUL2^ELOB/C^ and CUL5^ELOB/C^, with the aromatic-rich N-terminus of the Cullin interacting with the base of ELOC (40–44). Typically, CUL2/5 ligases use an ELOB/C binding motif (BC-box) consisting of small hydrophobic residues arranged on a helix to bind ELOC, however, NSP1 uses a helix anchored by a central phenylalanine residue (Phe^210^) to bind to the hydrophobic binding pocket of ELOC. The BC-box motif is usually followed by a proline-rich Cullin box that facilitates a small interaction between the Cullin and E3. In contrast, NSP1 uses a second helix that makes extensive interactions with ELOC and CUL3 to stabilize ternary complex formation. Indeed, interface residues are conserved across NSP1 genes from diverse Rotavirus A strains and AlphaFold 3 predictions suggest a similar binding mode (fig. S2A-B). Previously, ZSWIM8 has been reported as a CUL3^ELOB/C^ adaptor with roles in target-directed miRNA degradation (45, 46). Unlike NSP1, ZSWIM8 contains a canonical BC-box, suggesting that its mode of assembly may be more similar to traditional CUL2/5 ligases. Indeed, a recent structure of the CUL3-ELOB/C-ZSWIM8 complex reveals ZSWIM8 uses a proline-rich motif to bind to CUL3 (47).

Despite conservation of CUL3^ELOB/C^ binding regions, Rotavirus A strains differ substantially at their C-termini, with some strains having the ELLISD motif associated with IRF3 binding (48) and others having a β-TrCP degron, DSGXXD, to recruit and degrade β-TrCP thereby inhibiting NFκB signaling (33, 49–51). Mutation of the β-TrCP degron or CUL3^ELOB/C^ binding site attenuated β-TrCP degradation (Fig. 2F) while uncoupling substrate and Cullin binding (fig. S2C). The analogous mutations in the IRF3-binding NSP1 variant showed a conserved role for these residues in CUL3^ELOB/C^ binding and substrate degradation (Fig. 2G, fig. S2D). Consistent with the shared role of β-TrCP and IRF3 in IFN induction, NSP1 variants from both strains suppressed IFNβ induction following Sendai virus infection in a manner dependent on binding to their respective substrates, β-TrCP and IRF3, as well as CUL3 and ELOC (fig. S2E-F).

To assess the physiological importance of CUL3^ELOB/C^ recruitment during live virus infection, we generated recombinant viruses using the well-established Rotavirus SA11 reverse genetics system (52). SA11 harboring NSP1 mutations in the CUL3^ELOB/C^ binding interface failed to degrade NSP1 substrates IRF3 and SAMD9 in MA104 cells (fig. S2G). Multi-step growth in interferon-competent HT-29 cells showed that mutation of the ELOC or CUL3 binding site attenuated viral expansion relative to wild-type control; however, the reduction was less than that of NSP1 deletion, suggesting additional roles for NSP1 beyond substrate degradation (Fig. 2H, fig. S2H).

As a double-stranded RNA virus, Rotavirus can be recognized by cytoplasmic RNA sensors RIG-I and MDA5, which activate MAVS and promote nuclear localization of IRF3 and NFκB, culminating in expression of type I interferon genes. Thus, NSP1 strains use conserved interactions with CUL3^ELOB/C^ to inhibit RNA sensing and prevent the induction of type I interferon, but do so by targeting distinct substrates, highlighting the importance of this immune evasion axis for Rotavirus A virulence.

## Teviot virus matrix protein co-opts CUL3^BTBD1^ to degrade JAK1 and inhibit IFNβ signaling

We next considered the matrix protein (M) from Teviot virus (TevPV). Teviot virus is a bat-borne *Paramyxovirus* with a negative-strand RNA genome (53). Although matrix proteins typically serve a structural role in virion assembly, they can also have regulatory functions (54). TevPV-M-mediated degradation required CRL activity and a ubiquitin-focused CRISPR knockout screen identified CUL3 and BTBD1 as required host factors (Fig. 3A-B, Data S2C-D). IP-MS identified components of the β-catenin destruction complex (CTNNB1/APC) as well as the CUL3 adaptor BTBD1 and components of the TRiC chaperonin complex (Fig. 3C). Cas9-mediated mutation of CUL3 or BTBD1 but not the related adaptor BTBD2 attenuated M-mediated degradation (Fig. 3D). AlphaFold 3 predicted TevPV-M binds BTBD1 adjacent to but outside of the canonical substrate binding motif (fig. S3A) (55). Mutating residues in TevPV-M at the predicted BTBD1 interface prevented binding to CUL3 and ALFA-tagged BTBD1 (fig. S3B). Despite binding to multiple components of the β-catenin destruction complex, expression of TevPV-M did not result in a change in β-catenin stability (fig. S3C).

To identify potential substrates, we performed whole-cell proteomics. Janus kinase 1 (JAK1) levels were reduced upon TevPV-M expression (Fig. 3E, Data S4A). JAK1 is a tyrosine kinase that mediates cytokine signaling for multiple cytokines including Type I interferons, such as IFNβ. Wild-type TevPV-M but not a BTBD1 binding mutant resulted in degradation of JAK1, consistent with JAK1 being a substrate (Fig. 3F). JAK1 was not detected in the IP-MS with TevPV-M, however, this may reflect low affinity between substrate and viral protein. To capture the interaction, we transfected cells expressing TevPV-M under doxycycline inducible control with ALFA-JAK1 and performed affinity purification. JAK1 pulled down TevPV-M independently of TevPV-M’s interactions with BTBD1, indicating a physical association between the viral effector and the host substrate (fig. S3D). In IFNβ signaling, cytokine binding dimerizes receptor-associated TYK2 and JAK1 kinases resulting in transphosphorylation (56). TevPV-M reduced the total levels of JAK1 but not TYK2 while attenuating phosphorylation of both kinases as well as transcription factors STAT1 and STAT2 in a manner that depended on interactions with BTBD1 (Fig. 3G).

Ubiquitin ligases and adaptors are often unstable owing to their interactions with the UPS. Because matrix protein is a structural component of the virion, viruses may face a trade-off between robust protein expression and effective substrate degradation. Indeed, TevPV-M levels were enhanced by treatment with CRL inhibitor MLN4924 or mutation of residues at the BTBD1 binding site, both of which prevented degradation of JAK1, suggesting viruses may need to balance virion assembly with immune evasion (Fig. 3H, fig. S3E).

## Adana virus NSs recruits CUL1^β-TrCP^ to degrade JAK1 and inhibit IFNβ signaling

The NSs gene from Adana virus, an insect-borne *Phlebovirus* (57), scored as a putative viral degradin. NSs-mediated degradation was dependent on CRLs (Fig. 4A) and a CRISPR screen identified CUL1, SKP1, and β-TrCP2 (*FBXW11*) as required host factors (Fig. 4B, Data S2E-F). Consistent with CUL1 involvement, NSs interacted with multiple components of the CUL1^β-TrCP^ complex (β-TrCP1, β-TrCP2, CUL1, and SKP1) by IP-MS (Fig. 4C, Data S3C and S3G). Manual sequence inspection revealed a canonical β-TrCP degron (DSGIST) at the NSs C-terminus and Cas9-mediated mutation of CUL1 and β-TrCP2 but not β-TrCP1 resulted in reduced degradation (Fig. 4D). Also, among the bound proteins was JAK1, which we considered as a potential substrate. Indeed, NSs reduced JAK1 expression in a manner dependent on the C-terminal β-TrCP degron in NSs (Fig. 4E). Notably, NSs lacks acceptor lysine residues upstream of the degron typically required for β-TrCP-mediated ubiquitination (58), which may explain its primary role as an adaptor rather than as a substrate. Truncation experiments suggested the JAK1 pseudokinase domain was critical for its degradation (fig. S4A) and AlphaFold 3 predictions indicated NSs engages the N-lobe of the JAK1 pseudokinase domain at the JAK1 dimerization interface (Fig. 4F) (59). Mutating the β-TrCP degron abolished β-TrCP binding but not the JAK1 interaction, while mutation of the predicted JAK1 binding site reduced JAK1 binding without attenuating the β-TrCP interaction (Fig. 4G). Similarly, mutations to the JAK1 pseudokinase domain at the predicted interface resulted in attenuated degradation by NSs (fig. S4B).

To test the functional importance of JAK1 degradation, we assessed signaling pathway activation following IFNβ stimulation. Wild-type NSs led to a modest reduction in JAK1 levels but to strong suppression of JAK1, TYK2, and STAT1 phosphorylation, which was dependent on β-TrCP and JAK1 binding (Fig. 4H). Together, these results indicate Adana virus NSs co-opts CUL1^β-TrCP^ to degrade JAK1 and suppress IFNβ signaling (fig. S4C).

Previously, the structurally related NSs gene from Rift valley fever virus (RVFV) has been reported to have multiple roles in protein degradation (60–62). Primary sequence alignment and AlphaFold 3 predictions of NSs genes from diverse *Phlebovirus* species suggested that JAK1 binding was restricted to a closely related clade of viruses despite conservation of the overall NSs fold (fig. S4D-E). Although RVFV NSs has been identified previously to interact with β-TrCP (61), the canonical β-TrCP degron we identified in Adana virus and related species is not conserved in RVFV. Instead RVFV uses a phosphomimic degron distinct from that of Adana virus, highlighting the mechanistic diversity of co-option in this viral family. By degrading JAK1, both Adana virus and Teviot virus attenuate IFNβ signaling but do so through distinct Cullin complexes, highlighting the critical role for JAK1 in viral restriction.

## Razdan virus NSs recruits CUL3 to degrade CUL1 and inhibit NFκB signaling

The NSs genes from Razdan (63) and Bhanja (64–66) virus, two closely related tick-borne *Bunyaviruses*, scored as putative degradins and we followed up on Razdan NSs for initial validation and characterization. Razdan NSs induced CRL-dependent degradation that required CUL3 (Fig. 5A-B and S5A, Data S2G-H). Consistent with this, NSs bound strongly to CUL3, but no BTB-domain adaptor proteins were found by IP-MS, suggesting a possible direct interaction (fig. S5B, Data S3D and S3G). Indeed, AlphaFold 3 predicted the Razdan NSs C-terminal region directly engages CUL3 at the typical BTB domain binding site (fig. S5C). Whole-cell proteomics revealed a loss of CUL1 and stabilization of multiple CUL1 substrates (CTNNB1, ORC1, EID1, IREB2, MORF4L1/2) and F-box proteins (CCNF, FBXO28), suggesting that CUL1 is degraded by NSs (Fig. 5C, Data S4B). Indeed, both Razdan and Bhanja NSs robustly degraded CUL1 in a CRL-dependent manner (Fig. 5D). To test the impact of NSs expression on CUL1 activity, we used a GPS reporter consisting of a peptide from IFNA8 previously identified in a genome-wide screen for peptide degrons that is regulated by CUL1^FBXO21^ (67). Expression of Razdan or Bhanja NSs stabilized this reporter demonstrating functional inhibition by CUL1 degradation (Fig. 5E). Degradation of CUL1 was dependent on Razdan NSs residues at the predicted CUL3 binding interface, consistent with NSs functioning as a non-canonical CUL3 ubiquitin ligase (fig. S5D). Reciprocal IP between ALFA-tagged CUL1 and HA-tagged NSs showed binding independent of interactions with CUL3 (fig. S5E) and a cell-based ubiquitination assay that revealed NSs ubiquitinated CUL1 in a CUL3-dependent manner (fig. S5F).

Targeting β-TrCP is a common viral immune evasion strategy because activation of NFκB signaling requires CUL1^β-TrCP^-mediated degradation of phosphorylated IκBα, releasing NFκB for nuclear translocation (33, 49–51, 68). In TNFα treated cells, expression of Razdan or Bhanja virus NSs attenuated IκBα degradation and resulted in a size shift by SDS-PAGE, which corresponded to an increase in phosphorylated IκBα (Fig. 5F). Similar effects were observed for NFκB-p65 nuclear translocation (Fig. 5G, fig. S5G) and induction of the NFκB target gene, IL1β (Fig. 5H). Together, these results indicate that NSs co-opts CUL3 to degrade CUL1 and inhibit NFκB signaling (fig. S5H).

## African swine fever virus encodes multiple CUL2/5^ELOB/C^ adaptors with diverse substrates

African Swine Fever Virus (ASFV) is a large, double-stranded DNA virus that infects wild and domestic pigs, causing a severe fever with near-100 % mortality in domestic herds (69, 70). The ASFV gene MGF505–9R induced degradation in a CRL-dependent manner, and a CRISPR screen for required host factors identified CUL5, along with ELOC (*TCEB1*) and ELOB (*TCEB2*) (Fig. 6A-B). MGF505–9R is a member of the MGF505 family of repeat proteins, which are highly duplicated genes (~10–15 copies/genome for each) clustered at the genomic termini. Multiple MGF505 family members, as well as those from the related MGF360 family, scored in the degradation screen (Fig. 6C). AlphaFold 3 predictions suggest MGF505 and MGF360 genes have similar N-terminal domains but differ in the size and sequence of their alpha-helical repeats (fig. S6A). Closer examination of the structurally conserved N-terminal domain of MGF505 and MGF360 genes revealed a conserved BC-box motif, which is used by host ubiquitin ligases to bind to ELOC (Fig. 6D). Consistent with this, degradation by MGF505–9R was blocked by dominant-negative CUL5 (Fig. 6E) or mutation of the BC-box (Fig. 6F). In contrast, MGF360 family members, exemplified by MGF360–10L, induced CRL-dependent protein degradation that was blocked by both dominant-negative CUL2 and dominant-negative CUL5 (fig. S6B-C). MGF505–9R and MGF360–12L bound to CUL5 and CUL2, respectively, and these interactions were dependent on BC-box residues (fig. S6D). Despite conservation of the N-terminal domain, MGF505 and MGF360 family members have divergent sequences in their repeat domains, suggesting the possibility of distinct substrate specificities.

To identify candidate substrates, we compared IP-MS data of MGF505–9R wild-type to *ELOC mutant. As expected, components of the CUL5 complex (CUL5, ELOB, ELOC, RNF7), selectively bound wild-type MGF505–9R relative to the *ELOC mutant. 9R-mediated degradation should reduce the abundance of substrates, resulting in lower spectral counts in wild-type-expressing cells relative to cells expressing the *ELOC mutant. Using this approach, we found ZC3HAV1 (ZAP1), an interferon-inducible CpG-binding antiviral protein and its cofactor, the helicase DHX30, as top hits (Fig. 7A and fig. S7A, Data S3E and S3G) (71–73). Although initially described for RNA viruses, ZC3HAV1 also restricts DNA viruses, including Vaccinia virus (74) and Cytomegalovirus (75). Consistent with its role as a substrate, ZC3HAV1 was robustly down-regulated in porcine WSL-R cells as measured by whole-cell proteomics (Fig. 7B, Data S4C). Among the depleted proteins, we also identified ZC3H7A/B, which are involved in miRNA biogenesis (76), the antiviral and antibacterial ubiquitin ligase RNF213, and caspase 8 (CASP8), an initiator caspase with a central role in extrinsic apoptosis via TNF signaling (fig. S7B) (77). To test the functional consequences of MGF505–9R-mediated degradation, we performed Sendai virus infections of A549 cells treated with IFNβ to upregulate ZC3HAV1 (Fig. 7C-D, fig. S7C-E) (78). MGF505–9R expression enhanced Sendai virus RNA levels, an effect that required CUL5^ELOB/C^ binding and was not observed for MGF505–10R (Fig. 7D).

In addition to MGF505–9R, MGF505–10R scored in the screen with a similar overall fold as 9R and a highly conserved N-terminal domain despite having only 45% overall sequence identity. To identify substrates of MGF505–10R, we applied a similar IP-MS strategy to that described above. Enriched proteins included lysosomal factors involved in autophagy such as FIP200 and its partner ATG13 (79), and components of the GATOR2 complex (MIOS, WDR59, SEH1L, WDR24) (Fig. 7E and S8A, Data S3F-G) (80). Whole-cell proteomics in porcine WSL-R cells revealed loss of FIP200, consistent with its role as a substrate (Fig. 7F). FIP200 degradation by MGF505–10R depended on the BC-box and was blocked by CRL inhibitor MLN4924 (fig. S8B). Despite robust binding to GATOR2 components, we did not observe degradation of these proteins, suggesting that they may not be substrates (fig. S8C).

Given FIP200's role in regulating autophagy, we sought to assess the impact of MGF505–10R expression on starvation-induced autophagy. To do so, we used a previously described autophagy reporter (fig. S8D) (81). Expression of MGF505–10R, but not the *ELOC mutant or MGF505–9R, reduced autophagy flux following amino acid starvation (Fig. 7G). These results were corroborated by autophagy flux measurements using a fluorescent LC3 reporter in which MGF505–10R expression attenuated GFP-LC3 degradation following amino-acid starvation, phenocopying FIP200 loss (fig. S8E) (82). Our findings are consistent with prior reports that autophagy is blocked during ASFV infection (83). Previously, members of the MGF505 and MGF360 families have been shown to have diverse functions (84–91). Our finding that MGF505 and MGF360 family proteins function as CRLs unites these observations and may aid in the development of antivirals and attenuated vaccine strains that exploit this common mechanism of action.

## Discussion

Here, we used a recently constructed viral ORF library to identify viral proteins systematically that target host proteins for degradation. By fusing viral genes to a GFP-specific nanobody, we identified a diverse set of viral ubiquitin ligases that, despite being screened in a substrate-agnostic manner, converged on a core set of substrates involved in immune signaling and antiviral responses.

These viral proteins co-opt the host UPS through diverse strategies, some mirroring host mechanisms and others representing distinct modes of action. We classified the viral degradins into three main categories: (i) Canonical ubiquitin ligases, (ii) Hijackers that redirect host ubiquitin ligases, and (iii) Non-canonical ubiquitin ligases that rewire CRL machinery using atypical mechanisms.

The most straightforward way viruses co-opt the UPS is by acting as canonical ubiquitin ligases that mimic host E3s. One such example is the ASFV MGF505 family members that contain a BC-box consisting of regularly spaced hydrophobic residues on a helix to recruit CUL5^ELOB/C^. Although similar BC-box sequences are found in host proteins, MGF505 family members have a distinct repeat domain not present in host ligases (92), suggesting that this motif may have evolved convergently rather than via gene capture, as seen for poxvirus BTB domain-containing proteins (93). MGF505 family members, as well as the related MGF360 family, have a conserved N-terminus that interacts with degradation machinery and a variable C-terminus that mediates substrate recruitment, allowing the virus to leverage the UPS to regulate diverse cellular processes.

In addition to mimicking host ligases, viral proteins can hijack E3s by binding to host ubiquitin ligases and altering their substrate specificity. We observed such viral hijacking for Teviot virus matrix protein and Adana virus NSs that both degrade JAK1 to regulate cytokine signaling. Despite shared regulation of JAK1, TevPV-M and Adana virus NSs differ considerably in their interactions with the UPS with TevPV-M binding to CUL3^BTBD1^ and Adana virus NSs binding to CUL1^β-TrCP^, essentially acting as an adaptor to endogenous adaptor BTB and F-Box proteins. In both cases, the virus functionalizes a host ligase, facilitating recognition of JAK1 as a neo-substrate.

By mimicking or hijacking host ligases, viruses exploit pre-existing patterns of protein-protein interactions in host cells to execute viral functions. However, viruses can subvert these patterns, assembling complexes that exploit common features of the host UPS. Here we found that Rotavirus A NSP1 uses the CUL2/5 adaptor ELOC in place of a BTB domain to facilitate CUL3 binding and substrate degradation. Like NSP1, the Razdan and Bhanja virus NSs lack a BTB domain but instead use their C-terminal tail to interact with CUL3 at the site where BTB domains typically bind. Thus, despite the stereotypical pattern of Cullins and adaptor proteins observed in host ligases, we found multiple examples of viruses subverting these rules. Unlike host ligases, which typically arise through gene duplication and diversification, viral ligases often emerge de novo, allowing them to explore unconventional modes of interaction including exploiting conserved structural elements to innovate new strategies.

Despite the diverse modes of Cullin recruitment, viral ubiquitin ligases converged on a set of substrates with critical roles in antiviral signaling including the JAK-STAT and NFκB pathways. Although our initial screen was performed in a substrate-agnostic manner by fusing viral proteins to an αGFP nanobody, we identified multiple regulators of JAK1 and CUL1^β–TrCP^. Convergent targeting of immune signaling proteins by viral ubiquitin ligases with diverse mechanisms of degradation highlights the importance of immune signaling in restricting viral infection.

One notable finding from our study is that viruses with diverse host tropisms encode effectors that can modulate immune signaling in human cells. Owing to the diversity of our library, in addition to genes from known human viruses, we identified genes from viruses that do not infect humans as their primary host. Nonetheless, these proteins interacted with components of the human UPS and immune signaling pathways. This suggests that other barriers such as transmission may be more critical for establishing host range while highlighting how adaptation to new hosts can occur rapidly in zoonotic diseases.

In summary, viruses have evolved diverse mechanisms that harness the UPS to inhibit immune signaling and support viral infection. Our approach, combining pooled genetic screens with functional characterization, provides a powerful platform for identifying viral effectors and their host dependencies without requiring live virus infection. This strategy of ectopic viral protein expression enables the study of viruses with diverse host and cell-type tropisms in a single pooled genetic screen, but it does not fully recapitulate viral protein function in the context of a virally infected host cell. However, in conjunction with in-depth mechanistic and functional studies, this approach can be used to discover and characterize viral protein function. While the structural predictions presented in our study are supported by biochemical and functional evidence, definitive validation of these binding interfaces will require future structural studies. Together, our findings reveal potential targets for antiviral intervention and offer insights into viral evolution and immune evasion mechanisms.

## Materials and Methods

### Cell culture

HEK-293T (ATCC:CRL-3216, RRID:CVCL_0063), A375 (ATCC:CRL-1619, RRID:CVCL_0132), and A549 (ATCC: CCL-185, RRID:CVCL_0023) cells were cultured at 37°C, 5% CO_2_ in Dulbecco’s modified Eagle’s medium (DMEM), high glucose, GlutaMAX (ThermoFisher Scientific, 10566016) supplemented with 100 units/mL penicillin-streptomycin (ThermoFisher Scientific, 15070063), and 10% fetal bovine serum (FBS) (Cytiva, SH30088.03). BHK-T7 cells (94) were cultured in DMEM supplemented with 10% FBS, 100 IU/mL penicillin, 100 μg/ml streptomycin, and 0.292 mg/mL L-glutamine, adding 0.3 mg/mL G-418 (Promega) every other passage. MA104 N*V cells (94) were cultured in medium 199 (Sigma-Aldrich) supplemented with 10% FBS, 100 IU/mL penicillin, 100 μg/mL streptomycin, and 0.292 mg/mL L-glutamine, adding 10 μg/mL puromycin (Selleckchem, #S7417) and 10 μg/mL blasticidin (Selleckchem, #S7419) every other passage. HT-29 (ATCC: HTB-38, RRID:CVCL_0320) cells were cultured in advanced DMEM F12 (Gibco #12634010) supplemented with 10% FBS, Penicillin-Streptomycin-Glutamine (Gibco, #10378016), 10 mM HEPES (Gibco, #15630106), Non-Essential Amino Acids Solution (Gibco, #11140050), and 1 mM sodium pyruvate (Gibco, #11360070). WSL-R (RRID:CVCL_0I66) cells were grown at 37°C, 5% CO_2_ in 50% Ham’s F-12 Nutrient Mix medium (ThermoFisher Scientific, 11765054), 50% IMDM (ThermoFisher Scientific, 12440053) supplemented with 10% FBS, 1% Penicillin-Streptomycin, and 1% GlutaMAX (ThermoFisher Scientific, 35050079). For baculovirus generation, Sf9 cells (Expression Systems, 94–001S) were cultured at 27°C in ESF 921 medium (Expression Systems, 96–001-01). For insect cell expression, *Trichoplusia ni* (High Five; Gibco, B85502) cells were cultured at 27°C in SF-4 Baculo Express ICM medium (BioConcept, 9–00F38).

### Lentivirus and retrovirus production

Lentivirus was produced by transfecting 293T cells with transfer plasmid and packaging mix including VSV-G, gag-pol, tat, and rev (2:1:1:1) in PolyJet (SignaGen, SL100688) according to the manufacturer’s instructions. For small-scale virus production, 293T cells were seeded at 0.8 x 10^6^ per well in a 6-well plate. The following day, cells were transfected with 1 μg of transfer plasmid, 1 μg of packaging mix, and 6 μL of PolyJet per well. For large-scale transfection including lentiviral libraries, Lenti-X cells (Takara, 632180) were seeded at 12 x 10^6^ in 15 cm plates. The following day, 6.5 μg transfer plasmid and 6.5 μg packaging mix were combined with 52 μL of PolyJet and added to 15 cm plates. The medium was exchanged 5 h after transfection, and lentivirus-containing supernatant was collected 48 to 72 h after transfection, passed through a 0.45-μm filter or spun down to remove cells, and applied directly to target cells supplemented with 10 μg/mL polybrene (Millipore, TR-1003-G) or concentrated using the Lenti-X concentrator (Takara, 631232).

Retrovirus was produced by transfecting 293T cells with expression plasmid and packaging mix including VSV-G and pUMVC (Addgene #8449 ; RRID:Addgene_8449) at a 1:1 ratio in PolyJet according to the manufacturer’s instructions. 293T cells were seeded at 0.8 x 10^6^ per well in a 6-well plate. The following day, cells were transfected with 1 μg of transfer plasmid, 1 μg of packaging mix, and 6 μL of PolyJet per well. The medium was exchanged 5 h after transfection and retrovirus-containing supernatant was collected 48 h later, spun down to remove cells, and added to target cells for transduction in the presence of 10 μg/mL polybrene.

### Plasmids and cloning

To monitor degradation via αGFPnb fusion, we generated a Gateway-compatible destination vector, pHAGE-TRE-αGFPnb-R-DEST-PGK-puro, that encodes a doxycycline-inducible GFP nanobody (21) in which lysine residues were mutated to arginine to limit autoubiquitination. To optimize degradation by GFP nanobody fusions, we cloned pHAGE-PGK-DsRed-IRES-GFP-EF1a-blast (epCRG.046), a modified version of the GPS3.0 vector (95). Reverse tetracycline-controlled transactivator (rtTA) expression was provided by a modified version of pINDUCER20 (Addgene plasmid # 44012; RRID:Addgene_44012) (96) engineered with mouse CD19 or BFP marker (pINDUCER20-mCD19, pINDUCER20-BFP).

To construct the αGFPnb-vORF library, a barcoded library consisting of ~10,500 vORFs was used. Briefly, this library covers all viral species with human tropism and consists of viral genes up to 570 amino acids in Gateway compatible entry clones (17). For genes longer than this synthesis limit, 570 amino acid fragments with 285 amino acid overlap were used. Each fragment has a unique barcode plus one of five diversification cassettes to improve screening statistics. This library was cloned into pHAGE-TRE-αGFPnb-R-DEST-PGK-puro using LR clonase II (ThermoFisher Scientific, 11791100). Reactions were transformed into ElectroMAX DH10B (ThermoFisher Scientific, 18290015), recovered for 1 h in SOC medium at 37°C, plated on ampicillin-containing LB agar plates, and incubated overnight at 30°C. Bacteria were collected and grown in liquid LB culture supplemented with 100 μg/mL carbenicillin for 4 h at 37°C before performing HiSpeed Maxi preps (Qiagen, 12663).

For validation, individual entry vectors were Gateway cloned into pHAGE-TRE-αGFPnb-R-DEST-PGK-hygro for compatibility with puromycin-resistant lentiviral CRISPR constructs. For follow-up CRISPR screens, vORFs were cloned into the pHAGE-GPS3.0-DEST-hygro vector (95). For IP-MS, whole-cell proteomics, and functional assays, vORFs were Gateway cloned into pHAGE-TRE-3xHA-DEST-PGK-puro or pHAGE-TRE-3xHA-DEST-PGK-blast. As a negative control, an entry clone encoding ffLuc2 was used (Addgene plasmid #162889, RRID:Addgene_162889) (97). Mutants were cloned by Q5 site directed mutagenesis of entry vectors with primers designed using NEBase Changer. Briefly, viral or human entry clones were amplified with Q5 High-Fidelity 2X Master Mix (New England Biolabs, M0492L) with mutagenic primers. The resulting PCR product was treated with Kinase, Ligase, DpnI (KLD) mix (New England Biolabs, M0554S) before transformation into Stbl3 chemically competent cells (Invitrogen, C7373–03). For Rotavirus reverse genetics experiments, we introduced mutations into the pT7-NSP1SA11 (Addgene plasmid #89168, RRID: Addgene_89168) (52) via site directed mutagenesis as described above.

For measurements of substrate stability by flow cytometry, human open reading frames from the Ultimate ORF collection (ThermoFisher Scientific) were cloned into the pHAGE-GPS3.0-DEST-hygro vector (95). Entry clone IDs were as follows: JAK1 (IOH82180), BTRC (β-TrCP1, IOH11366), CUL1 (IOH41991), CTNNB1 (β-catenin, IOH29101). IRF3 was amplified off pTRIP-GFP-IRF3 (Addgene plasmid #127663; RRID:Addgene_127663) (98) with adaptors for pENTR/D-TOPO cloning (Life Technologies, K2400–20). The CUL1^FBXO21^ reporter consisting of a peptide from IFNA8 was described previously (pHAGE-GPS6.0-hygro-P32881_IFNA8_5_M1–15) (67). To monitor induction of interferon expression, we modified pCCL/IFNB1-d2eGFP-3’UTR (Addgene plasmid # 180232; RRID:Addgene_180232) (99), replacing eGFP with mScarlet (epCRG.253 pCCL/IFNB1-d2mScarlet). To monitor autophagy, we used pMRX-IP-HaloTag7-LC3 (Addgene plasmid #184899; RRID:Addgene_184899) (81) and pMXs GFP-LC3-RFP (Addgene plasmid #117413; RRID: Addgene_117413) (82).

To generate ALFA-tagged (100) ELOC, human ELOC 18–112 was PCR amplified off pIVM_26 pCDF-1b DUET ELOBC (Addgene plasmid #204501, RRID:Addgene_204501) (44) with attB adaptors. BP clonase II (ThermoFisher Scientific, 11789100) was used to move ELOC amplicon into pDONR221 (ThermoFisher Scientific, 12536017). This was then shuttled into pRK5-CMV-ALFA-DEST-PGK-TagBFP (epCRG.838) using LR clonase II. To generate FLAG-tagged CUL3, the CUL3 clone from the Ultimate ORF collection (IOH26262) was shuttled into pHAGE-CMV-2xFLAG-DEST-PGK-puro using LR clonase II. To generate ALFA-tagged BTBD1, the BTBD1 clone from the Ultimate ORF collection (IOH21551) was shuttled into pHAGE-PGK-ALFA-DEST-EF1a-blast (epCRG.1030) using LR clonase II. To generate ALFA-tagged CUL1 and JAK1 entry clones from the Ultimate ORF collection (CUL1: IOH41991, JAK1: IOH82180) were cloned into pRK5-CMV-ALFA-DEST-PGK-TagBFP (epCRG.838) using LR clonase II.

For transient transfection of HA-tagged MGF505 family members, vORFs were PCR amplified and cloned into pRK5-HA-SAMTOR (Addgene plasmid #100513, RRID:Addgene_100513) (101) digested with SalI/NotI using Gibson Assembly Master Mix (New England Biolabs, 2611S).

To perturb host degradation pathways, we performed Cas9-mediated mutation and overexpressed dominant-negative Cullin constructs. For individual targeting, guides were cloned into lentiCRISPRv2 (102) with a modified FE hairpin (103). For overexpression of dominant-negative Cullins by transient transfection DN-CUL constructs with a tagBFP marker were used (pHAGE-SFFV-DNCul-FLAG-2A-tagBFP) (104).

For baculoviral insect expression, ELOB, ELOC, Strep-TEV-CUL3 and Strep-TEV-IRF3 were cloned into pAC8 vector. NSP1 (ID: B3SRV2.0.1, ID: Q3ZK61.0.1) and GST-TEV-RBX1 were cloned into pLIB vector. Reagents for neddylation and ubiquitylation were cloned and purified as previously described (105), including pET3a-UB, pGEX4T1–3C-NEDD8, pGEX4T1-TEV-UBE2D3, pGEX4T1-TEV-UBE2M, pET-APPBP1-UBA3, and pLIB-GST-TEV-UBA1.

### Viral ORF degradation screen

A single-cell clone of 293T-pINDUCER20-mCD19 expressing pHAGE-PGK-DsRed-IRES-GFP-EF1a-blast was selected for low DsRed expression and high doxycycline-inducible expression of CD19 using anti-mouse CD19 AF647 (Biolegend, 115522). This cell line was transduced with pHAGE-TRE-αGFPnb-vORF-PGK-puro library at 20–30% transduction efficiency targeting 2,000x representation at the level of ORFs (~132e6 cells) and selected with puromycin. Cells were treated with 200 ng/mL doxycycline (Sigma, D9891–1G) for 16 h and sorted using Sony SH800S cell sorter with 100 μm chip (Sony, LE-C3210), collecting the bottom 1% of cells based on GFP/DsRed ratio. The screen was performed in duplicate with independent infection, selection, and cell sorting.

Genomic DNA from input and sorted samples was extracted using the GeneJET Genomic DNA purification kit (ThermoFisher Scientific, K0722) and amplified using Q5 High-Fidelity 2X Master Mix with primers specific to the region around the barcode that added an adaptor sequence and a 1–7 base pair stagger (5’ only) (106). PCR product was purified using QIAquick PCR purification kit (Qiagen, 28106) and amplified with a second round of PCR to add Illumina adaptors and a sample index. Pooled PCR products were run on a 1% agarose gel, isolated using QIAquick Gel Extraction Kit (Qiagen, 28706) and sequenced with NovaSeq 6000 SP (Illumina) at the Harvard Biopolymers Facility. Illumina sequencing reads were analyzed using Cutadapt followed by Bowtie to identify the barcode. After eliminating barcodes with fewer than 10 reads in either input replicate, sorted samples were compared to unsorted input using MAGeCK (107) to identify enriched barcodes. Open reading frames with fewer than two barcodes were excluded from analysis. Signal peptide-containing vORFs were filtered based on SignalP 5.0 (108) to avoid potential quality control issues associated with N-terminal fusion.

### Validation and ubiquitin CRISPR screens

For individual validation, 293T-pINDUCER20-mCD19 cells expressing pHAGE-PGK-DsRed-IRES-GFP-EF1a-blast were transduced with a single viral ORF in pHAGE-TRE-αGFPnb-DEST-PGK-hygro. Following hygromycin selection, cells were treated for 12 h with 400 ng/mL DOX. In the final 8 h, DOX was diluted to 200 ng/mL and 1 μM MLN4924 (Selleckchem, S7109), TAK-243 (Selleckchem, S8341), or bortezomib (APExBIO, A2614) was added. Following induction and inhibitor treatment, fluorescence was measured using CytoFLEX LX flow cytometer (Beckman Coulter).

For CRISPR screens, vORFs were Gateway cloned into pHAGE-GPS3.0-DEST-hygro vector using LR Clonase II. DsRed IRES GFP-vORF expressing cell lines were transduced at 20–30% transduction efficiency with an sgRNA lentiviral library targeting ~1,500 genes (6 guides/gene) related to the ubiquitin-proteasome system (67) at 500x coverage at the level of guides. Cells were selected in puromycin for 3 d and seeded in complete media for 2 d. Cells were sorted using Sony SH800S cell sorter with 100 μm chip, selecting the top 5% of cells based on GFP/DsRed ratio. Genomic DNA and Illumina libraries were prepared as described above but with modified PCR1 primers to amplify the sgRNA cassette.

For individual Cas9-mediated mutation, validation cell lines were transduced with guides in lentiCRISPRv2 with FE modification and puromycin or blasticidin resistance. 4–7 d after transduction, cells were induced with 200 ng/mL DOX with or without 1 μM MLN4924 for 16 h and fluorescence was measured by flow cytometry.

For dominant-negative Cullin expression, cell lines from individual validation were seeded in 12 well plates at 0.3e6/well and transfected the following day with 300 ng pHAGE-SFFV-DNCul-FLAG-2A-tagBFP or empty vector control and 1μL PolyJet. 5–24 h after transfection, media was exchanged for 200 ng/mL DOX with or without 1 μM MLN4924 and fluorescence was measured 16 h after induction by flow cytometry.

### Immunoprecipitation and whole-cell lysate western blotting

For measurements of whole cell lysate, cells were seeded in 6 well plates and treated with 200 ng/mL DOX with or without 1 μM MLN4924. For signaling assays, cells were treated with 2 nM IFNβ (Peprotech, 300–02BC) or 100 ng/mL TNFα (R&D systems, 210–10-020/CF) for 30 min. Cells were washed once in ice-cold PBS and lysed in 200 μL of RIPA buffer (Boston BioProducts, BP-115X) supplemented with Halt Protease and Phosphatase Inhibitor Cocktail (ThermoFisher Scientific, 78445) for 30 min with end-over-end rotation at 4°C and spun 21,000xg for 12 min at 4°C. Clarified lysate was mixed 1:1 with Tris-Glycine SDS Sample Buffer (ThermoFisher Scientific, LC2676) supplemented with 5% 2-Mercaptoethanol.

For immunoprecipitations, cells were seeded in 10 cm or 15 cm plates and allowed to reach >80% confluency before induction with 200 ng/mL DOX with or without 1 μM inhibitors (MLN4924 or TAK243). For transient expression, cells in 10 cm plates were transfected with 3μg plasmid and 18 μL PolyJet according to manufacturer’s instructions. 12–16 h post-induction or 24 h post-transfection, cells in 10 cm plates were washed in ice-cold PBS and lysed in 500 μL IP-lysis buffer consisting of 25 mM HEPES pH 7.4, 150 mM NaCl, 5 mM EDTA, 1% Triton X-100 supplemented with Halt Protease and Phosphatase Inhibitor. Lysates were rotated end-over-end at 4°C for 30 min and spun 21,000xg for 12 min at 4°C. Clarified lysate was applied to 10 μL pre-washed anti-HA magnetic beads (ThermoFisher Scientific, 88836), anti-FLAG M2 magnetic beads (Millipore Sigma, M8823), or ALFA Selector ST magnetic beads (NanoTag, N1516-L) and incubated for 2 h with end-to-end rotation at 4°C. Beads were washed 3x in 500 μL IP-lysis buffer and eluted with 100 μL of Tris-Glycine SDS Sample Buffer supplemented with 5% 2-Mercaptoethanol (Millipore Sigma, M3148). Samples were boiled for 5 min at 95°C and applied to the magnet before collecting supernatant for western blot analysis.

Whole-cell lysates and immunoprecipitation samples were loaded onto Tris Glycine gels 4–12% (ThermoFisher Scientific, XP04125BOX) or 4–20% (ThermoFisher Scientific, XP04205BOX) with Precision Plus Protein Dual color standard (Bio Rad, 1610394) or PageRuler Plus (ThermoFisher Scientific, 26619). Samples were run for 80 min at 120 volts in Tris-Glycine SDS running buffer (ThermoFisher Scientific, LC26754) and transferred onto 0.2 μm Nitrocellulose membranes (Bio Rad, 1704158) using the Trans-Blot Turbo Transfer system (Bio Rad). Membranes were treated with blocking buffer consisting of 5% milk (LabScientific, M0842) diluted in 20 mM Tris pH 7.4, 150 mM NaCl, 1% Tween 20 (TBS-T). After 30-min blocking with gentle rocking at room temperature, membranes were washed twice with TBS-T and incubated with 1:2,000 primary antibody diluted in TBS-T with 5% bovine serum albumin (w/v) and 0.02% sodium azide or blocking buffer overnight at 4°C with gentle agitation.

After primary antibody incubation, membranes were rinsed quickly in TBS-T followed by three 5 min washes. Secondary antibodies were diluted in blocking buffer and incubated for 1 h at room temperature with gentle rocking. For rabbit antibodies, 1:5,000 dilution of Goat anti-Rabbit IgG HRP (Invitrogen, 31460 or Cell Signaling Technologies, 7074S) was used. For mouse antibodies, 1:2,000–1:5,000 dilution of Horse anti-Mouse HRP (Cell Signaling Technologies, 7076S) or 1:5,000 IRDye^®^ 680RD Goat anti-Mouse IgG Secondary Antibody (Licor, 926–68070) in Intercept (PBS) Blocking Buffer (Licor, 927–70001) was used. After secondary incubation, membranes were washed as described above and incubated with Western Lightning Plus ECL (Perkin Elmer, NEL104001EA) or SuperSignal West Femto Maximum Sensitivity Substrate (ThermoFisher Scientific, 34096) for 5 min before exposures were collected with high sensitivity autoradiography film (Denville Scientific, E3212), Odyssey Imager (Licor), or ChemiDoc MP imaging system.

### Immunoprecipitation mass-spectrometry

293T-pINDUCER20 cells were transduced with viral ORFs in pHAGE-TRE-3xHA-DEST-PGK-puro, selected with puromycin, and expanded into 5x15cm plates. After reaching >80% confluency, cells were induced with 200 ng/mL DOX with or without 1 μM inhibitors (MLN4924 or TAK243) overnight. Each 15 cm plate was lysed in 1 mL IP-lysis buffer, incubated with end-over-end rotation at 4°C for 30 min, and spun 21,000xg for 20 min at 4°C. Clarified lysate was applied to 100 μL (20 μL/15 cm plate) of pre-washed anti-HA magnetic beads and incubated for 2 h with end-to-end rotation at 4°C. Beads were washed with 3 mL of IP-lysis buffer followed by three 1 mL washes. Beads were incubated with 100 μL 50 mM Tris pH 7.4 in 10% SDS at 95°C for 5 min to elute proteins. Supernatants were collected from the magnet, reduced with 5 mM TCEP (ThermoFisher Scientific, 77720) for 15 min at 55°C and alkylated with 20 mM Iodoacetamide for 30 min at room temperature protected from light. Samples were acidified with 2.5% phosphoric acid, quenched by 10-fold dilution with 100 mM Tris pH 7.4 in 90% methanol and passed through S-Trap microcolumns (Protifi, C02-micro-10). Columns were washed 3 times with quench buffer and incubated with 2 μg of trypsin (Promega, V5113) in 20 μL ammonium bicarbonate pH 8.0 overnight at 37°C. Peptides were eluted by successive application of ammonium bicarbonate pH 8.0, 0.2% formic acid, 50% acetonitrile, spinning 1 min at 4,000xg between each step. Peptides were dried under reduced pressure using SpeedVac, resuspended in 30 μL 0.1% formic acid and analyzed by LC-MS/MS on Vanquish Flex LC with Acclaim 120 C18 2.2 μM 120 Å 2.1 x 100 mm column (ThermoFisher Scientific, 068982) and Q Exactive mass spectrometer (ThermoFisher Scientific).

To analyze peptide enrichment, the human proteome was downloaded from UniProt (UP000005640) and supplemented with HA-tagged viral proteins in fasta format. Fragpipe v18.0 (109) was used to perform peptide calling with a 10 PPM tolerance for precursor ions and 0.04 Da fragment mass tolerance allowing for 2 missed trypsin cleavages. Common 293T contaminants that appeared in multiple experiments independent of bait expression were filtered out (see Data S3G).

### Protein expression and cryo-electron microscopy

Individual baculovirus preparations for Strep-TEV-CUL3, ELOB, ELOC, GST-TEV-RBX1, and NSP1 (ID: B3SRV2.0.1 or Q3ZK61.0.1) were generated in *Spodoptera frugiperda* cells. *Trichoplusia ni* cells were coinfected with all viruses and the complex was purified by GST affinity, followed by TEV cleavage overnight at 4°C, ion exchange chromatography, and size exclusion chromatography into a buffer consisting of 25 mM HEPES, 200 mM NaCl, 1 mM TCEP, pH 7.5.

NSP1-ELOB-ELOC-CUL3-RBX1 complex was diluted to 4 μM immediately before application to grids. Quantifoil Cu 1.2/1.3 grids were glow discharged at 15 mA for 20 seconds, and 4 μL of sample was applied and immediately plunge frozen into liquid ethane using a Leica EM GP1 plunge freezer, with chamber humidity at 90% and 10°C. The dataset was collected on a ThermoFisher Titan Krios at 300 kV equipped with a Falcon 4i direct electron detector by EPU. Movies were collected with a total dose of 50 e^-^/Å^2^ over 80 frames, at 0.737 Å/pixel with a nominal magnification of 165,000x, defocus range of -0.8 μm to -2.0 μm, and at -15° and -30° stage tilt to compensate for particle orientation bias. 12,479 movies were collected and the dataset was processed in cryoSPARC v4.7 (fig. S1D).

Models were built using a local refinement map focusing on NSP1-ELOB-ELOC and an N-terminal portion of CUL3. An initial model was generated in AlphaFold 3 and ChimeraX was used to manually fit each domain of the proteins to accommodate differences seen in comparison to the AlphaFold generated model. COOT was used to build the model manually followed by phenix.refine for refinement. The final model was generated using iterations of phenix.refine and COOT inspection.

### In vitro ubiquitination assay

Purified NSP1-ELOB-ELOC-CUL3-RBX1 complex was NEDDylated by incubating 5 μM NSP1 complex, 1 μM UBE2M, 0.2 μM APPBP1-UBA3, and 15 μM NEDD8 at room temperature for 10 minutes in 25 mM HEPES, 100 mM NaCl, 10 mM MgCl_2_, 5 mM ATP, pH 7.5. Reaction was quenched by adding 20 mM DTT and purified by size exclusion chromatography in 25 mM HEPES, 200 mM NaCl, 1 mM TCEP, pH 7.5. For ubiquitination, 500 nM Strep-IRF3 was incubated with equimolar NSP1-ELOB-ELOC-CUL3-RBX1 with or without NEDDylation on ice for 20 minutes. Reaction was initiated in 25 mM HEPES, 100 mM NaCl, 10 mM MgCl_2_, 5 mM ATP, pH 7.5 by adding additional 2 μM UBE2D, 0.2 μM UBA1, and 60 μM ubiquitin at room temperature. Samples were taken at each time point and quenched with SDS sample buffer and separated by SDS-PAGE. The assay was analyzed by Western blot using a Strep-tag II antibody (1:4000, AbCam, ab76950) and an Anti-Rabbit IgG IRDye 800CW (1:4000, Licor, #92632211). The membrane was imaged on the LiCor Odyssey LCx.

### RNA extraction and quantitative PCR

For analysis of IL1β expression, A375-pINDUCER20 cells were transduced with vORFs in pHAGE-TRE-3xHA-DEST-PGK-puro. Cells were seeded in 12 well plates and induced with 200 ng/mL DOX for 16 h followed by 4 h in 100 ng/mL TNFα. Cells were washed once in PBS and RNA was extracted using the Direct-zol RNA Miniprep kit (Zymo Research, R2072). cDNA was generated using iScript cDNA synthesis kit (Bio Rad, 1708891) and quantitative PCR was performed using SYBR Green qPCR master mix (GLPBIO, GK10002) with primers against ACTB (Integrated DNA technologies, Hs.PT.39a.22214847) or IL1B (Integrated DNA technologies, Hs.PT.58.1518186).

For induction of ZC3HAV1 following IFNβ treatment, A549 cells were treated with 5 nM IFNβ for 24 h followed by 18 h incubation in complete media. Cells were washed with PBS prior to RNA isolation, cDNA generation, and qPCR for ZC3HAV1 (Integrated DNA technologies, Hs.PT.58.1926165) and ACTB.

### Rotavirus reverse genetics and infection assays

Recombinant simian Rotavirus SA11 was generated using an optimized, entirely plasmid-based reverse genetics system as previously described (94). Briefly, 0.4 μg each of pT7-SA11-VP1, -VP2, -VP3, -VP4, -VP6, -VP7, -NSP1 (wild-type or mutants *CUL3: L185R/L186R, *ELOC: F211R), -NSP3, and -NSP4; 1.2 μg each of pT7-SA11-NSP2 and -NSP5; 0.8 μg of the helper plasmid C3P3-G1; and 14 μl TransIT-LT1 (Mirus, MIR 2304) were mixed and transfected into BHK-T7 cells in 12-well plates. To generate the pT7-SA11-ΔNSP1 plasmid, the NSP1 coding sequence was replaced with EGFP as previously described (110). At 18 h post-transfection, cells were washed twice with FBS-free DMEM and incubated with 800 μL fresh FBS-free DMEM. Twenty-four hours later, 0.5 × 10^5^ MA104 N*V cells in 200 μL FBS-free DMEM containing 2.5 μg/mL porcine pancreatic type IX-S trypsin (Sigma-Aldrich) were added, and cocultures were maintained for an additional 3 days. Cells were then subjected to three freeze-thaw cycles. Rescued viruses were amplified in MA104 cells in 6-well plates to generate virus stocks.

For substrate degradation assays, MA104 cells in 24-well plates were infected with rSA11 or rSA11-NSP1 mutants at a multiplicity of infection (MOI) of 5 for 8 h. Cells were washed twice with ice-cold PBS and lysed in RIPA buffer supplemented with protease inhibitor cocktail (Thermo Scientific) for 30 min at 4°C. Lysates were clarified by centrifugation at 12,000xg for 10 min at 4°C, resolved on 4–15% SDS-PAGE gels, and transferred to 0.45-μm nitrocellulose membranes (Bio-Rad). Following incubation with primary and secondary antibodies, membranes were visualized using the Clarity Western ECL substrate (Bio-Rad).

For viral titer measurements, HT-29 cells (2 × 10^5^ cells/ml) were seeded in 24-well plates and infected with SA11 at a multiplicity of infection (MOI) of 0.01. As a control, the ΔNSP1 strain was used. After infection, cells were washed five times with PBS to remove unbound virus and incubated in serum-free medium containing 0.5 μg/mL trypsin. Infected cells were harvested at 1 h and 24 h post-infection and subjected to three freeze-thaw cycles prior to viral titer determination. Viral titers were measured in MA104 cells by focus-forming unit (FFU) assay. Briefly, virus samples were serially diluted 5- or 10-fold and incubated with a monolayer of MA104 cells seeded in 96 well plates for 14 h at 37°C. Following infection, cells were fixed with 10% formalin, permeabilized with 1% Triton X-100, and incubated with anti-rotavirus capsid mouse monoclonal antibody and anti-mouse HRP-linked antibodies. Foci were detected using 3-amino-9-ethylcarbazole HRP substrate (Vector Laboratories, SK-4200) and stopped by washing twice with PBS.

### Sendai virus infections

For IFNβ reporter induction, A375-pINDUCER20 cells were transduced with pCCL/IFNB1-d2mScarlet and single-cell cloned. Cells were transduced with vORFs in pHAGE-TRE-3xHA-DEST-PGK-puro, selected with puromycin, and seeded for SeV infection in 96 well flat bottom plates. Cells were treated with 200 ng/mL DOX for 24 h and infected with a 1:100 dilution of Sendai virus (ATCC, VR-907) for 14 h prior to mScarlet measurements by flow cytometry.

For measurements of viral load, A549-pINDUCER20 cells were transduced with lentiviral CRISPR constructs or vORFs in pHAGE-TRE-3xHA-DEST-PGK-puro. Selected cells were seeded in 12 well plates at 100,000 cells/well and treated with 200 ng/mL DOX and 5 nM IFNβ for 24 h. Cells were overlaid with 1:166 Sendai virus in serum-free DMEM for 1 h prior to dilution in complete DMEM to 1:500 final concentration. 18 h after infection, cells were washed twice in PBS and RNA was purified using the Direct-zol RNA Miniprep kit and analyzed for expression of SeV-N using previously reported primers (78) relative to ACTB.

### Structural predictions

AlphaFold structural predictions were performed using the AlphaFold 3 server (111) and visualized using ChimeraX (112). N- and C-terminal regions with pLDDT < 50 were omitted from figures of protein complexes.

### Phylogenetic analysis

Position-Specific Iterated BLAST (PSI-BLAST) (113) was performed using the Swiss-Prot database or non-redundant protein sequence database as indicated. Hits were filtered to remove incomplete sequences and clustered using MMseqs2 (114) to prevent redundancy (>98% sequence identity). Unique hits were aligned using Clustal Omega (115, 116) and visualized using Jalview (117) and Interactive Tree of Life (118).

### Substrate stability and cellular ubiquitination assays

For substrate stability measurements, human ORFs expressed in pHAGE-GPS3.0-DEST-hygro were introduced by lentiviral transduction into 293T-pINDUCER20 cells that were subsequently infected with vORFs in pHAGE-TRE-3x-HA-DEST-PGK-puro. Following drug selection, cells were treated with 200 ng/mL DOX for 16 h and fluorescence was measured by flow cytometry.

For cellular ubiquitination assays, 293T-pINDUCER20 cells harboring pHAGE-TRE-3x-HA-vORF-PGK-puro were seeded in 10 cm plates and transfected with 6 μg plasmid encoding human ORFs expressed in pHAGE-GPS3.0-DEST-hygro. 5 h post transfection, media was exchanged for 200 ng/mL DOX for 16 h. In the final hour, 1 μM bortezomib was added to prevent degradation. Cells were lysed with 1 mL lysis buffer containing 50 mM Tris pH7.5, 150 mM NaCl, 1 mM EDTA, 0.5% Triton X-100, 0.7% (w/v) N-ethylmaleimide, and Halt Protease and Phosphatase Inhibitor Cocktail for 30 min at 4°C. Lysates were spun down for 12 min at 21,000xg and clarified lysates were incubated with 10 μL ChromoTek GFP-Trap Magnetic Agarose (Proteintech, gtma-20) for 1 h at 4°C. Beads were washed once with lysis buffer followed by 3x750 μL washes in 8M urea, 1% SDS in PBS and one 750 μL wash in 1% SDS in PBS, spinning 1 min at 2,000xg between washes. After a final wash in lysis buffer, GFP was eluted in 100 μL Tris-Glycine SDS Sample Buffer containing 5% βME with 10 min incubation at 95°C. Samples were placed on a magnetic stand, and supernatant was transferred to new tubes. Western blots were performed as described above with primary antibodies against GFP (Santa Cruz, sc-9996) and ubiquitin (Cell Signaling Technologies, 58395S).

### Whole-cell proteomics

For whole-cell proteomics, A375-pINDUCER20, 293T-pINDUCER20, or WSL-R-pINDUCER20 cells were transduced with vORFs or luciferase (ffLuc2) control in pHAGE-TRE-3xHA-DEST-PGK-puro. Following puromycin selection, cells were seeded in triplicate on 10 cm plates and allowed to reach >80% confluency before induction with 200 ng/mL DOX for 16 h. Cells were washed in ice-cold PBS and lysed in 8 M urea, 200 mM EPPS pH 8.5 supplemented with protease inhibitor (Pierce, A32953) by passing through a 1.5-inch 21G needle for 10 strokes. Protein concentration was quantified using Pierce BCA assay (ThermoFisher Scientific, 23227) and normalized to 1 mg/mL. 100 μL of normalized lysate was reduced with 5 mM TCEP for 15 min, alkylated with 10 mM iodoacetamide for 30 min in the dark, and quenched with 5 mM dithiothreitol (DTT) for 15 min. Protein was extracted via methanol-chloroform precipitation, vortexing for 5 sec between each addition: 400 μL methanol, 100 μL chloroform, 300 μL water. The samples were centrifuged for 1 min at 14,000xg before removal of organic and aqueous phases. The samples were washed once with 400 μL methanol and centrifuged at 21,000xg for 2 min. Pellets were resuspended in 200 mM EPPS pH 8.5 and treated with 1 μg Lys-C protease (FUJIFILM, 121–05063) overnight at room temperature. The following day, 1 μg trypsin (ThermoFisher Scientific, 90305) was added for 6 h at 37°C. Following digestion, samples were labeled with 200 μg TMT reagent (ThermoFisher Scientific, A52045) in 30% acetonitrile for 1 h at room temperature, quenched with 0.3% hydroxylamine, desalted with C18 solid-phase extraction (Waters, WAT054925), and centrifuged under reduced pressure to dry.

The pooled TMT-labeled peptides were fractionated using an Agilent 1260 HPLC system. Peptides were separated over a 560-min linear gradient from 5% to 35% acetonitrile in 10 mM ammonium bicarbonate (pH 8) at a flow rate of 0.25 mL/min, utilizing an Agilent 300 Extend C18 column (3.5 μm particles, 2.1 mm ID, 25 cm length). The peptide mixture was divided into 96 fractions that were then combined into 24 super-fractions for FAIMS-MS/MS analysis. Each super-fraction was acidified with 1% formic acid, concentrated to near dryness via vacuum centrifugation, desalted using StageTips, dried again, and reconstituted in 5% acetonitrile with 5% formic acid for LC-MS/MS analysis.

Mass spectrometric data were collected on an Orbitrap Ascend mass spectrometer coupled to a Vanquish Neo UHPLC for twelve non-adjacent super-fractions. Approximately 1 μg of peptide was separated at a flow rate of 450 nL/min on a 100 μm capillary column that was packed with 35 cm of Accucore 150 resin (2.6 μm, 150 Å; ThermoFisher Scientific). The scan sequence began with an MS1 spectrum using the following parameters for Orbitrap analysis: resolution 60,000, 350–1350 Th, automatic gain control (AGC) target of 100%, maximum injection time 50 ms. Data were acquired for 90 min per fraction. The hrMS2 stage consisted of fragmentation by higher energy collisional dissociation (HCD, normalized collision energy 36%) and analysis using the Orbitrap (AGC 200%, maximum injection time 120 ms, isolation window 0.6 Th, resolution 45,000). Data were acquired using the FAIMSpro interface with dispersion voltage (DV) of 5,000V, the compensation voltages (CVs) of -40V, -60V, and -70V, and TopSpeed parameter of 1 sec per CV. Data analysis was performed as described previously (119).

### Immunofluorescence

For analysis of NFκB nuclear translocation, A375-pINDUCER20 cells were transduced with vORFs in pHAGE-TRE-3xHA-DEST-PGK-puro. Cells seeded on Poly-L-Lysine coated coverslips (Fisher Scientific, 08–774-383) were treated with 200 ng/mL DOX for 16 h followed by 30 min with 100 ng/mL TNFα. Coverslips were washed once in PBS and fixed in 4% formaldehyde (ThermoFisher Scientific, 28906) in PBS for 10 min at room temperature. Coverslips were washed twice in PBS, incubating 5 min between washes, and permeabilized for 30 min at room temperature in blocking solution consisting of 1 mg/mL bovine serum albumin (Millipore Sigma, A3294), 3% goat serum (Cell Signaling Technologies, 5425), 0.1% Triton X-100, 1 mM EDTA pH 8.0 diluted in PBS. 1:500 αNFκB-p65 Rabbit mAb (Cell Signaling Technology, 8242S) primary antibody was added overnight at 4°C. Coverslips were washed 3 times in PBS, incubating 5 min between washes. 1:500 Goat anti-rabbit IgG AF647 (ThermoFisher Scientific, A32733) was added in blocking buffer for 1 h at room temperature. Coverslips were washed 3 times in PBS, incubating 5 min between washes. In the second wash, 500 ng/mL DAPI (Millipore Sigma, D9542) was added. Coverslips were mounted on glass slides using ProLong Gold Antifade Mountant (ThermoFisher Scientific, P36934) and imaged on Axio Observer 7 (Zeiss) with Plan-Apochromat 63x/1.40 Oil DIC M27 objective (Zeiss, 420782–9900-000), Colibri 7 Type R[G/Y]CBV-UV light source (Zeiss, 423052–9741-000), and Axiocam 705 mono camera (Zeiss, 426560–9060-000). Images were processed using FIJI (120) and analyzed using CellProfiler (121) to quantify nuclear NFκB p65.

### Autophagy reporter assays

293T-pINDUCER20 expressing pMRX-IP-HaloTag7-LC3 were transduced with lentiviral CRISPR constructs or vORFs in pHAGE-TRE-3xHA-DEST-PGK-blast. For vORF expression, transduced cells were induced with 200 ng/mL DOX for 16 h and labeled with 100 nM HaloTag TMR Ligand (Promega, G8251) for 20 min. Cells were washed once and starved in DMEM (High Glucose) with Sodium Pyruvate, without Amino Acids (FUJIFILM, 048–33575) for 6 h. Cells were washed once with PBS, lysed in 200 μL RIPA buffer, and analyzed by western blot as described above.

For the autophagy flux fluorescent reporter assay, 293T-pINDUCER20 cells expressing pMXs GFP-LC3-RFP were transduced with lentiviral CRISPR constructs or vORFs in pHAGE-TRE-3xHA-DEST-PGK-puro. Following puromycin selection, cells were treated with 200 ng/mL DOX for 24 hours followed by starvation in DMEM without amino acids over a time course. Following starvation, fluorescence intensity was measured by flow cytometry.

## Supplementary Material

DataS4_WCP

DataS7-aaSeq

DataS8_rawWB

DataS3-IP-MS

DataS5-abClones

DataS1-aGFPnbScreen

DataS2-CRISPR_screens

DataS6-primers-guides

Glassman_2026_supplement

scienceaec6299_mdar_reproducibility_checklist

List of Supplementary Materials

Figs. S1 to S8

Tables S1

Data S1-S8

MDAR Reproducibility Checklist

## Figures and Tables

**Fig. 1.**
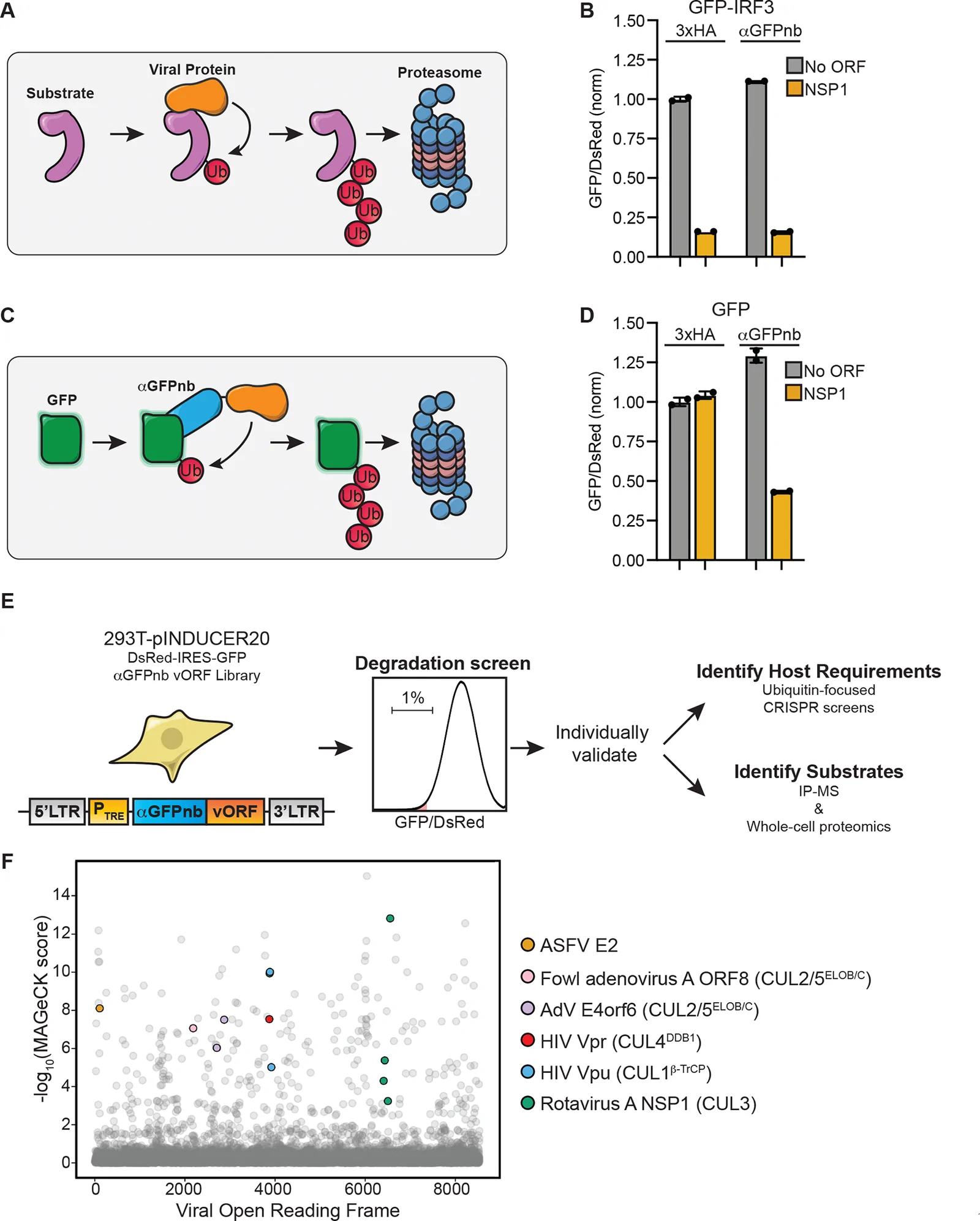
A pooled genetic screen identifies viral regulators of protein stability. (A-D) anti-GFP nanobody (αGFPnb) fusion enables substrate-agnostic tracking of protein degradation. (A) General schematic of a viral ubiquitin ligase. (B) Rotavirus A NSP1 (ID: Q3ZK61.0.1) degrades IRF3. 293T-pINDUCER20 cells expressing GPS-IRF3 (DsRed IRES GFP-IRF3) and a tetracycline response element (TRE)-driven NSP1 with N-terminal 3xHA or αGFPnb fusion were treated with 200 ng/mL doxycycline (DOX) for 16 h and GFP/DsRed ratio was measured by flow cytometry. Data show mean ± SD of duplicate measurements and are representative of two independent experiments. (C) Schematic of αGFPnb-mediated degradation. (D) αGFPnb fusion enables NSP1 degradation of GFP. 293T-pINDUCER20 cells expressing GPS reporter (DsRed IRES GFP) were analyzed as described in B. (E) Workflow for discovery and characterization of viral ubiquitin ligases. Immunoprecipitation-mass spectrometry (IP-MS). (F) Results from αGFPnb-vORF degradation screen. Significance was calculated by comparing barcode abundance in the bottom 1% GFP/DsRed cells relative to input using MAGeCK. Representative genes are indicated as colored dots. The presence of multiple dots indicates similar viral genes scoring across species or strains. The screen was performed in duplicate with independent infection, selection, and sorting. See also Data S1.

**Fig. 2.**
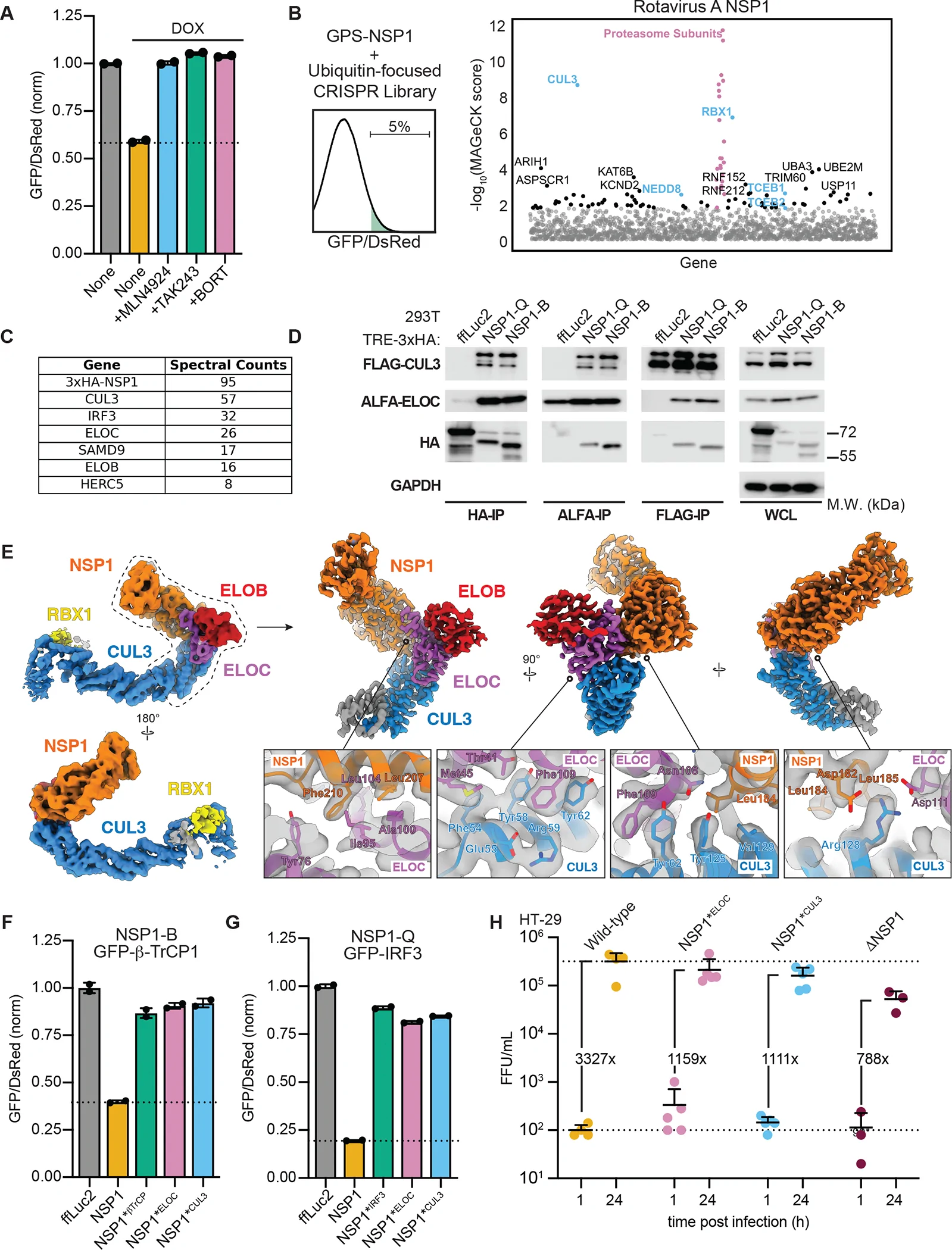
Rotavirus A NSP1 co-opts CUL3^ELOB/C^ to suppress antiviral RNA signaling. (A) Rotavirus A NSP1-mediated degradation requires Cullin-RING ligase activity. GPS reporter cells harboring TRE-αGFPnb-NSP1 (ID: Q3ZK61.0.1) were treated with 200 ng/mL doxycycline (DOX) for 4 h prior to addition of 1 μM inhibitor for the final 8 h (BORT: bortezomib). Fluorescence was measured in duplicate by flow cytometry and displayed as mean ± SD. (B) NSP1 degradation requires a CUL3 complex. Ubiquitin-focused CRISPR screen in GPS-NSP1 (DsRed IRES GFP-Q3ZK61.0.1) cells. See also Data S2A-B. (C) NSP1 host factor interactions by immunoprecipitation-mass spectrometry (IP-MS). TRE-3xHA-NSP1 expression was induced with DOX in the presence of MLN4924 for 16 h. Total spectral counts from a single experiment are shown after filtering common contaminants. See also Data S3A and S3G. (D) NSP1 nucleates a CUL3^ELOB/C^ complex. Cells were transfected with TRE-3xHA-ffLuc2 or NSP1 (ID: Q3ZK61.0.1 or B3SRV2.0.1), CMV-ALFA-ELOC, and CMV-2xFLAG-CUL3 in the presence of DOX prior to affinity purification. (E) Cryo-electron microscopy (cryo-EM) structure of NSP1-CUL3^ELOB/C^ with highlighted region selected for local refinement. (F-G) NSP1-mediated degradation depends on substrate and CUL3^ELOB/C^-binding. GPS-β-TrCP1 (DsRed IRES GFP-BTRC) cells harboring TRE-NSP1 (ID: B3SRV2.0.1) and mutants (*β-TrCP: S480A/S483A, *ELOC: F210R, *CUL3: L184R/L185R) (F) or GPS-IRF3 (DsRed IRES GFP-IRF3) cells harboring TRE-NSP1 (ID: Q3ZK61.0.1) and mutants (*IRF3: L483R, *ELOC: F210R, *CUL3: L184R/L185R) (G) were analyzed as in A. (H) Rotavirus rSA11 replication in interferon-competent HT-29 cells infected at a multiplicity of infection (MOI) of 0.01 and analyzed at time of harvest by focus-forming unit (FFU) assay. Dots represent paired measurements of individual viral stocks for which infection was detectable at both time points. See also fig. S2H. Results are representative of two or more independent experiments unless otherwise noted.

**Fig. 3.**
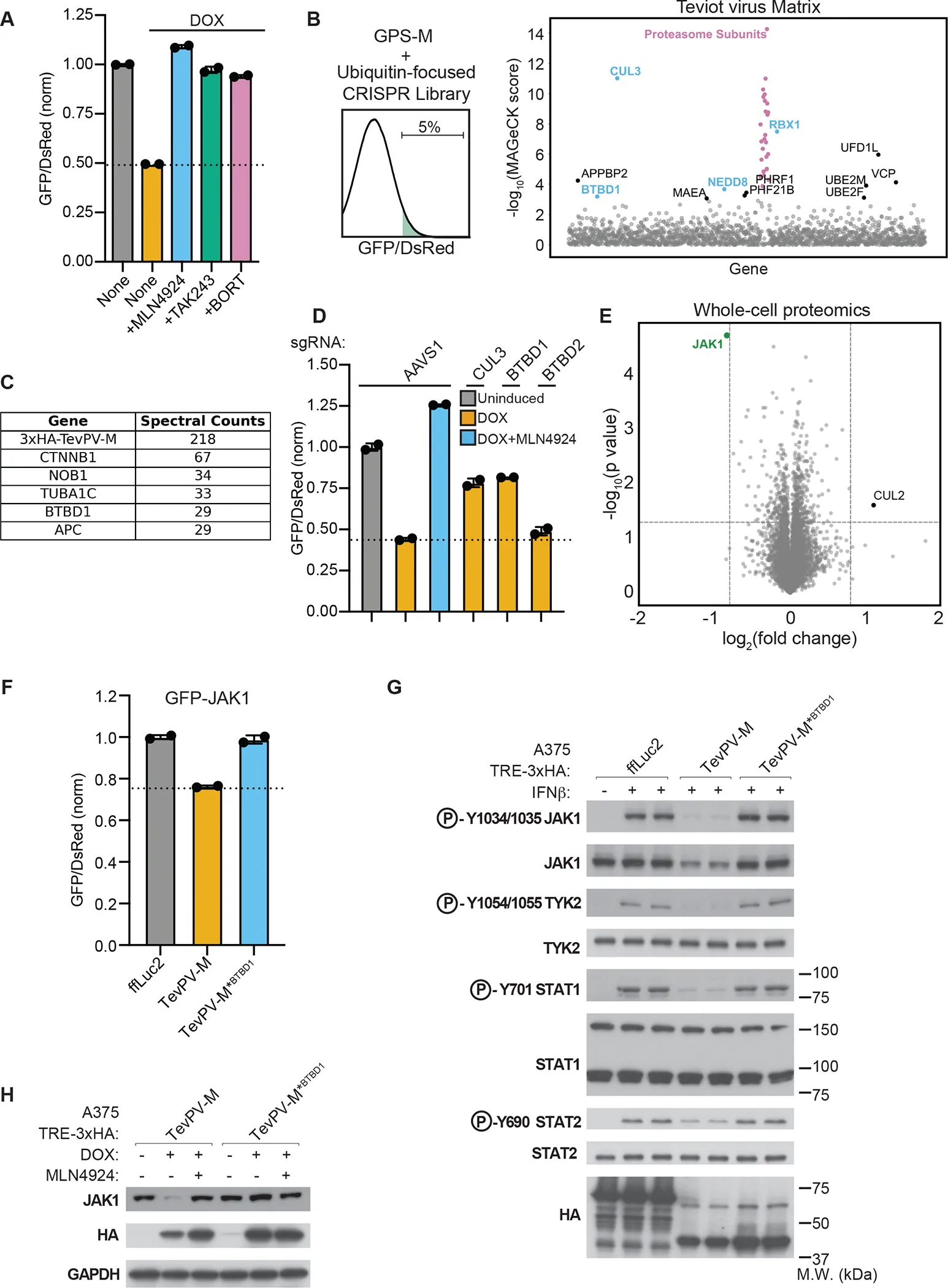
Teviot virus matrix protein (TevPV-M) recruits CUL3^BTBD1^ to degrade JAK1. (A) TevPV-M-mediated degradation requires Cullin-RING ligase activity. GPS reporter cells harboring TRE-αGFPnb-TevPV-M (ID: YP_009176989.1.1) were treated with 200 ng/mL doxycycline (DOX) for 4 h prior to addition of 1 μM inhibitor for the final 8 h (BORT: bortezomib). Fluorescence was measured in duplicate by flow cytometry and displayed as mean ± SD. (B) TevPV-M degradation depends on CUL3^BTBD1^. Ubiquitin-focused CRISPR screen in GPS-TevPV-M (DsRed IRES GFP-YP_009176989.1.1) cells. See also Data S2C-D. (C) TevPV-M interaction mapping by immunoprecipitation-mass spectrometry (IP-MS). TRE-3xHA-TevPVM expression was induced with DOX in the presence of TAK243 for 12 h. Total spectral counts from a single experiment are shown after filtering to remove common contaminants across experiments. See also Data S3B and S3G. (D) TevPV-M-mediated degradation is dependent on CUL3^BTBD1^. GPS reporter cells transduced with lentiCRISPR constructs were analyzed as described in A. (E) Whole-cell proteomics of TevPV-M expression in A375 cells relative to luciferase control for a single experiment performed in triplicate. Proteins consistently enriched in the ffLuc2 control were omitted from the plot. See also Data S4A. (F) TevPV-M degrades JAK1. GPS-JAK1 (DsRed IRES GFP-JAK1) cells were transduced with TevPV-M or mutant (*BTBD1:L196E/I199E/P297F) and treated with DOX for 16 h prior to analysis as described in A. See also fig. S3A-B. (G) TevPV-M attenuates IFNβ signaling. A375 cells were transduced with constructs described in F, induced with DOX and stimulated for 30 min with 2 nM IFNβ. (H) TevPV-M expression inversely correlates with JAK1 abundance. Cells from G were treated as indicated for 16 h. See also fig. S3. Experimental results are representative of two or more independent experiments unless otherwise indicated.

**Fig. 4.**
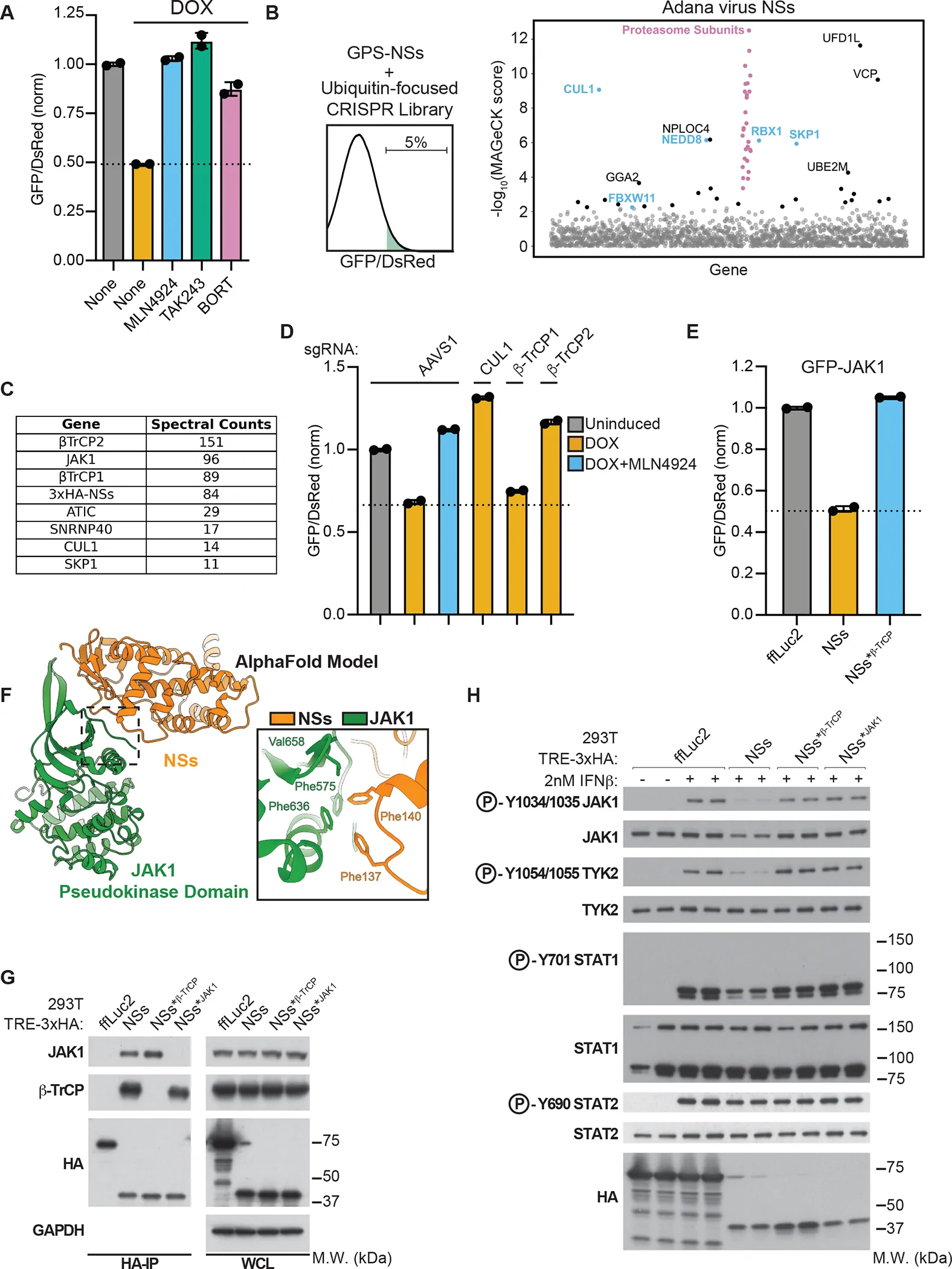
Adana virus NSs recruits CUL1^β-TrCP^ to degrade JAK1. (A) Adana virus NSs-mediated degradation requires Cullin-RING ligase activity. GPS reporter cells harboring TRE-αGFPnb-NSs (ID: YP_009227129.1.1) were treated with 200 ng/mL doxycycline (DOX) for 4 h prior to addition of 1 μM inhibitor for the final 8 h (BORT: bortezomib). Fluorescence was measured in duplicate by flow cytometry and displayed as mean ± SD. (B) NSs degradation depends on CUL1^β-TrCP2^. Ubiquitin-focused CRISPR screen in GPS-NSs (DsRed IRES GFP-YP_009227129.1.1) cells. See also Data S2E-F. (C) NSs interaction mapping by immunoprecipitation-mass spectrometry (IP-MS). TRE-3xHA-NSs expression was induced with DOX in the presence of MLN4924 for 16 h prior to HA immunoprecipitation. Total spectral counts from a single experiment are shown after filtering to remove common contaminants across experiments. See also Data S3C and S3G. (D) NSs-mediated degradation is dependent on CUL1^β-TrCP2^. GPS reporter cells from A were transduced with CRISPR-Cas9 constructs targeting the indicated genes (β-TrCP1: *BTRC*, β-TrCP2: *FBXW11*) and analyzed as described in A. (E) NSs expression reduces JAK1 levels. GPS-JAK1 (DsRed IRES GFP-JAK1) cells harboring NSs wildtype or mutant (*β-TrCP: S259A/S262A/T263A) were treated with DOX for 16 h and analyzed as in A. (F) AlphaFold 3 prediction of NSs-JAK1 pseudokinase domain complex (ipTM = 0.64). See also, fig. S4A-B. (G) Separation of function mutations uncouple JAK1 and β-TrCP binding. Cells expressing NSs or mutants (*β-TrCP and *JAK1:F137A/F140A) were treated with DOX + MLN4924 for 16 h before HA-IP and western blotting. (H) NSs suppression of IFNβ signaling requires β-TrCP and JAK1 binding. A375 cells were transduced with constructs as described in G and induced with DOX for 24 h prior to 30 min stimulation with 2 nM IFNβ. See also fig. S4. Experimental results are representative of two or more independent experiments unless otherwise indicated.

**Fig. 5.**
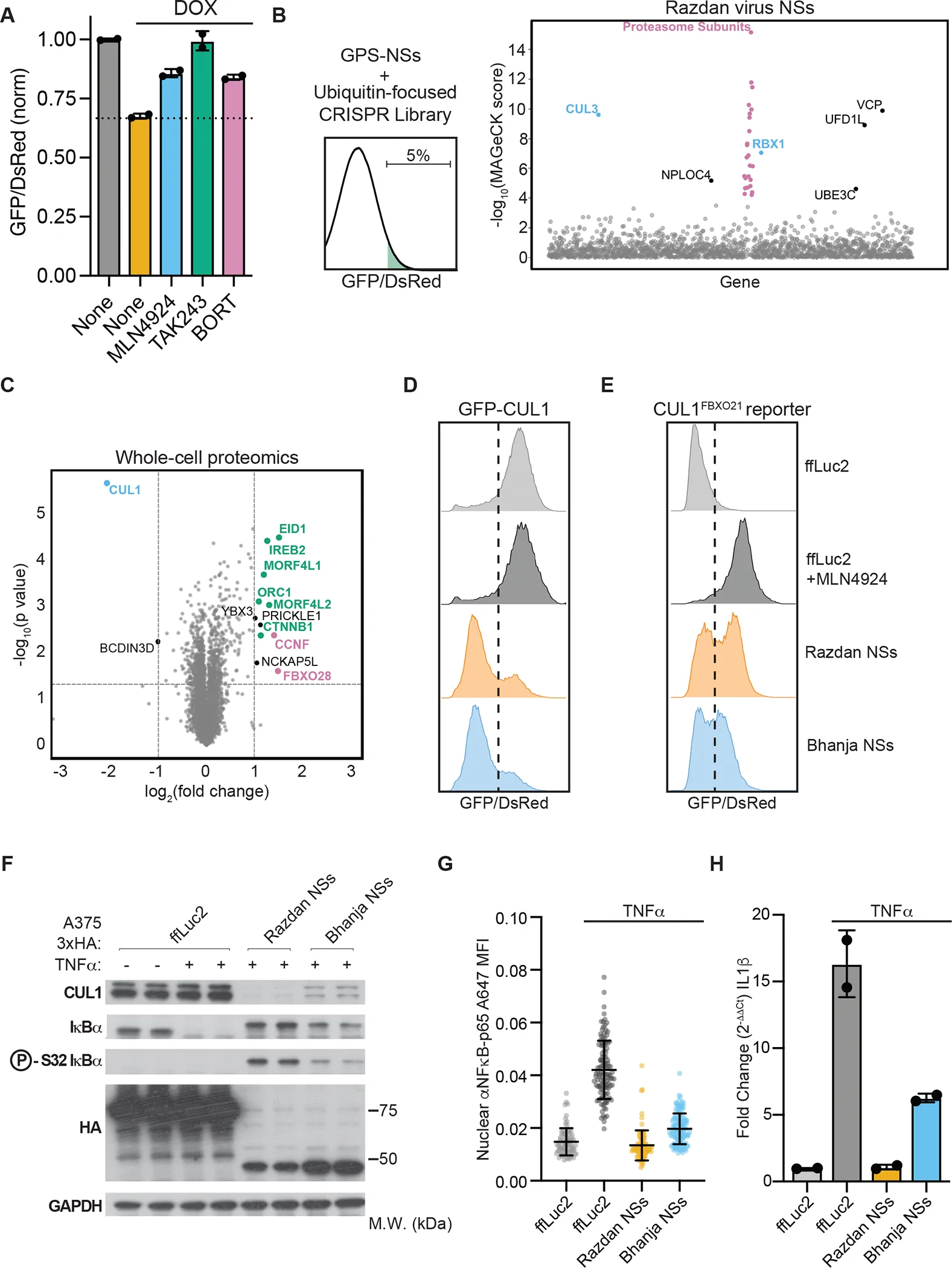
Razdan virus NSs engages CUL3 to degrade CUL1 and inhibit NFκB signaling. (A) Razdan NSs-mediated degradation requires Cullin-RING ligase activity. GPS reporter cells harboring TRE-αGFPnb-NSs (ID: YP_008719919.1.1) were treated with 200 ng/mL DOX for 4 h prior to addition of 1 μM inhibitor for the final 8 h (BORT: bortezomib). Fluorescence was measured in duplicate by flow cytometry and displayed as mean ± SD. (B) Razdan NSs degradation depends on CUL3. Ubiquitin-focused CRISPR screen in GPS-NSs (DsRed IRES GFP-YP_008719919.1.1) cells. See also Data S2G-H. (C) Razdan NSs expression reduces CUL1 abundance while increasing CUL1 substrates and adaptors. 293T cells harboring TRE-NSs or ffLuc2 control were treated with DOX for 16 h prior to harvesting lysates for whole-cell proteomics. Results show a single experiment performed in triplicate. See also Data S4B. (D-E) NSs genes from Razdan and Bhanja virus (ID: YP_009141016.1.1) degrade CUL1 and inhibit CUL1 activity. GPS-CUL1 (DsRed IRES GFP-CUL1, D) or CUL1^FBXO21^ reporter (DsRed IRES GFP-IFNA8_93–120, E) cells were analyzed as described in A. (F) Razdan and Bhanja virus NSs expression attenuate IκBα degradation following TNFα treatment. A375 cells harboring TRE3xHA-NSs were induced with DOX for 16 h followed by 100 ng/mL TNFα for 30 min. (G) Razdan and Bhanja virus NSs expression blocks nuclear translocation of NFκB p65 upon TNFα treatment. Cells from F were fixed, permeabilized, and stained with αNFκB-p65 antibody followed by AF647-labeled secondary antibody and DAPI to identify nuclei. Nuclear localization of p65 was quantified using CellProfiler (see also fig. S5F). (H) Razdan and Bhanja virus NSs expression attenuates induction of IL1β following TNFα stimulation. Cells from F were TNFα stimulated in duplicate for 4 h prior to qPCR for IL1B and ACTB endogenous control. See also fig. S5. Experimental results are representative of two or more independent experiments unless otherwise indicated.

**Fig. 6.**
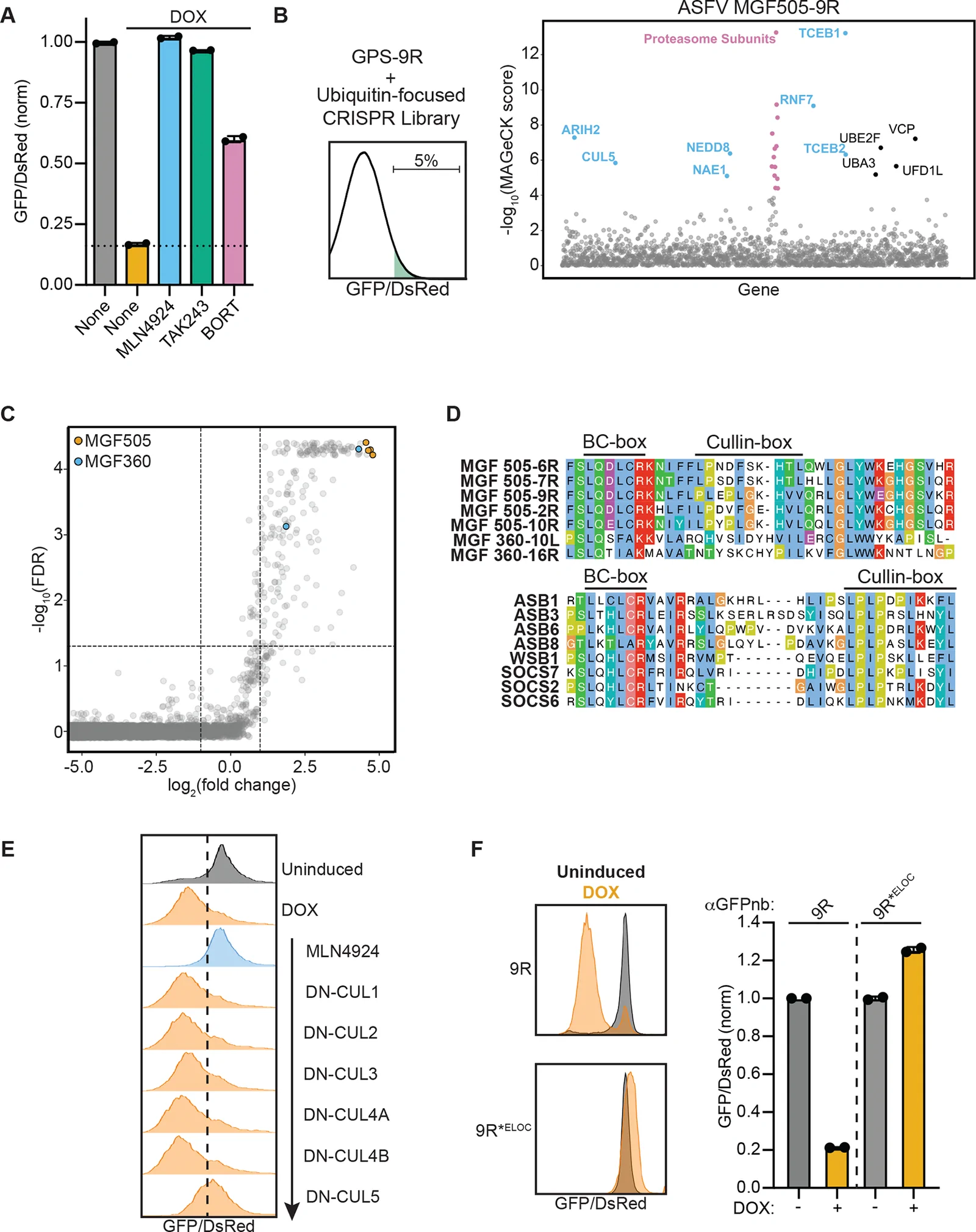
African Swine Fever Virus MGF505 genes are CUL5^ELOB/C^ ubiquitin ligases. (A) MGF505–9R degradation requires Cullin-RING ligase activity. GPS reporter cells harboring TRE-αGFPnb-MGF505–9R (ID: P0C9U9.0.1) were treated with 200 ng/mL doxycycline (DOX) for 4 h prior to addition of 1 μM inhibitor for the final 8 h (BORT: bortezomib). Fluorescence was measured in duplicate by flow cytometry and shown as mean ± SD. (B) MGF505–9R degradation depends on CUL5. Ubiquitin-focused CRISPR screen in GPS-MGF505–9R (DsRed IRES GFPP0C9U9.0.1) cells. See also Data S2I-J. (C) αGFPnb screen results showing ASFV MGF505 (orange) and MGF360 (blue) hits. Fold change and false discovery rate (FDR) were calculated by comparing barcode abundance in sorted cells relative to unsorted cells using MAGeCK. For plotting, jitter was introduced to distinguish closely spaced data points. See also, Fig. 1F. (D) MGF505 and MGF360 family genes contain conserved N-terminal BC- and Cullin-box motifs. Alignment was performed using Clustal Omega and visualized using Jalview. (E) MGF505–9R degradation is blocked by dominant-negative (DN) CUL5. GPS reporter cells harboring TREαGFPnb-MGF505–9R were transfected with DN-CUL constructs containing a tagBFP marker. Five hours post-transfection, cells were treated with DOX ± MLN4924 for 16 h prior to analysis of GFP/DsRed ratio in the tagBFP+ population by flow cytometry. (F) Conserved BC-box residues are required for MGF505–9R-mediated degradation. GPS reporter cells with TRE-αGFPnbMGF505–9R wild-type or mutant (*ELOC:L4A/L7A/C8A) were treated with DOX for 16 h prior to analysis as described in A. Experimental results are representative of two or more independent experiments unless otherwise indicated.

**Fig. 7.**
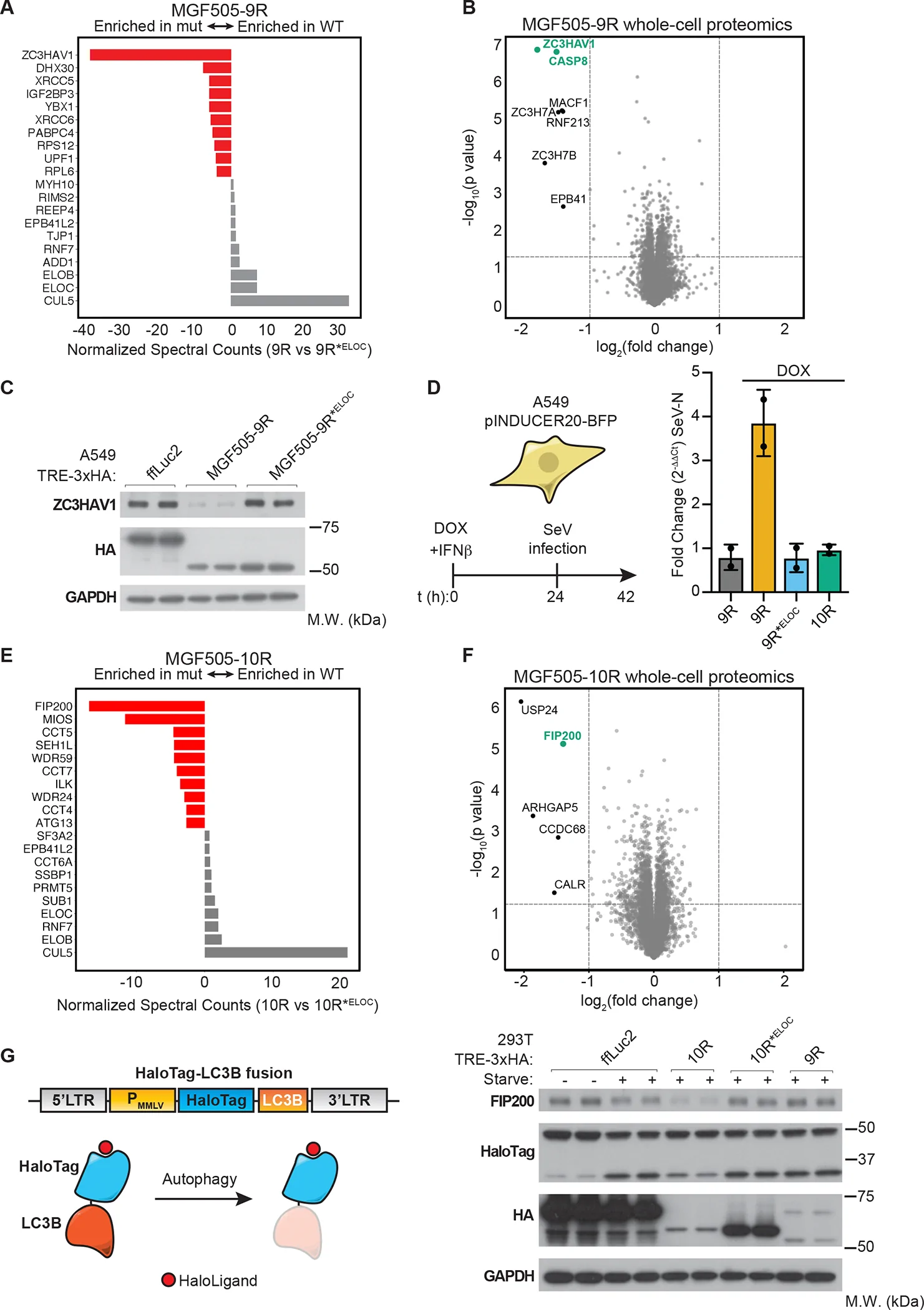
MGF505 family members 9R and 10R use CUL5^ELOB/C^ to regulate distinct substrates. (A) MGF505–9R differential immunoprecipitation mass-spectrometry (IP-MS). 293T cells harboring TRE-3xHA-MGF505–9R (ID: P0C9U9.0.1) wild-type or mutant (*ELOC:L4A/L7A/C8A) were induced with 200 ng/mL DOX prior to HA-immunoprecipitation and mass spectrometry. After filtering for common contaminants, spectral counts from individual pull-downs were normalized to bait and subtracted to obtain relative binding measurements. See also fig. S7A, Data S3E and S3G. (B) Whole-cell proteomics of MGF505–9R in porcine WSL-R cells. Plot shows abundance in MGF505–9R expressing cells relative to luciferase control. Proteins annotated as uncharacterized were omitted from the plot. Results show a single experiment performed in triplicate. See also fig. S7B and Data S4C. (C) MGF505–9R degradation of ZC3HAV1 depends on CUL5^ELOB/C^ binding. (D) MGF505–9R expression enhances Sendai virus (SeV) infection. A549 cells were treated with DOX and 5 nM IFNβ for 24 h prior to infection in duplicate with Sendai virus (1:500 dilution) for 18 h and qPCR for SeV-N relative to ACTB endogenous control. (E) MGF505–10R (ID: P0C9V2.0.1) differential IP-MS. 293T cells were transduced with TRE-3xHA-MGF505–10R wild-type or mutant (*ELOC: L4A/L7A/C8A) and analyzed as in A. See also fig. S8A and Data S3F-G. (F) Whole-cell proteomics of MGF505–10R in porcine WSL-R cells. Plot shows abundance in MGF505–10R expressing cells relative to luciferase control analyzed as described in B. See also Data S4D. (G) MGF505–10R attenuates autophagy upon amino acid starvation. 293T HaloTag-LC3 cells were induced with DOX and pulsed with 100 nM tetramethylrhodamine (TMR)-HaloLigand for 20 min, washed twice, and cultured in amino acid-free DMEM for 6 h prior to harvesting lysates for western blot (upper band: HaloTag-LC3 fusion, lower band: free HaloTag). See also fig. S8D. Experimental results are representative of two or more independent experiments unless otherwise indicated.

## Data Availability

Requests for resources and reagents should be directed to and will be fulfilled by the Lead Contact, Stephen J. Elledge (selledge@genetics.med.harvard.edu). Destination vectors used in this study have been deposited to Addgene. Other materials available upon request. The structure data have been deposited on RCSB and EMDB under PDB ID 9Q3E and EMD-72190. Raw whole-cell proteomics and IP-MS data have been deposited on PRIDE PXD078348. Raw sequencing data and analysis scripts along with AlphaFold 3 input files are available on Dryad (122).

## References

[1] Sadler AJ, Williams BR, Structure and function of the protein kinase R. Curr Top Microbiol Immunol 316, 253–292 (2007), doi: 10.1007/978-3-540-71329-6_13. 17969452

[2] Katze MG, He Y, Gale M Jr., Viruses and interferon: a fight for supremacy. Nat Rev Immunol 2, 675–687 (2002), doi: 10.1038/nri888. 12209136

[3] Newton K, Strasser A, Kayagaki N, Dixit VM, Cell death. Cell 187, 235–256 (2024), doi: 10.1016/j.cell.2023.11.044. 38242081

[4] Varshavsky A, The Ubiquitin System, Autophagy, and Regulated Protein Degradation. Annu Rev Biochem 86, 123–128 (2017), doi: 10.1146/annurev-biochem-061516-044859. 28654326

[5] Randow F, Lehner PJ, Viral avoidance and exploitation of the ubiquitin system. Nat Cell Biol 11, 527–534 (2009), doi: 10.1038/ncb0509-527. 19404332

[6] Isaacson MK, Ploegh HL, Ubiquitination, ubiquitin-like modifiers, and deubiquitination in viral infection. Cell Host Microbe 5, 559–570 (2009), doi: 10.1016/j.chom.2009.05.012. PMC7103382 19527883

[7] Heaton SM, Borg NA, Dixit VM, Ubiquitin in the activation and attenuation of innate antiviral immunity. J Exp Med 213, 1–13 (2016), doi: 10.1084/jem.20151531. PMC4710203 26712804

[8] Scheffner M, Werness BA, Huibregtse JM, Levine AJ, Howley PM, The E6 oncoprotein encoded by human papillomavirus types 16 and 18 promotes the degradation of p53. Cell 63, 1129–1136 (1990), doi: 10.1016/0092-8674(90)90409-8. 2175676

[9] Li YL , The structural basis for HIV-1 Vif antagonism of human APOBEC3G. Nature 615, 728–733 (2023), doi: 10.1038/s41586-023-05779-1. PMC10033410 36754086

[10] Sheehy AM, Gaddis NC, Choi JD, Malim MH, Isolation of a human gene that inhibits HIV-1 infection and is suppressed by the viral Vif protein. Nature 418, 646–650 (2002), doi: 10.1038/nature00939. 12167863

[11] Ishido S, Wang C, Lee BS, Cohen GB, Jung JU, Downregulation of major histocompatibility complex class I molecules by Kaposi's sarcoma-associated herpesvirus K3 and K5 proteins. J Virol 74, 5300–5309 (2000), doi: 10.1128/jvi.74.11.5300-5309.2000. PMC110885 10799607

[12] Coscoy L, Ganem D, Kaposi's sarcoma-associated herpesvirus encodes two proteins that block cell surface display of MHC class I chains by enhancing their endocytosis. Proc Natl Acad Sci U S A 97, 8051–8056 (2000), doi: 10.1073/pnas.140129797. PMC16668 10859362

[13] Stevenson PG, Efstathiou S, Doherty PC, Lehner PJ, Inhibition of MHC class I-restricted antigen presentation by gamma 2-herpesviruses. Proc Natl Acad Sci U S A 97, 8455–8460 (2000), doi: 10.1073/pnas.150240097. PMC26969 10890918

[14] Yu X , Induction of APOBEC3G ubiquitination and degradation by an HIV-1 Vif-Cul5-SCF complex. Science 302, 1056–1060 (2003), doi: 10.1126/science.1089591. 14564014

[15] Goebel SJ , The complete DNA sequence of vaccinia virus. Virology 179, 247–266, 517–263 (1990), doi: 10.1016/0042-6822(90)90294-2. 2219722

[16] Beard PM, Froggatt GC, Smith GL, Vaccinia virus kelch protein A55 is a 64 kDa intracellular factor that affects virus-induced cytopathic effect and the outcome of infection in a murine intradermal model. J Gen Virol 87, 1521–1529 (2006), doi: 10.1099/vir.0.81854-0. 16690916

[17] Fujimura E , A viral ORFeome library for systems-level genetic dissection of host-pathogen interactions. Cell 189, (2026), doi: 10.1016/j.cell.2026.05.024. PMC13429030 42392077

[18] Simpson LM , An affinity-directed phosphatase, AdPhosphatase, system for targeted protein dephosphorylation. Cell Chem Biol 30, 188–202 e186 (2023), doi: 10.1016/j.chembiol.2023.01.003. 36720221

[19] Ramirez DH , Engineering a Proximity-Directed O-GlcNAc Transferase for Selective Protein O-GlcNAcylation in Cells. ACS Chem Biol 15, 1059–1066 (2020), doi: 10.1021/acschembio.0c00074. PMC7296736 32119511

[20] Caussinus E, Kanca O, Affolter M, Fluorescent fusion protein knockout mediated by anti-GFP nanobody. Nat Struct Mol Biol 19, 117–121 (2011), doi: 10.1038/nsmb.2180. 22157958

[21] Kubala MH, Kovtun O, Alexandrov K, Collins BM, Structural and thermodynamic analysis of the GFP:GFP-nanobody complex. Protein Sci 19, 2389–2401 (2010), doi: 10.1002/pro.519. PMC3009406 20945358

[22] Yen HC, Xu Q, Chou DM, Zhao Z, Elledge SJ, Global protein stability profiling in mammalian cells. Science 322, 918–923 (2008), doi: 10.1126/science.1160489. 18988847

[23] Barro M, Patton JT, Rotavirus nonstructural protein 1 subverts innate immune response by inducing degradation of IFN regulatory factor 3. Proc Natl Acad Sci U S A 102, 4114–4119 (2005), doi: 10.1073/pnas.0408376102. PMC554789 15741273

[24] Poirson J , Proteome-scale discovery of protein degradation and stabilization effectors. Nature 628, 878–886 (2024), doi: 10.1038/s41586-024-07224-3. 38509365

[25] Hrecka K , Lentiviral Vpr usurps Cul4-DDB1[VprBP] E3 ubiquitin ligase to modulate cell cycle. Proc Natl Acad Sci U S A 104, 11778–11783 (2007), doi: 10.1073/pnas.0702102104. PMC1906728 17609381

[26] Lutz LM, Pace CR, Arnold MM, Rotavirus NSP1 Associates with Components of the Cullin RING Ligase Family of E3 Ubiquitin Ligases. J Virol 90, 6036–6048 (2016), doi: 10.1128/JVI.00704-16. PMC4907217 27099313

[27] Boggio R, Passafaro A, Chiocca S, Targeting SUMO E1 to ubiquitin ligases: a viral strategy to counteract sumoylation. J Biol Chem 282, 15376–15382 (2007), doi: 10.1074/jbc.M700889200. 17392274

[28] Gilson T, Cheng CY, Hur WS, Blanchette P, Branton PE, Analysis of the Cullin binding sites of the E4orf6 proteins of human adenovirus E3 ubiquitin ligases. J Virol 88, 3885–3897 (2014), doi: 10.1128/JVI.03579-13. PMC3993529 24453364

[29] Querido E , Degradation of p53 by adenovirus E4orf6 and E1B55K proteins occurs via a novel mechanism involving a Cullin-containing complex. Genes Dev 15, 3104–3117 (2001), doi: 10.1101/gad.926401. PMC312842 11731475

[30] Riera E, Garcia-Belmonte R, Madrid R, Perez-Nunez D, Revilla Y, African swine fever virus ubiquitin-conjugating enzyme pI215L inhibits IFN-I signaling pathway through STAT2 degradation. Front Microbiol 13, 1081035 (2022), doi: 10.3389/fmicb.2022.1081035. PMC9880986 36713190

[31] Giaquinto C , Summary of effectiveness and impact of rotavirus vaccination with the oral pentavalent rotavirus vaccine: a systematic review of the experience in industrialized countries. Hum Vaccin 7, 734–748 (2011), doi: 10.4161/hv.7.7.15511. 21734466

[32] Hallowell BD, Tate J, Parashar U, An overview of rotavirus vaccination programs in developing countries. Expert Rev Vaccines 19, 529–537 (2020), doi: 10.1080/14760584.2020.1775079. PMC9234970 32543239

[33] Ding S , Comparative Proteomics Reveals Strain-Specific beta-TrCP Degradation via Rotavirus NSP1 Hijacking a Host Cullin-3-Rbx1 Complex. PLoS Pathog 12, e1005929 (2016), doi: 10.1371/journal.ppat.1005929. PMC5051689 27706223

[34] Arnold MM, The Rotavirus Interferon Antagonist NSP1: Many Targets, Many Questions. J Virol 90, 5212–5215 (2016), doi: 10.1128/JVI.03068-15. PMC4934742 27009959

[35] Hou G , SAMD9 senses cytosolic double-stranded nucleic acids in epithelial and mesenchymal cells to induce antiviral immunity. Nat Commun 16, 3756 (2025), doi: 10.1038/s41467-025-59090-w. PMC12015307 40263291

[36] Pintard L, Willems A, Peter M, Cullin-based ubiquitin ligases: Cul3-BTB complexes join the family. EMBO J 23, 1681–1687 (2004), doi: 10.1038/sj.emboj.7600186. PMC394240 15071497

[37] Nomburg J , Birth of protein folds and functions in the virome. Nature 633, 710–717 (2024), doi: 10.1038/s41586-024-07809-y. PMC11410667 39187718

[38] Bai C , SKP1 connects cell cycle regulators to the ubiquitin proteolysis machinery through a novel motif, the F-box. Cell 86, 263–274 (1996), doi: 10.1016/s0092-8674(00)80098-7. 8706131

[39] Skowyra D, Craig KL, Tyers M, Elledge SJ, Harper JW, F-box proteins are receptors that recruit phosphorylated substrates to the SCF ubiquitin-ligase complex. Cell 91, 209–219 (1997), doi: 10.1016/s0092-8674(00)80403-1. 9346238

[40] Nguyen HC, Yang H, Fribourgh JL, Wolfe LS, Xiong Y, Insights into Cullin-RING E3 ubiquitin ligase recruitment: structure of the VHL-EloBC-Cul2 complex. Structure 23, 441–449 (2015), doi: 10.1016/j.str.2014.12.014. PMC4351159 25661653

[41] Dai Z , Structural insights into the ubiquitylation strategy of the oligomeric CRL2(FEM1B) E3 ubiquitin ligase. EMBO J 43, 1089–1109 (2024), doi: 10.1038/s44318-024-00047-y. PMC10943247 38360992

[42] Kim YK , Structural basis of intersubunit recognition in elongin BC-cullin 5-SOCS box ubiquitin-protein ligase complexes. Acta Crystallogr D Biol Crystallogr 69, 1587–1597 (2013), doi: 10.1107/S0907444913011220. 23897481

[43] Guo Y , Structural basis for hijacking CBF-beta and CUL5 E3 ligase complex by HIV-1 Vif. Nature 505, 229–233 (2014), doi: 10.1038/nature12884. 24402281

[44] Cardote TAF, Gadd MS, Ciulli A, Crystal Structure of the Cul2-Rbx1-EloBC-VHL Ubiquitin Ligase Complex. Structure 25, 901–911 e903 (2017), doi: 10.1016/j.str.2017.04.009. PMC5462531 28591624

[45] Shi CY , The ZSWIM8 ubiquitin ligase mediates target-directed microRNA degradation. Science 370, (2020), doi: 10.1126/science.abc9359. PMC8356967 33184237

[46] Han J , A ubiquitin ligase mediates target-directed microRNA decay independently of tailing and trimming. Science 370, (2020), doi: 10.1126/science.abc9546. PMC8177725 33184234

[47] Farnung J , The E3 ubiquitin ligase mechanism specifying targeted microRNA degradation. Nature 652, 784–793 (2026), doi: 10.1038/s41586-026-10232-0. PMC13083262 41851464

[48] Zhao B , Structural basis for concerted recruitment and activation of IRF-3 by innate immune adaptor proteins. Proc Natl Acad Sci U S A 113, E3403–3412 (2016), doi: 10.1073/pnas.1603269113. PMC4914169 27302953

[49] Graff JW, Ettayebi K, Hardy ME, Rotavirus NSP1 inhibits NFkappaB activation by inducing proteasome-dependent degradation of beta-TrCP: a novel mechanism of IFN antagonism. PLoS Pathog 5, e1000280 (2009), doi: 10.1371/journal.ppat.1000280. PMC2627925 19180189

[50] Winston JT , The SCFbeta-TRCP-ubiquitin ligase complex associates specifically with phosphorylated destruction motifs in IkappaBalpha and beta-catenin and stimulates IkappaBalpha ubiquitination in vitro. Genes Dev 13, 270–283 (1999), doi: 10.1101/gad.13.3.270. PMC316433 9990852

[51] Yaron A , Identification of the receptor component of the IkappaBalpha-ubiquitin ligase. Nature 396, 590–594 (1998), doi: 10.1038/25159. 9859996

[52] Kanai Y , Entirely plasmid-based reverse genetics system for rotaviruses. Proc Natl Acad Sci U S A 114, 2349–2354 (2017), doi: 10.1073/pnas.1618424114. PMC5338561 28137864

[53] Johnson RI , Characterization of Teviot virus, an Australian bat-borne paramyxovirus. J Gen Virol 100, 403–413 (2019), doi: 10.1099/jgv.0.001214. 30688635

[54] Bharaj P , The Matrix Protein of Nipah Virus Targets the E3-Ubiquitin Ligase TRIM6 to Inhibit the IKKepsilon Kinase-Mediated Type-I IFN Antiviral Response. PLoS Pathog 12, e1005880 (2016), doi: 10.1371/journal.ppat.1005880. PMC5021333 27622505

[55] Stogios PJ, Prive GG, The BACK domain in BTB-kelch proteins. Trends Biochem Sci 29, 634–637 (2004), doi: 10.1016/j.tibs.2004.10.003. 15544948

[56] Philips RL , The JAK-STAT pathway at 30: Much learned, much more to do. Cell 185, 3857–3876 (2022), doi: 10.1016/j.cell.2022.09.023. PMC9815833 36240739

[57] Alkan C , Isolation, genetic characterization, and seroprevalence of Adana virus, a novel phlebovirus belonging to the Salehabad virus complex, in Turkey. J Virol 89, 4080–4091 (2015), doi: 10.1128/JVI.03027-14. PMC4442372 25653443

[58] Wu G , Structure of a beta-TrCP1-Skp1-beta-catenin complex: destruction motif binding and lysine specificity of the SCF(beta-TrCP1) ubiquitin ligase. Mol Cell 11, 1445–1456 (2003), doi: 10.1016/s1097-2765(03)00234-x. 12820959

[59] Glassman CR , Structure of a Janus kinase cytokine receptor complex reveals the basis for dimeric activation. Science 376, 163–169 (2022), doi: 10.1126/science.abn8933. PMC9306331 35271300

[60] Li H , Rift Valley fever virus coordinates the assembly of a programmable E3 ligase to promote viral replication. Cell 187, 6896–6913 e6815 (2024), doi: 10.1016/j.cell.2024.09.008. 39366381

[61] Mudhasani R , Protein Kinase R Degradation Is Essential for Rift Valley Fever Virus Infection and Is Regulated by SKP1-CUL1-F-box (SCF)FBXW11-NSs E3 Ligase. PLoS Pathog 12, e1005437 (2016), doi: 10.1371/journal.ppat.1005437. PMC4737497 26837067

[62] Kainulainen M, Lau S, Samuel CE, Hornung V, Weber F, NSs Virulence Factor of Rift Valley Fever Virus Engages the F-Box Proteins FBXW11 and beta-TRCP1 To Degrade the Antiviral Protein Kinase PKR. J Virol 90, 6140–6147 (2016), doi: 10.1128/JVI.00016-16. PMC4907219 27122577

[63] Lvov DK , Razdan virus, a new ungrouped bunyavirus isolated from Dermacentor marginatus ticks in Armenia. Acta Virol 22, 506–508 (1978), doi:35950

[64] Hubalek Z, Halouzka J, Numerical comparative serology of the Bhanja antigenic group (Bunyaviridae). Arch Virol 84, 175–180 (1985), doi: 10.1007/BF01378970. 2581527

[65] Shah KV, Work TH, Bhanja virus: a new arbovirus from ticks Haemaphysalis intermedia Warburton and Nuttall, 1909, in Orissa, India. Indian J Med Res 57, 793–798 (1969), doi:5820426

[66] Calisher CH, Goodpasture HC, Human infection with Bhanja virus. Am J Trop Med Hyg 24, 1040–1042 (1975), doi: 10.4269/ajtmh.1975.24.1040. 812372

[67] Zhang Z , Elucidation of E3 ubiquitin ligase specificity through proteome-wide internal degron mapping. Mol Cell 83, 3377–3392 e3376 (2023), doi: 10.1016/j.molcel.2023.08.022. PMC10594193 37738965

[68] Mansur DS , Poxvirus targeting of E3 ligase beta-TrCP by molecular mimicry: a mechanism to inhibit NF-kappaB activation and promote immune evasion and virulence. PLoS Pathog 9, e1003183 (2013), doi: 10.1371/journal.ppat.1003183. PMC3585151 23468625

[69] You S , African swine fever outbreaks in China led to gross domestic product and economic losses. Nat Food 2, 802–808 (2021), doi: 10.1038/s43016-021-00362-1. 37117973

[70] Dixon LK, Sun H, Roberts H, African swine fever. Antiviral Res 165, 34–41 (2019), doi: 10.1016/j.antiviral.2019.02.018. 30836106

[71] Gao G, Guo X, Goff SP, Inhibition of retroviral RNA production by ZAP, a CCCH-type zinc finger protein. Science 297, 1703–1706 (2002), doi: 10.1126/science.1074276. 12215647

[72] Ficarelli M, Neil SJD, Swanson CM, Targeted Restriction of Viral Gene Expression and Replication by the ZAP Antiviral System. Annu Rev Virol 8, 265–283 (2021), doi: 10.1146/annurev-virology-091919-104213. 34129371

[73] Ye P, Liu S, Zhu Y, Chen G, Gao G, DEXH-Box protein DHX30 is required for optimal function of the zinc-finger antiviral protein. Protein Cell 1, 956–964 (2010), doi: 10.1007/s13238-010-0117-8. PMC4875121 21204022

[74] Peng C , Zinc-finger antiviral protein (ZAP) is a restriction factor for replication of modified vaccinia virus Ankara (MVA) in human cells. PLoS Pathog 16, e1008845 (2020), doi: 10.1371/journal.ppat.1008845. PMC7485971 32866210

[75] Gonzalez-Perez AC , The Zinc Finger Antiviral Protein ZAP Restricts Human Cytomegalovirus and Selectively Binds and Destabilizes Viral UL4/UL5 Transcripts. mBio 12, (2021), doi: 10.1128/mBio.02683-20. PMC8263000 33947766

[76] Treiber T , A Compendium of RNA-Binding Proteins that Regulate MicroRNA Biogenesis. Mol Cell 66, 270–284 e213 (2017), doi: 10.1016/j.molcel.2017.03.014. 28431233

[77] Varfolomeev EE , Targeted disruption of the mouse Caspase 8 gene ablates cell death induction by the TNF receptors, Fas/Apo1, and DR3 and is lethal prenatally. Immunity 9, 267–276 (1998), doi: 10.1016/s1074-7613(00)80609-3. 9729047

[78] Zhang B , Zinc Finger CCCH-Type Antiviral Protein 1 Restricts the Viral Replication by Positively Regulating Type I Interferon Response. Front Microbiol 11, 1912 (2020), doi: 10.3389/fmicb.2020.01912. PMC7456897 32922375

[79] Hosokawa N , Nutrient-dependent mTORC1 association with the ULK1-Atg13-FIP200 complex required for autophagy. Mol Biol Cell 20, 1981–1991 (2009), doi: 10.1091/mbc.e08-12-1248. PMC2663915 19211835

[80] Bar-Peled L , A Tumor suppressor complex with GAP activity for the Rag GTPases that signal amino acid sufficiency to mTORC1. Science 340, 1100–1106 (2013), doi: 10.1126/science.1232044. PMC3728654 23723238

[81] Yim WW, Yamamoto H, Mizushima N, A pulse-chasable reporter processing assay for mammalian autophagic flux with HaloTag. Elife 11, (2022), doi: 10.7554/eLife.78923. PMC9385206 35938926

[82] Morita K , Genome-wide CRISPR screen identifies TMEM41B as a gene required for autophagosome formation. J Cell Biol 217, 3817–3828 (2018), doi: 10.1083/jcb.201804132. PMC6219718 30093494

[83] Shimmon GL, Hui JYK, Wileman TE, Netherton CL, Autophagy impairment by African swine fever virus. J Gen Virol 102, (2021), doi: 10.1099/jgv.0.001637. PMC8513644 34406116

[84] Huang Z , African Swine Fever Virus MGF505–7R Interacts with Interferon Regulatory Factor 9 to Evade the Type I Interferon Signaling Pathway and Promote Viral Replication. J Virol 97, e0197722 (2023), doi: 10.1128/jvi.01977-22. PMC10062159 36815839

[85] Li D , African swine fever virus protein MGF-505–7R promotes virulence and pathogenesis by inhibiting JAK1- and JAK2-mediated signaling. J Biol Chem 297, 101190 (2021), doi: 10.1016/j.jbc.2021.101190. PMC8526981 34517008

[86] Sunwoo SY , Deletion of MGF505–2R Gene Activates the cGAS-STING Pathway Leading to Attenuation and Protection against Virulent African Swine Fever Virus. Vaccines (Basel) 12, (2024), doi: 10.3390/vaccines12040407. PMC11054455 38675789

[87] Yang K , African swine fever virus MGF505–11R inhibits type I interferon production by negatively regulating the cGAS-STING-mediated signaling pathway. Vet Microbiol 263, 109265 (2021), doi: 10.1016/j.vetmic.2021.109265. 34710767

[88] Yao M , African swine fever virus MGF505–6R attenuates type I interferon production by targeting STING for degradation. Front Immunol 15, 1380220 (2024), doi: 10.3389/fimmu.2024.1380220. PMC11116646 38799458

[89] Zhu G , Combinational Deletions of MGF110–9L and MGF505–7R Genes from the African Swine Fever Virus Inhibit TBK1 Degradation by an Autophagy Activator PIK3C2B To Promote Type I Interferon Production. J Virol 97, e0022823 (2023), doi: 10.1128/jvi.00228-23. PMC10231249 37162350

[90] Yang K , African swine fever virus MGF505–7R protein interacted with IRF7and TBK1 to inhibit type I interferon production. Virus Res 322, 198931 (2022), doi: 10.1016/j.virusres.2022.198931. 36130654

[91] Gao Q , The African Swine Fever Virus with MGF360 and MGF505 Deleted Reduces the Apoptosis of Porcine Alveolar Macrophages by Inhibiting the NF-kappaB Signaling Pathway and Interleukin-1beta. Vaccines (Basel) 9, (2021), doi: 10.3390/vaccines9111371. PMC8622997 34835302

[92] van Kempen M , Fast and accurate protein structure search with Foldseek. Nat Biotechnol 42, 243–246 (2024), doi: 10.1038/s41587-023-01773-0. PMC10869269 37156916

[93] Senkevich TG, Yutin N, Wolf YI, Koonin EV, Moss B, Ancient Gene Capture and Recent Gene Loss Shape the Evolution of Orthopoxvirus-Host Interaction Genes. mBio 12, e0149521 (2021), doi: 10.1128/mBio.01495-21. PMC8406176 34253028

[94] Hou G, Zeng Q, Matthijnssens J, Greenberg HB, Ding S, Rotavirus NSP1 Contributes to Intestinal Viral Replication, Pathogenesis, and Transmission. mBio 12, e0320821 (2021), doi: 10.1128/mBio.03208-21. PMC8669464 34903043

[95] Koren I , The Eukaryotic Proteome Is Shaped by E3 Ubiquitin Ligases Targeting C-Terminal Degrons. Cell 173, 1622–1635 e1614 (2018), doi: 10.1016/j.cell.2018.04.028. PMC6003881 29779948

[96] Meerbrey KL , The pINDUCER lentiviral toolkit for inducible RNA interference in vitro and in vivo. Proc Natl Acad Sci U S A 108, 3665–3670 (2011), doi: 10.1073/pnas.1019736108. PMC3048138 21307310

[97] Wall VE, Garvey LA, Mehalko JL, Procter LV, Esposito D, Combinatorial assembly of clone libraries using site-specific recombination. Methods Mol Biol 1116, 193–208 (2014), doi: 10.1007/978-1-62703-764-8_14. 24395366

[98] Gentili M , The N-Terminal Domain of cGAS Determines Preferential Association with Centromeric DNA and Innate Immune Activation in the Nucleus. Cell Rep 26, 2377–2393 e2313 (2019), doi: 10.1016/j.celrep.2019.01.105. PMC6391843 30811988

[99] Thomsen EA , Single-Cell Monitoring of Activated Innate Immune Signaling by a d2eGFP-Based Reporter Mimicking Time-Restricted Activation of IFNB1 Expression. Front Cell Infect Microbiol 11, 784762 (2021), doi: 10.3389/fcimb.2021.784762. PMC8803904 35118008

[100] Gotzke H , The ALFA-tag is a highly versatile tool for nanobody-based bioscience applications. Nat Commun 10, 4403 (2019), doi: 10.1038/s41467-019-12301-7. PMC6764986 31562305

[101] Gu X , SAMTOR is an S-adenosylmethionine sensor for the mTORC1 pathway. Science 358, 813–818 (2017), doi: 10.1126/science.aao3265. PMC5747364 29123071

[102] Sanjana NE, Shalem O, Zhang F, Improved vectors and genome-wide libraries for CRISPR screening. Nat Methods 11, 783–784 (2014), doi: 10.1038/nmeth.3047. PMC4486245 25075903

[103] Chen B , Dynamic imaging of genomic loci in living human cells by an optimized CRISPR/Cas system. Cell 155, 1479–1491 (2013), doi: 10.1016/j.cell.2013.12.001. PMC3918502 24360272

[104] Timms RT , Defining E3 ligase-substrate relationships through multiplex CRISPR screening. Nat Cell Biol 25, 1535–1545 (2023), doi: 10.1038/s41556-023-01229-2. PMC10567573 37735597

[105] Baek K , Unveiling the hidden interactome of CRBN molecular glues. Nat Commun 16, 6831 (2025), doi: 10.1038/s41467-025-62099-w. PMC12289921 40707481

[106] Meyer M, Kircher M, Illumina sequencing library preparation for highly multiplexed target capture and sequencing. Cold Spring Harb Protoc 2010, pdb prot5448 (2010), doi: 10.1101/pdb.prot5448. 20516186

[107] Li W , MAGeCK enables robust identification of essential genes from genome-scale CRISPR/Cas9 knockout screens. Genome Biol 15, 554 (2014), doi: 10.1186/s13059-014-0554-4. PMC4290824 25476604

[108] Almagro Armenteros JJ , SignalP 5.0 improves signal peptide predictions using deep neural networks. Nat Biotechnol 37, 420–423 (2019), doi: 10.1038/s41587-019-0036-z. 30778233

[109] Kong AT, Leprevost FV, Avtonomov DM, Mellacheruvu D, Nesvizhskii AI, MSFragger: ultrafast and comprehensive peptide identification in mass spectrometry-based proteomics. Nat Methods 14, 513–520 (2017), doi: 10.1038/nmeth.4256. PMC5409104 28394336

[110] Li E , Rhesus rotavirus NSP1 mediates extra-intestinal infection and is a contributing factor for biliary obstruction. PLoS Pathog 20, e1012609 (2024), doi: 10.1371/journal.ppat.1012609. PMC11476687 39348381

[111] Abramson J , Accurate structure prediction of biomolecular interactions with AlphaFold 3. Nature 630, 493–500 (2024), doi: 10.1038/s41586-024-07487-w. PMC11168924 38718835

[112] Pettersen EF , UCSF ChimeraX: Structure visualization for researchers, educators, and developers. Protein Sci 30, 70–82 (2021), doi: 10.1002/pro.3943. PMC7737788 32881101

[113] Altschul SF , Gapped BLAST and PSI-BLAST: a new generation of protein database search programs. Nucleic Acids Res 25, 3389–3402 (1997), doi: 10.1093/nar/25.17.3389. PMC146917 9254694

[114] Steinegger M, Soding J, MMseqs2 enables sensitive protein sequence searching for the analysis of massive data sets. Nat Biotechnol 35, 1026–1028 (2017), doi: 10.1038/nbt.3988. 29035372

[115] Sievers F , Fast, scalable generation of high-quality protein multiple sequence alignments using Clustal Omega. Mol Syst Biol 7, 539 (2011), doi: 10.1038/msb.2011.75. PMC3261699 21988835

[116] Madeira F , The EMBL-EBI Job Dispatcher sequence analysis tools framework in 2024. Nucleic Acids Res 52, W521–W525 (2024), doi: 10.1093/nar/gkae241. PMC11223882 38597606

[117] Waterhouse AM, Procter JB, Martin DM, Clamp M, Barton GJ, Jalview Version 2--a multiple sequence alignment editor and analysis workbench. Bioinformatics 25, 1189–1191 (2009), doi: 10.1093/bioinformatics/btp033. PMC2672624 19151095

[118] Letunic I, Bork P, Interactive Tree of Life (iTOL) v6: recent updates to the phylogenetic tree display and annotation tool. Nucleic Acids Res 52, W78–W82 (2024), doi: 10.1093/nar/gkae268. PMC11223838 38613393

[119] Navarrete-Perea J, Yu Q, Gygi SP, Paulo JA, Streamlined Tandem Mass Tag (SL-TMT) Protocol: An Efficient Strategy for Quantitative (Phospho)proteome Profiling Using Tandem Mass Tag-Synchronous Precursor Selection-MS3. J Proteome Res 17, 2226–2236 (2018), doi: 10.1021/acs.jproteome.8b00217. PMC5994137 29734811

[120] Schindelin J , Fiji: an open-source platform for biological-image analysis. Nat Methods 9, 676–682 (2012), doi: 10.1038/nmeth.2019. PMC3855844 22743772

[121] Stirling DR , CellProfiler 4: improvements in speed, utility and usability. BMC Bioinformatics 22, 433 (2021), doi: 10.1186/s12859-021-04344-9. PMC8431850 34507520

[122] Glassman CR , Data for virome-wide ubiquitin ligase discovery reveals diverse mechanisms of immune evasion, doi: 10.5061/dryad.pzgmsbd3d PMC13769053 42424437

