# Supplementary material for: Virome-wide ubiquitin ligase discovery reveals diverse mechanisms of immune evasion
Source: Science. Author manuscript; Available in PMC 2026 Nov 14. (PMC13769053; doi:10.1126/science.aec6299)

Unedited blots (LICOR) - Figure 2D

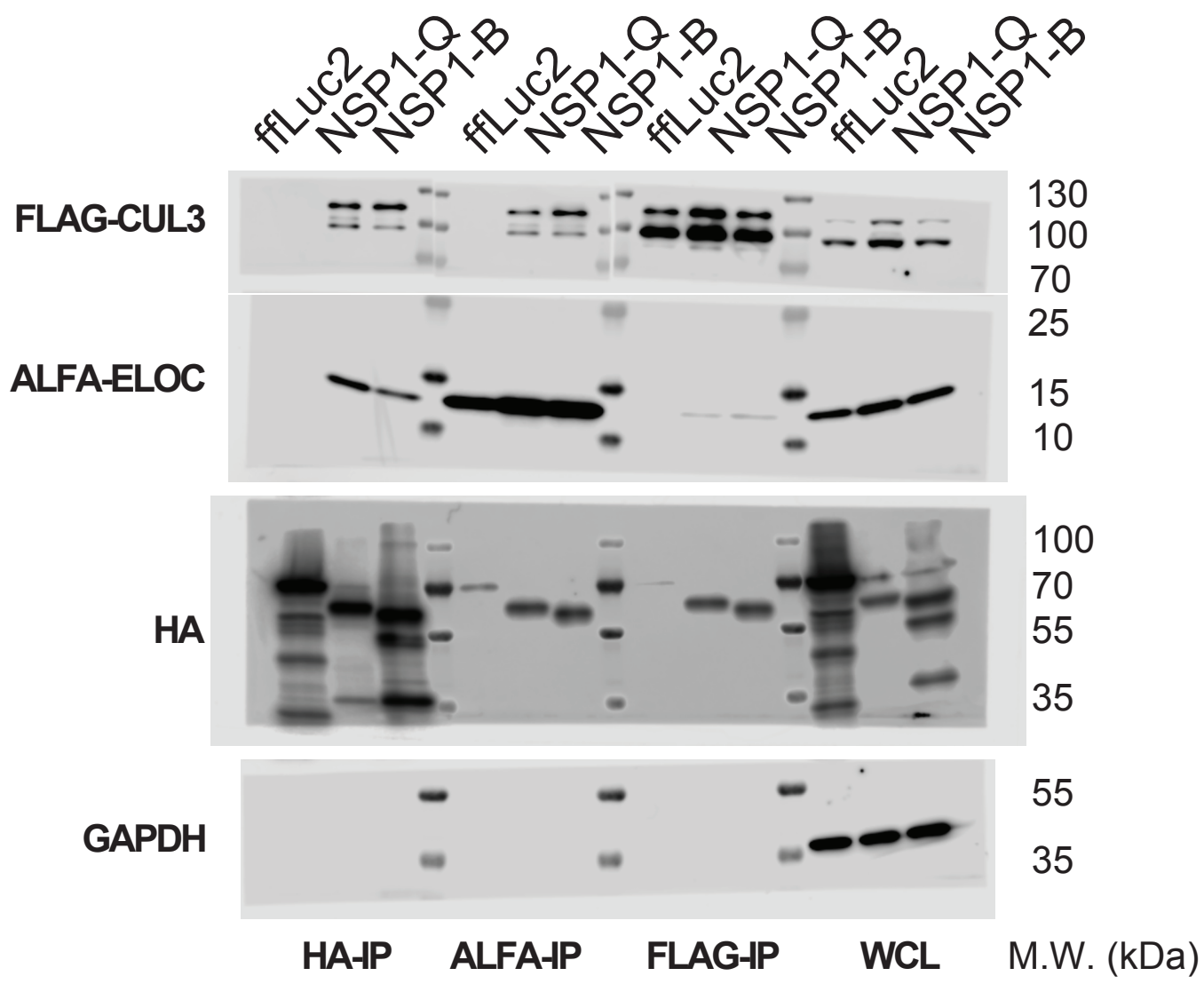

Unedited blots (ChemiDoc MP) - Figure S2G

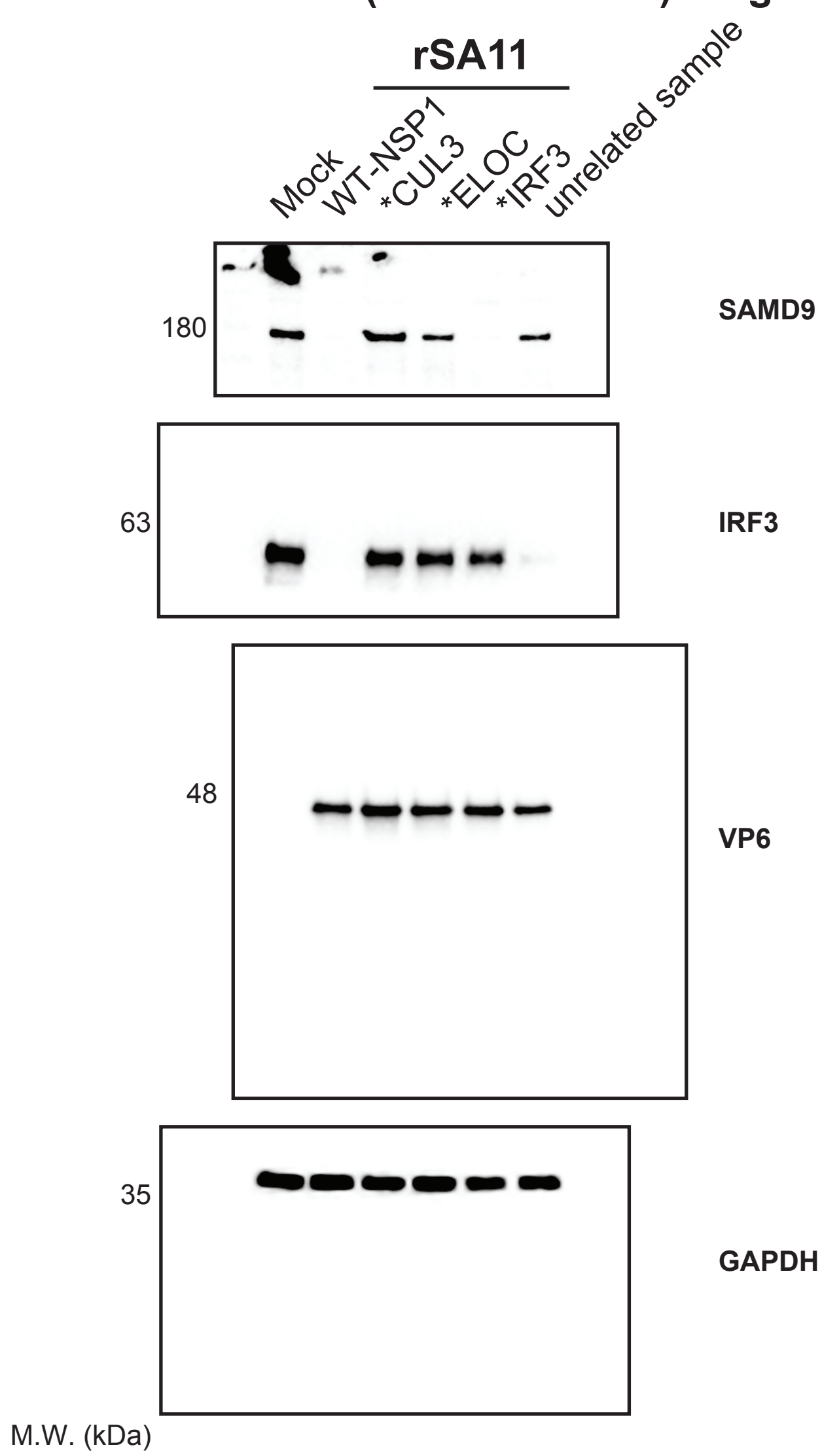

Unedited blots (film) - Figure S1B

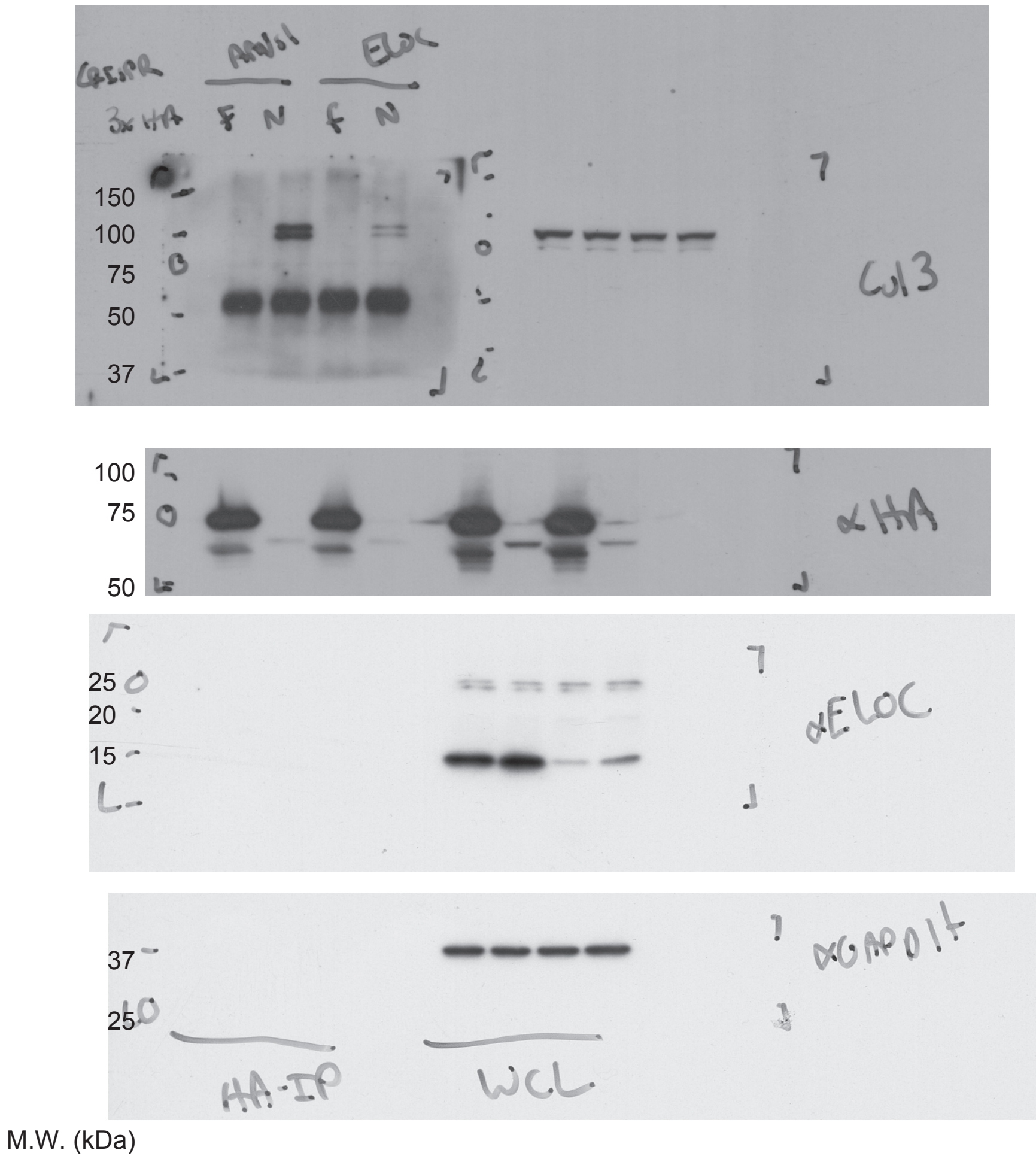

Unedited blots (LICOR) - Figure S1C

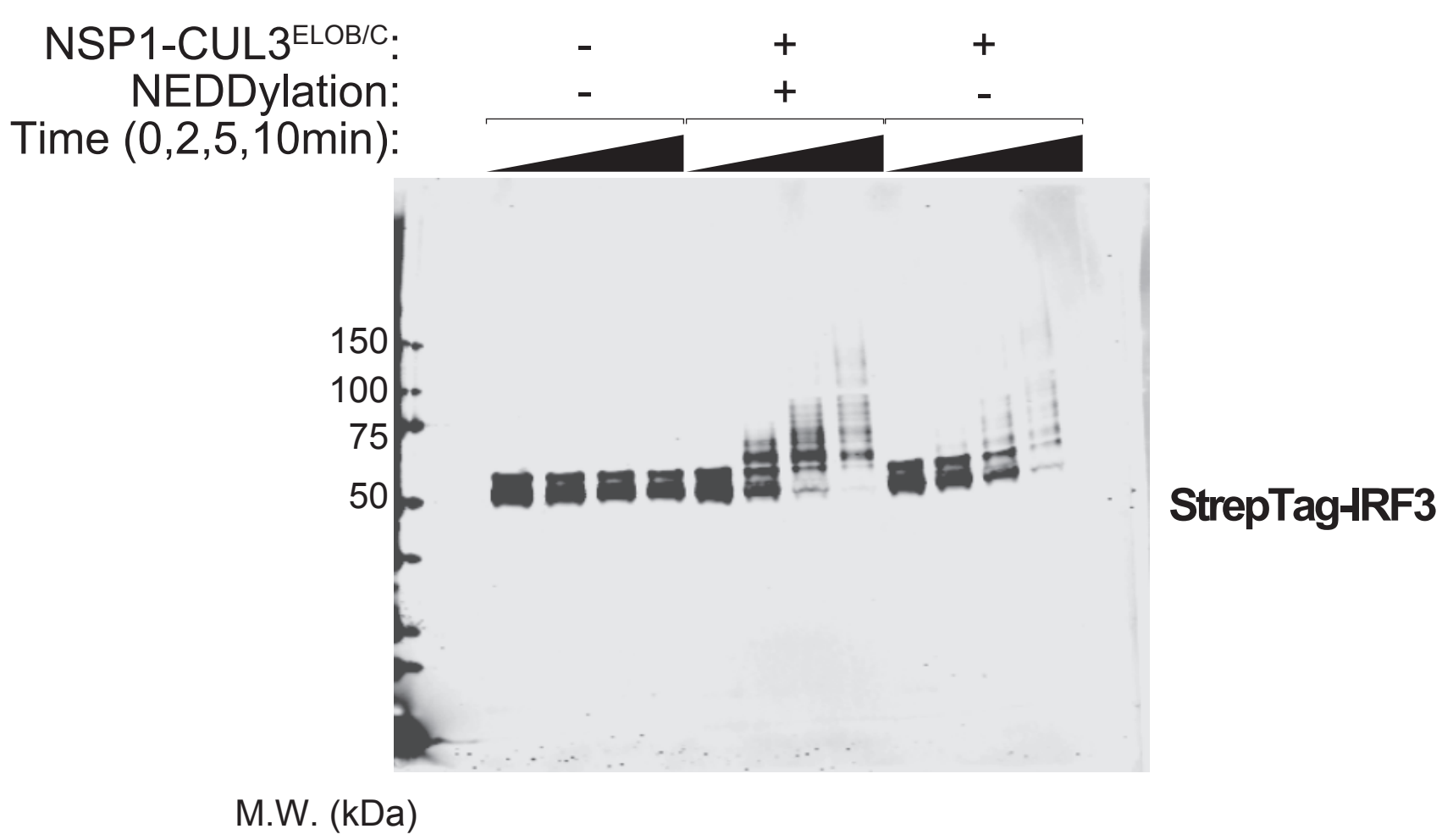

Unedited blots (film) - Figure S2C

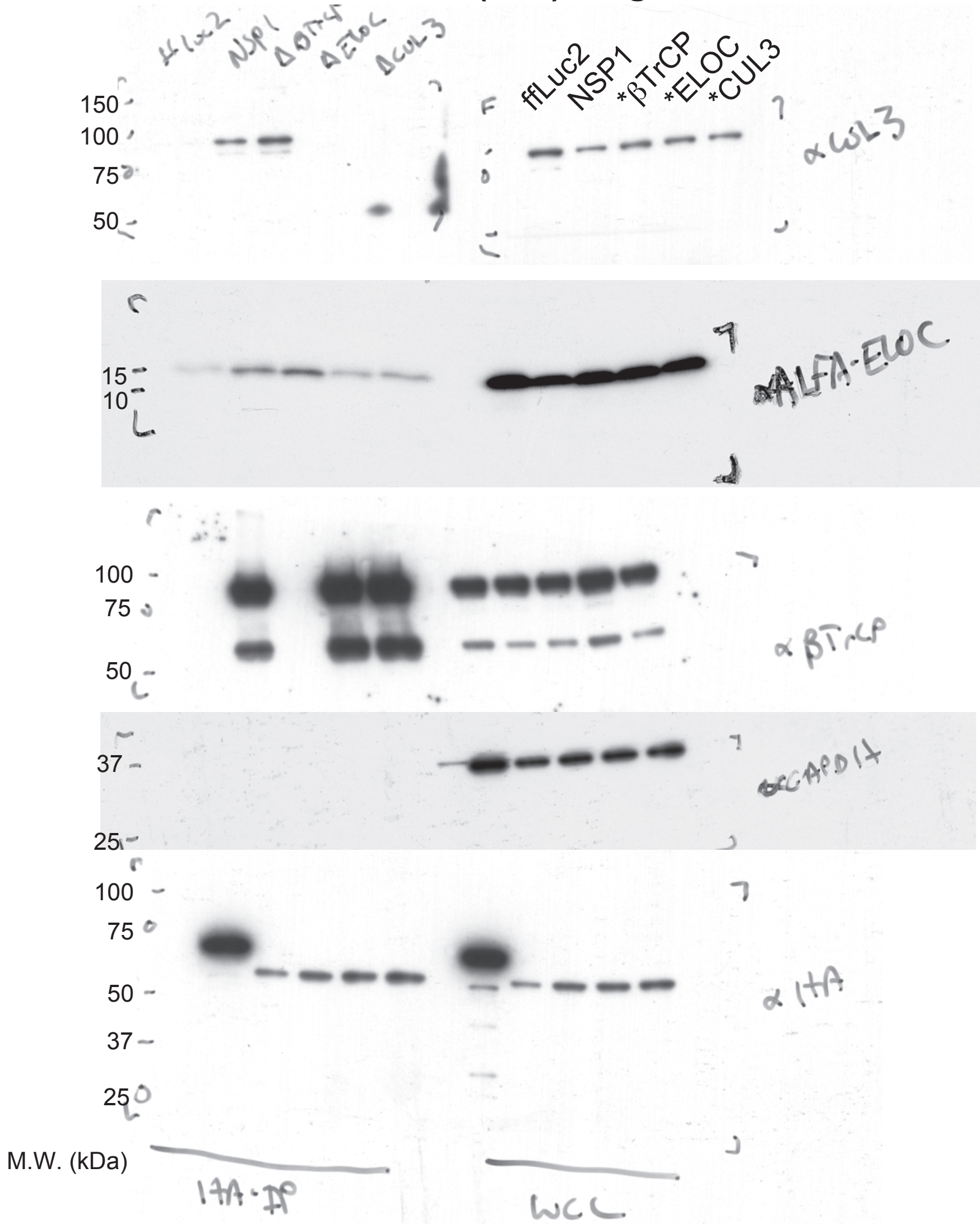

Unedited blots (film) - Figure S2D

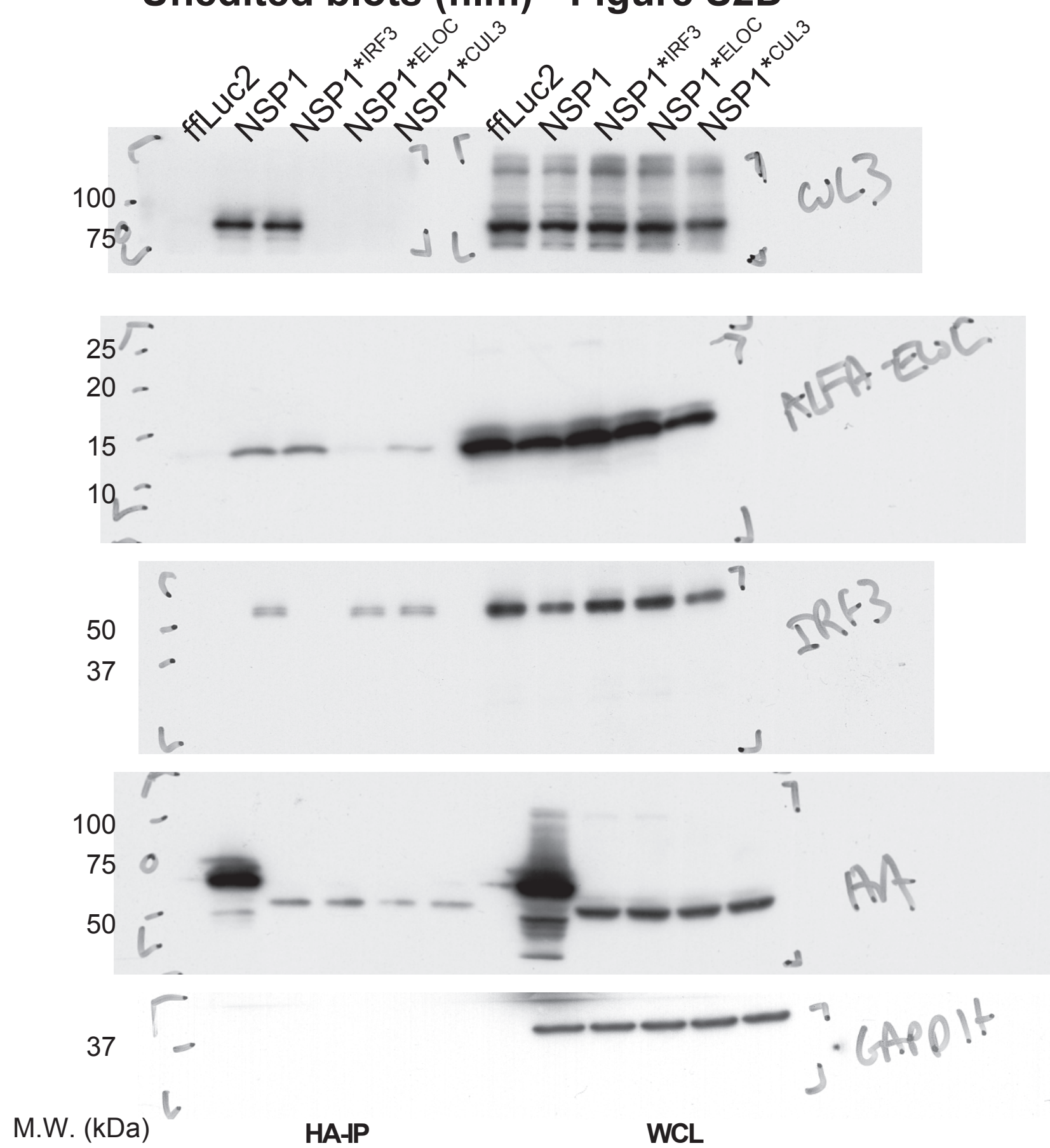

Unedited blots (film) - Figure 3G

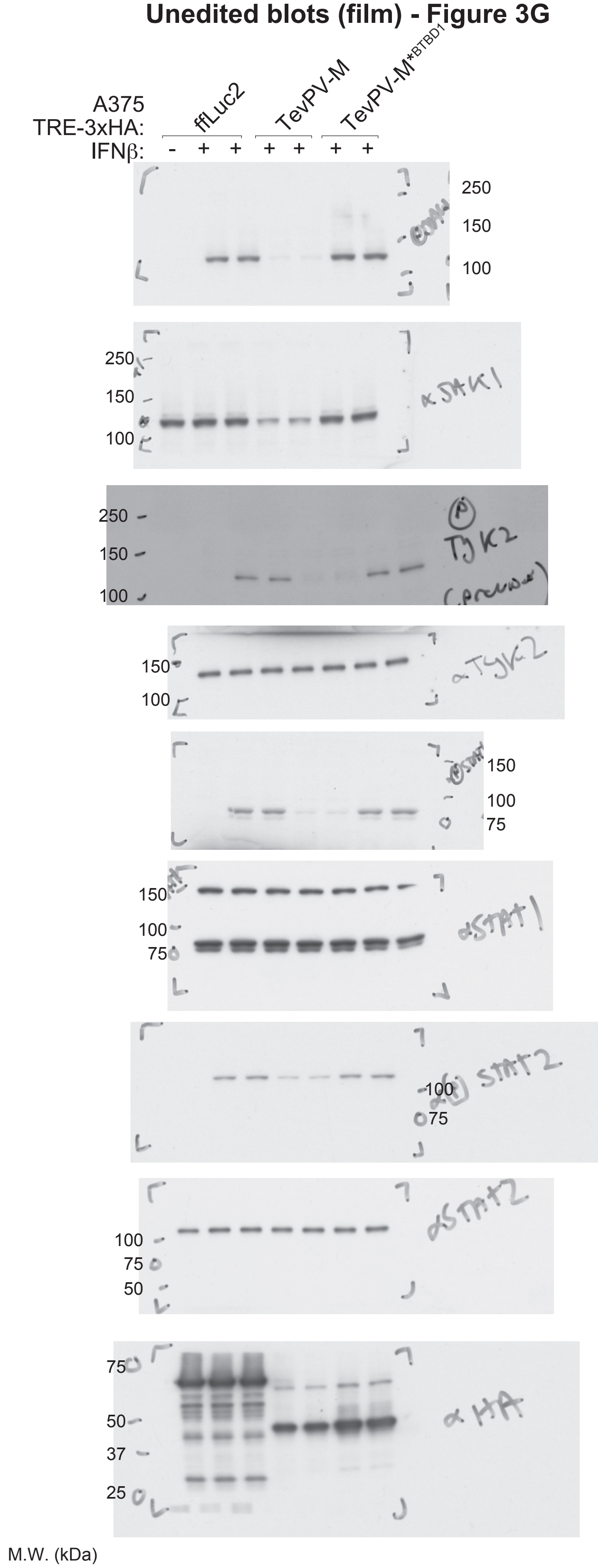

Unedited blots (film) - Figure 3H

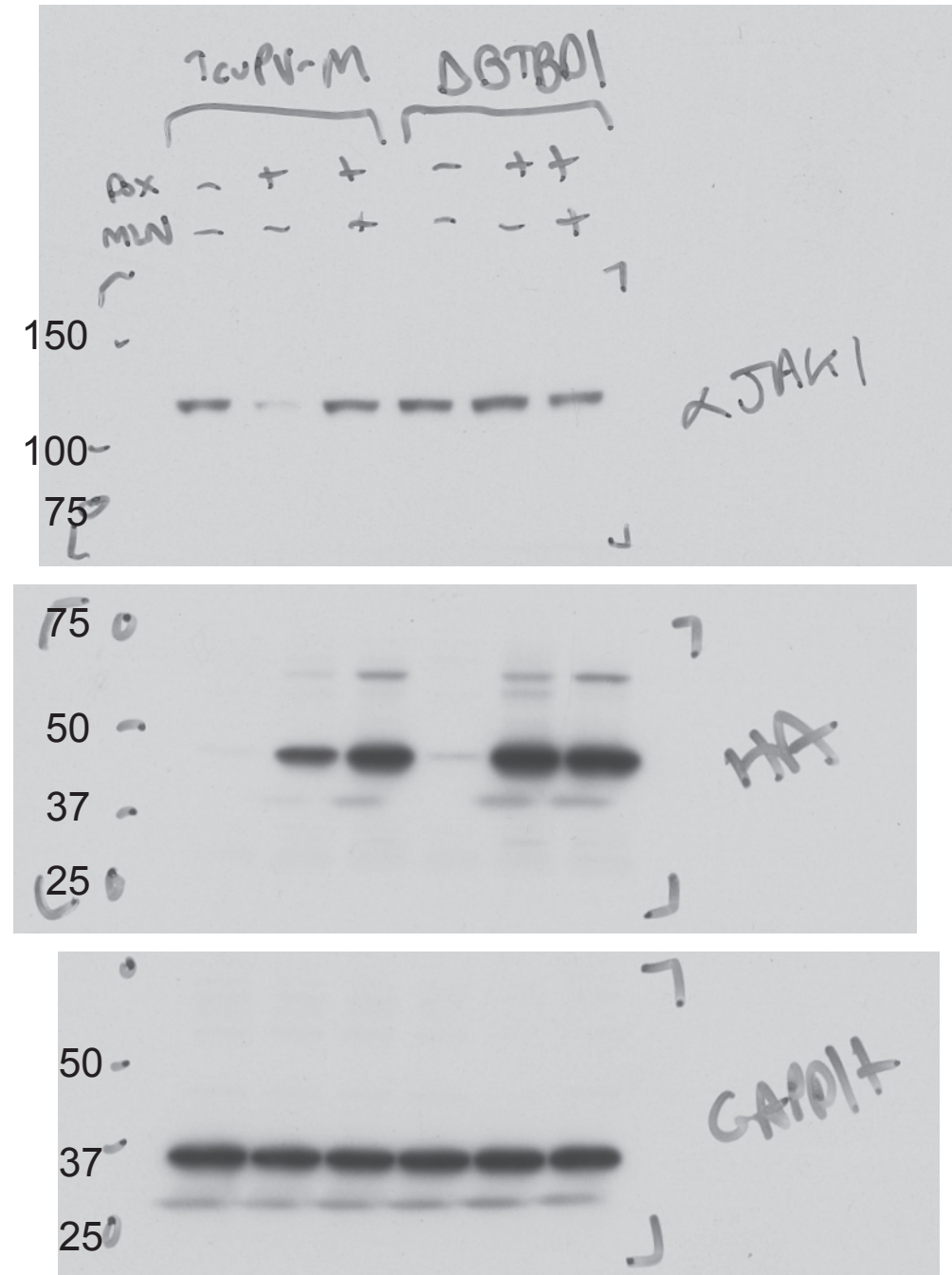

Unedited blots (film) - Figure S3B

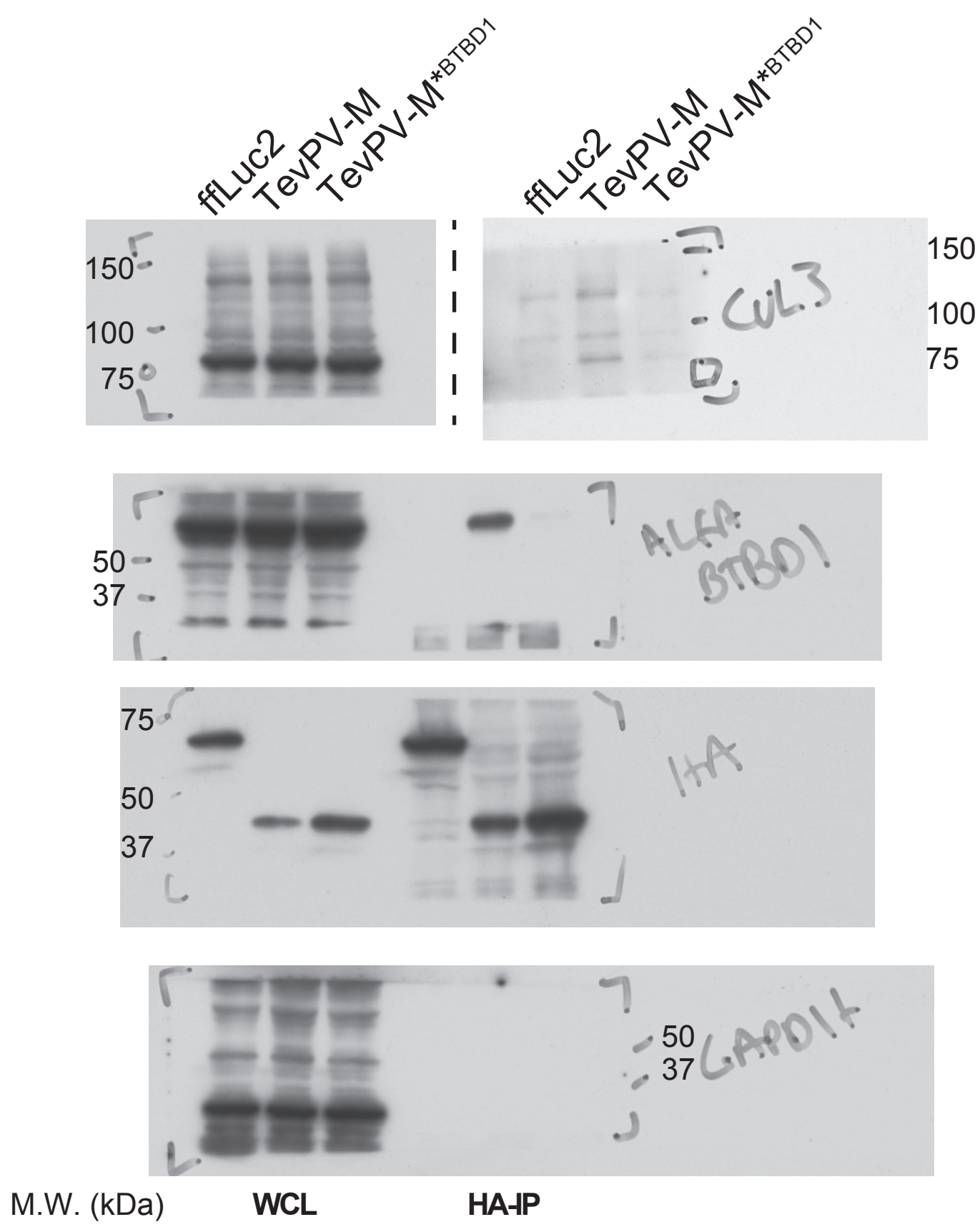

Unedited blots (LICOR) - Figure S3D

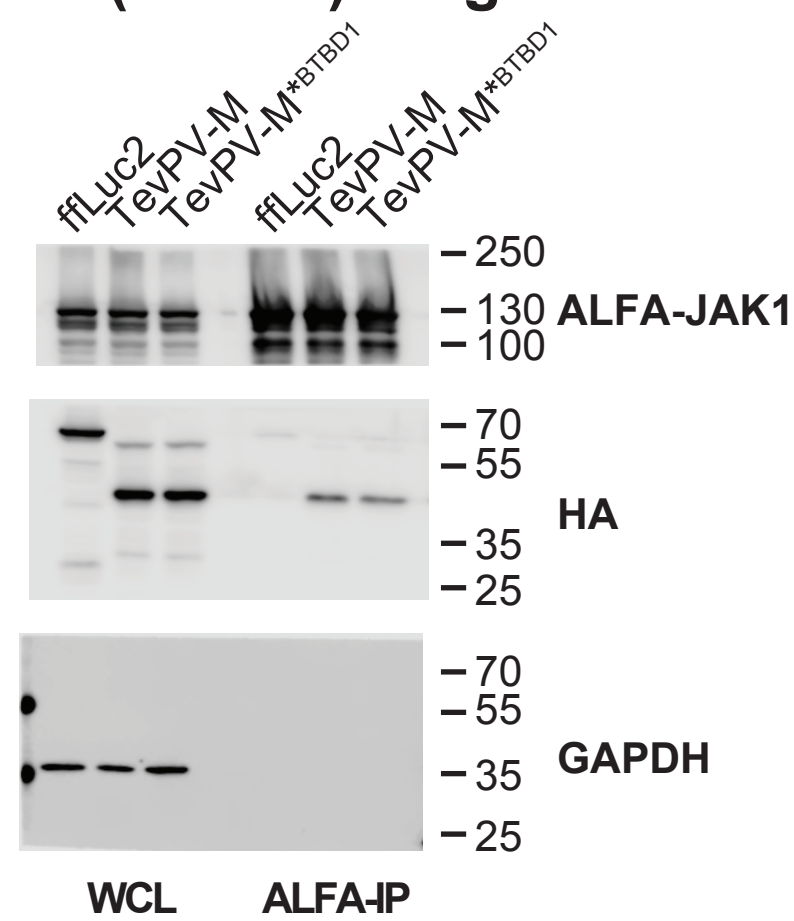

Unedited blots (film) - Figure 4G

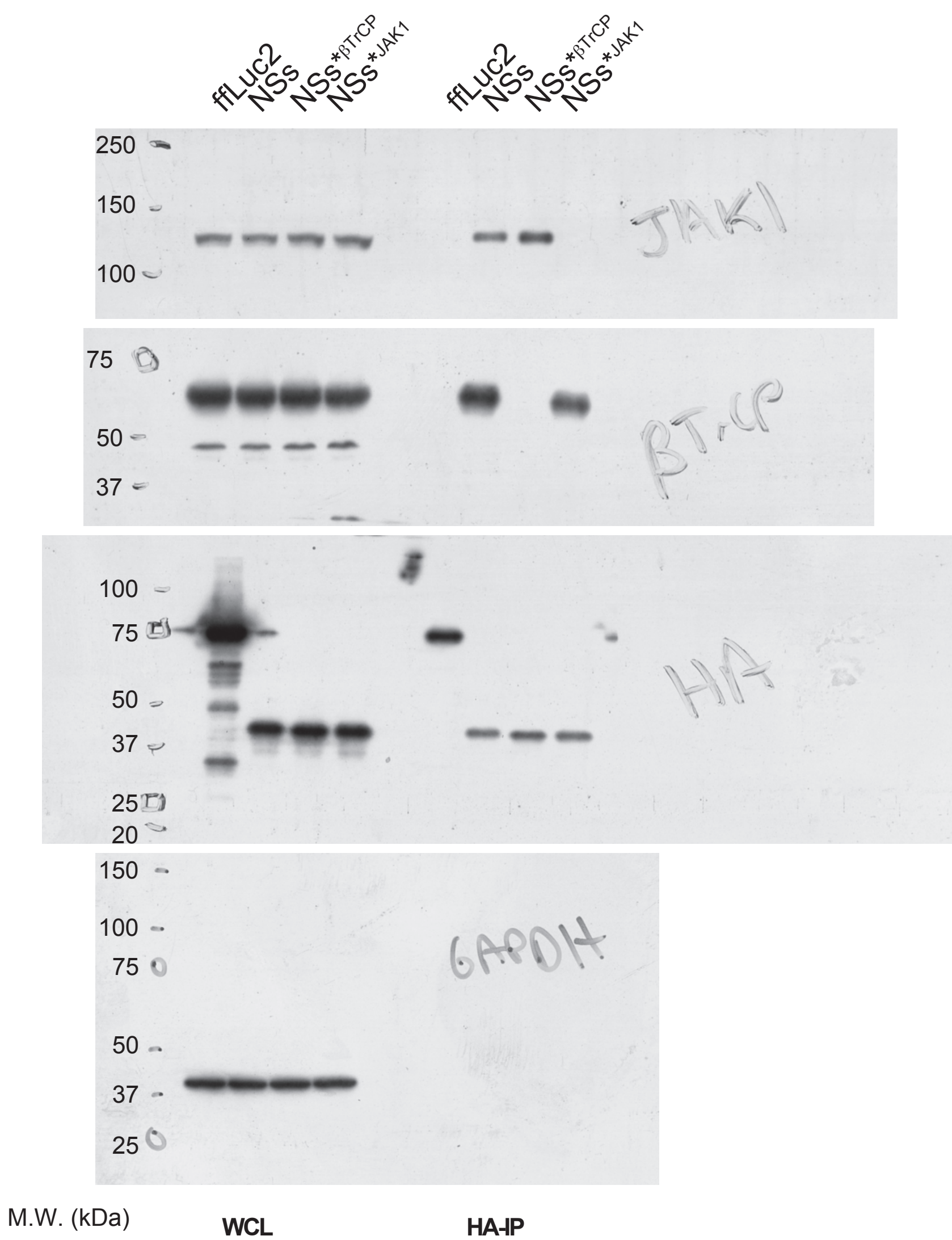

Unedited blots (film) - Figure 4H

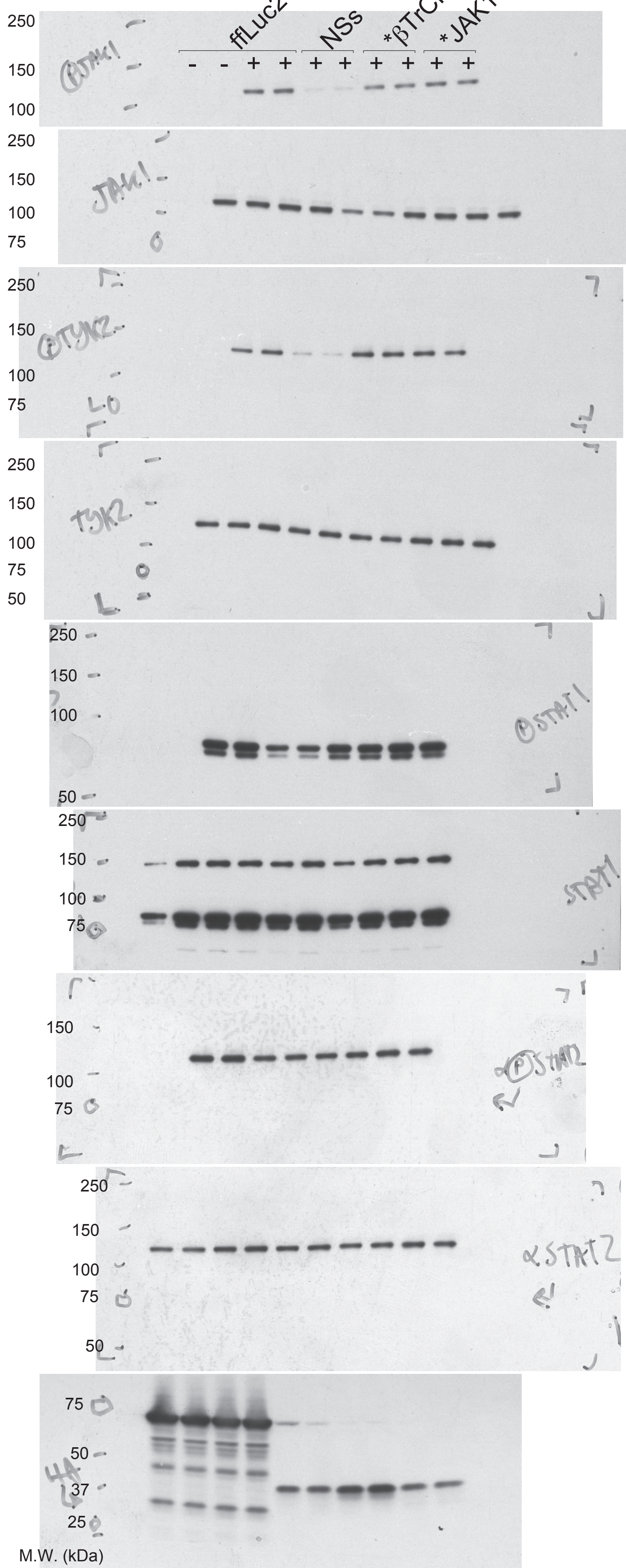

Unedited blots (film) - Figure 5F

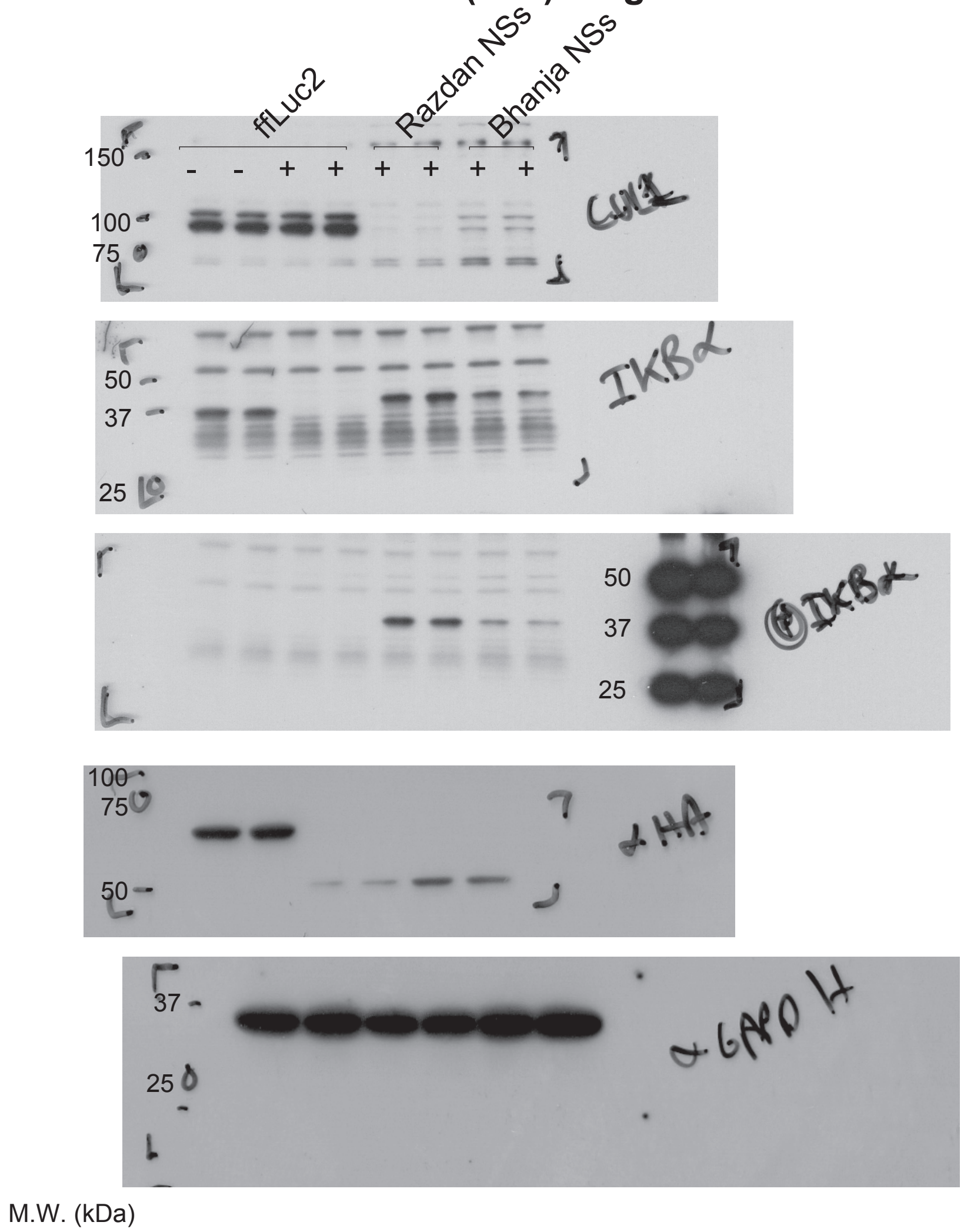

Unedited blots (LICOR) - Figure S5E

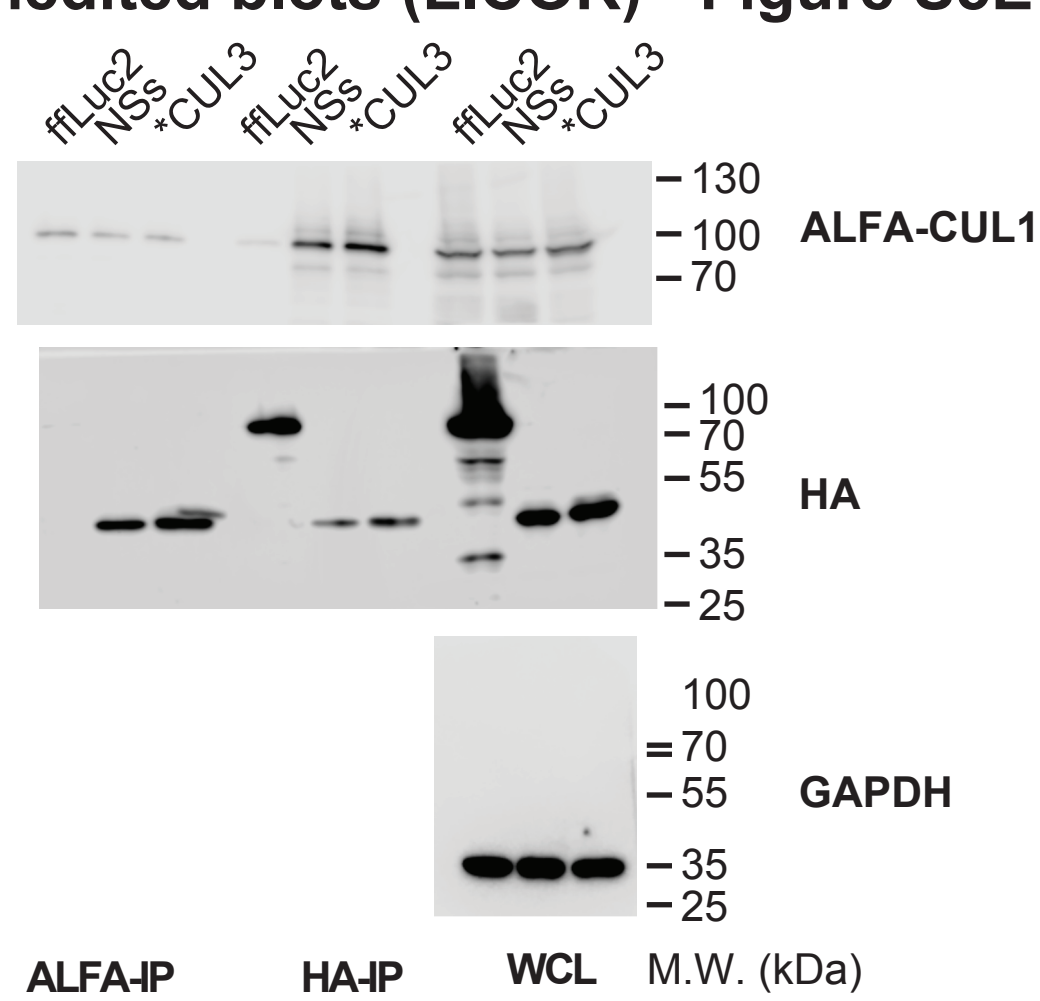

Unedited blots (LICOR) - Figure S5F

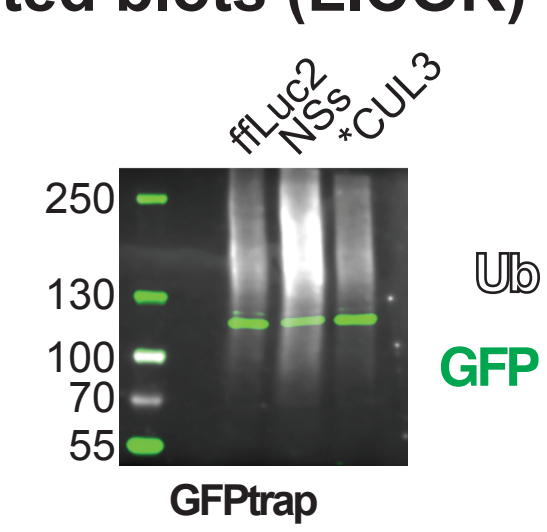

Unedited blots (LICOR) - Figure S6D

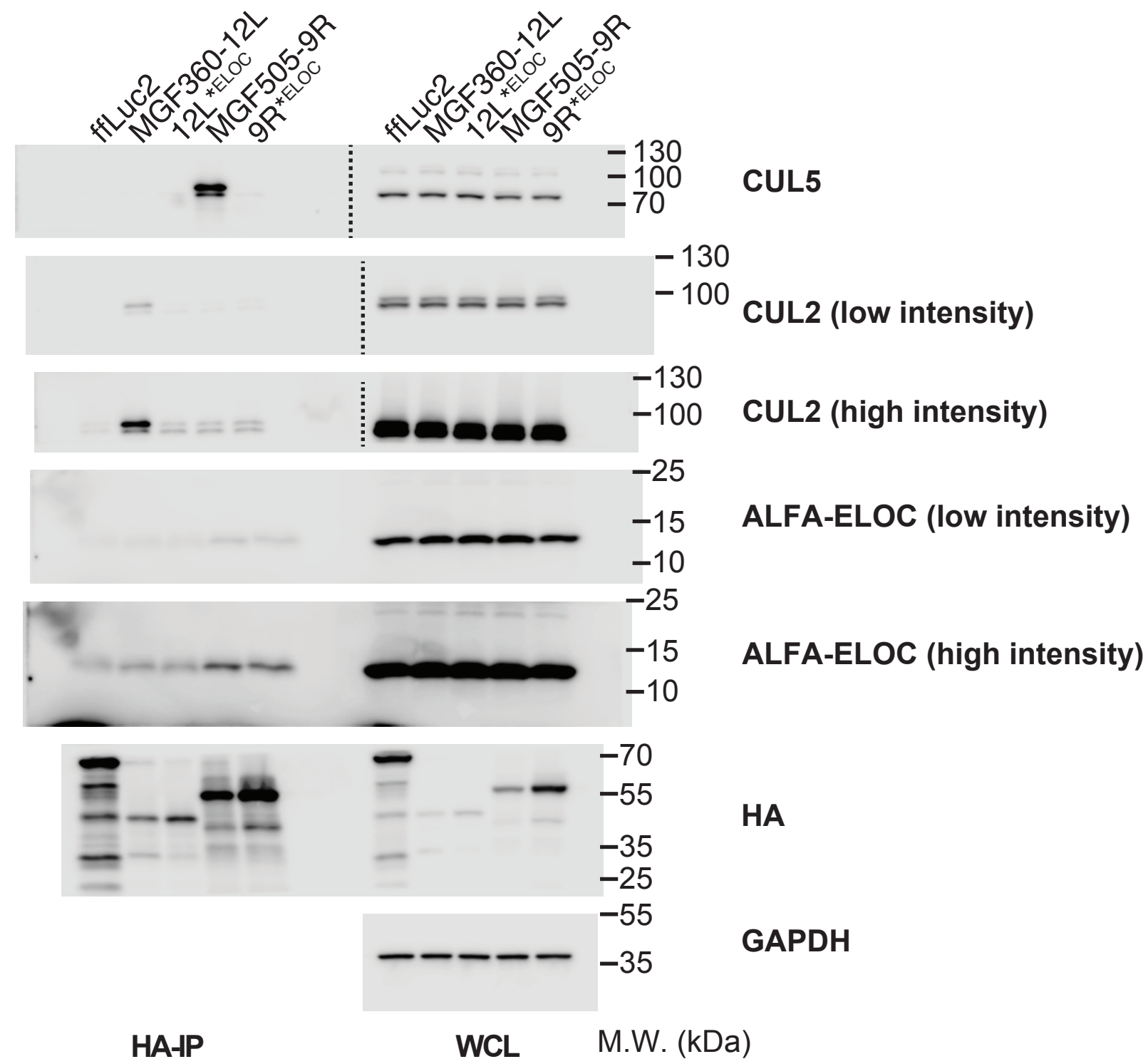

Unedited blots (film) - Figure 7C

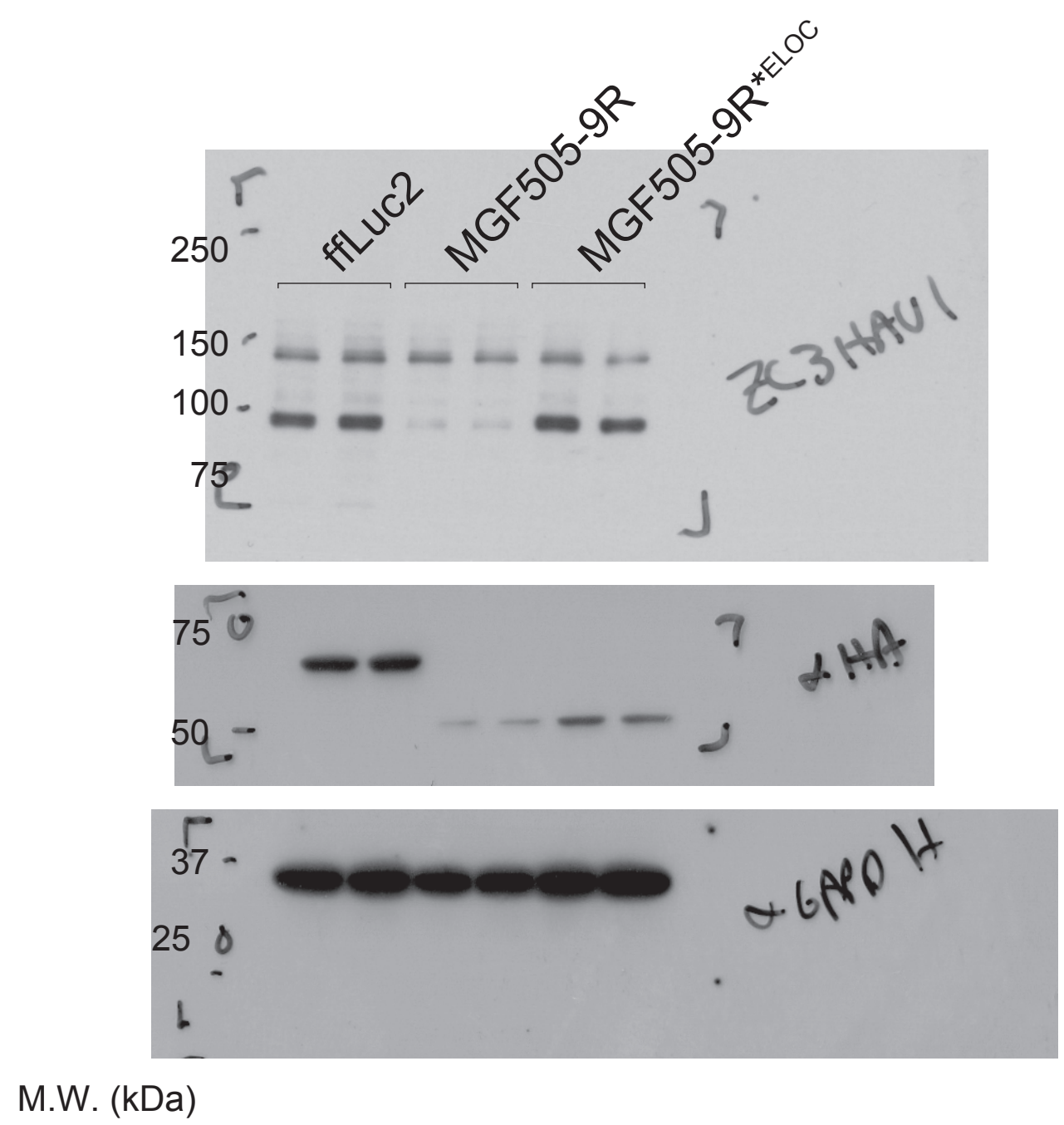

Unedited blots (film) - Figure 7G

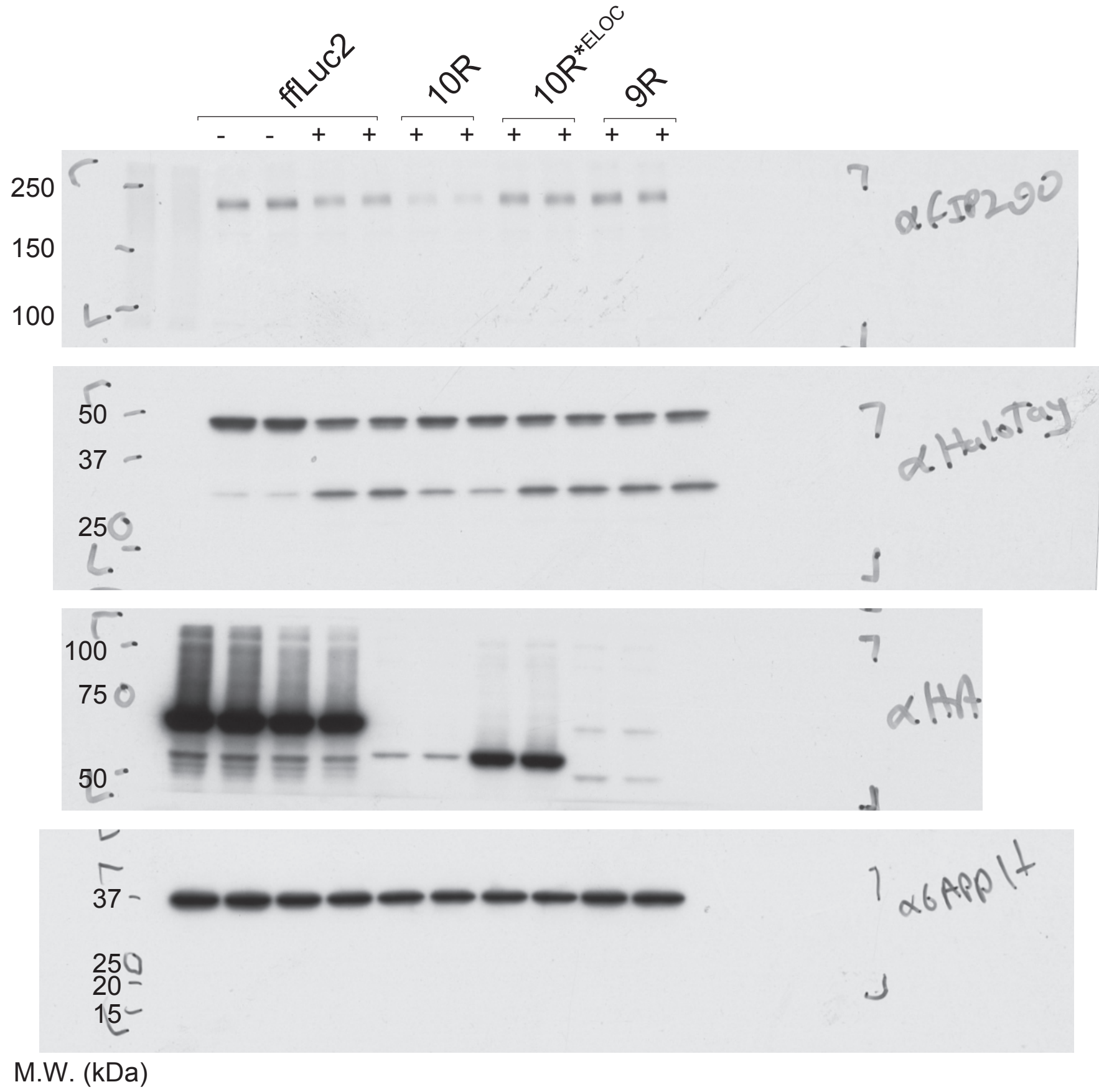

Unedited blots (film) - Figure S7A and S8A

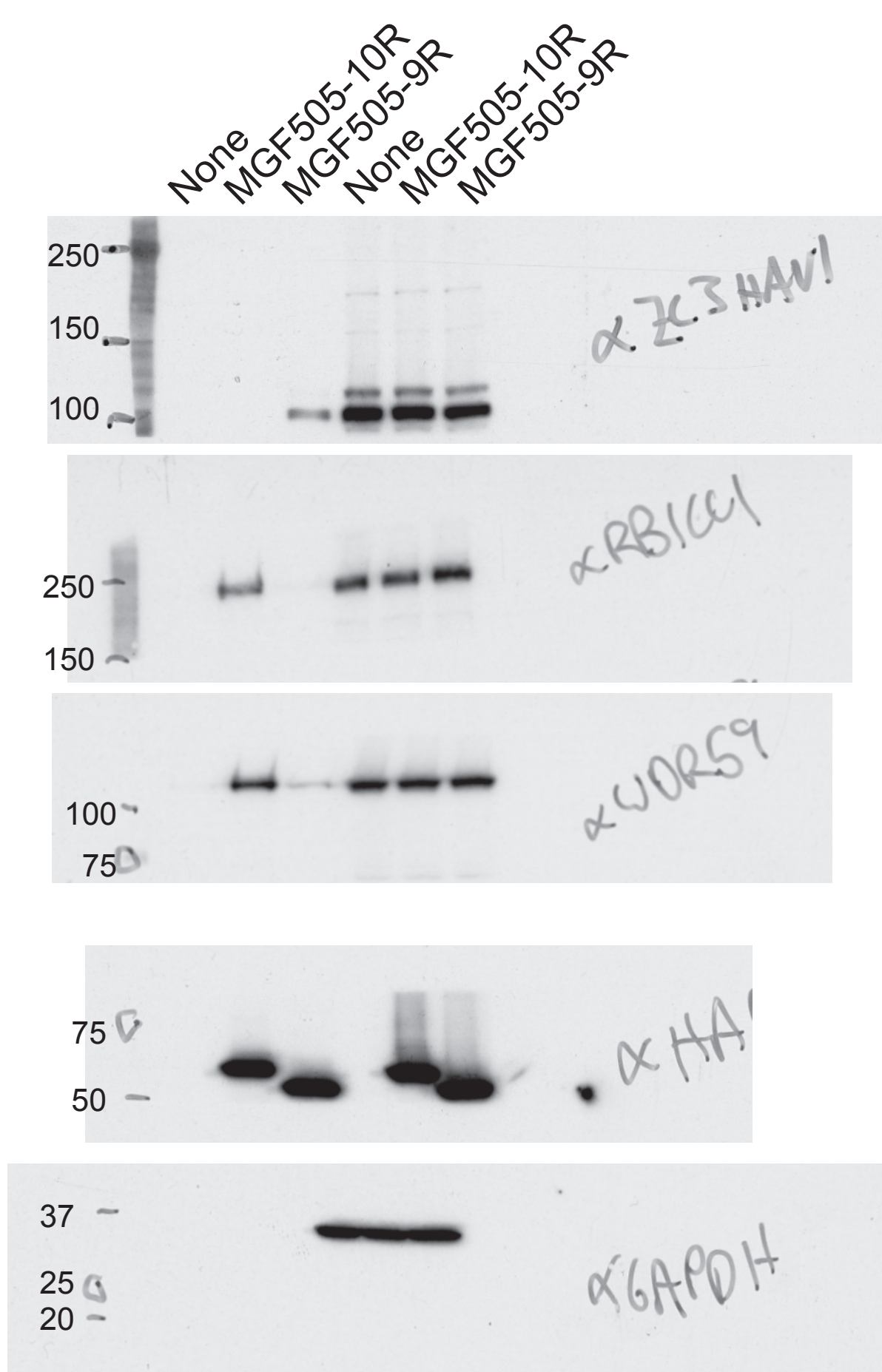

Unedited blots (film) - Figure S7B

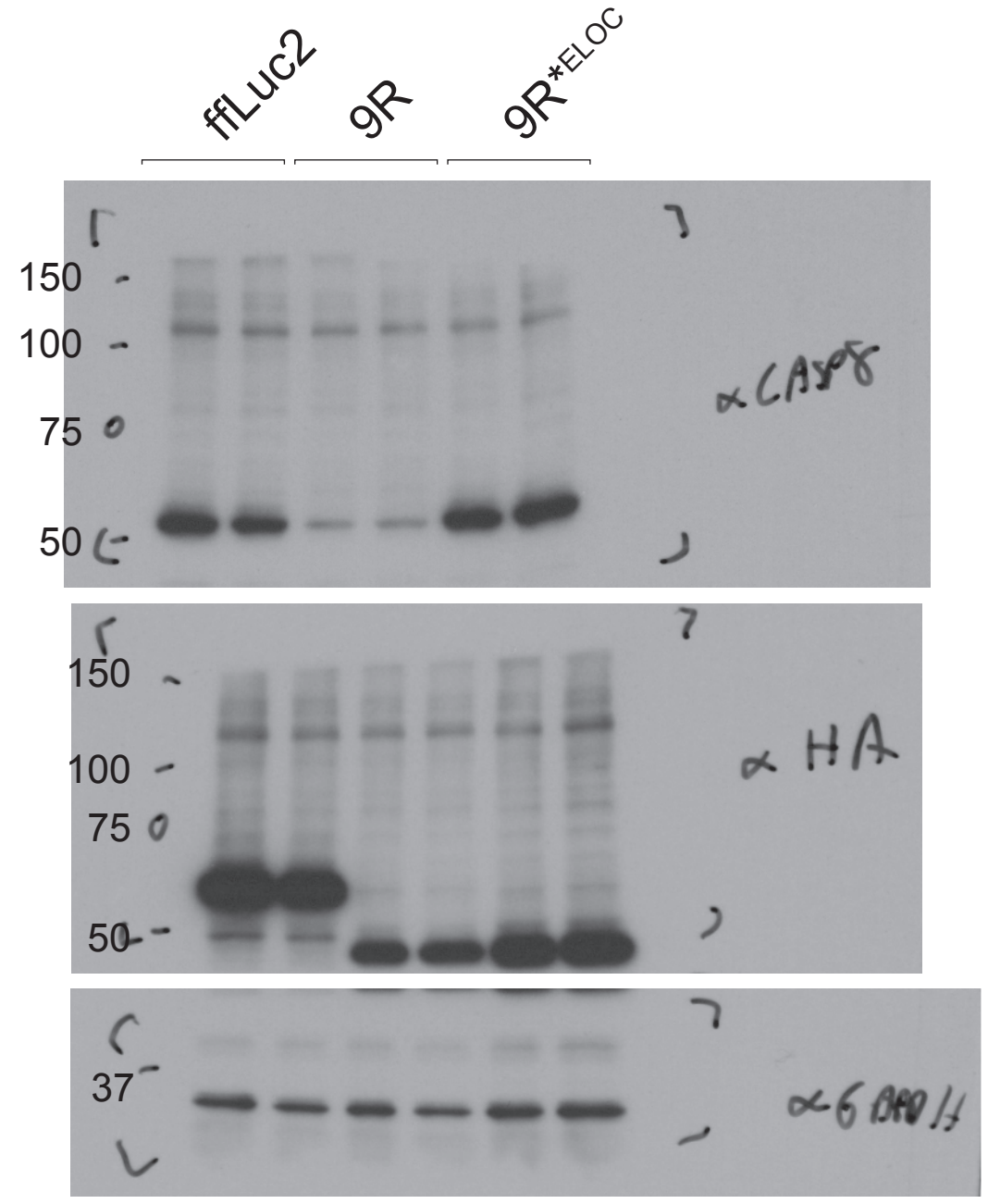

Unedited blots (film) - Figure S7D

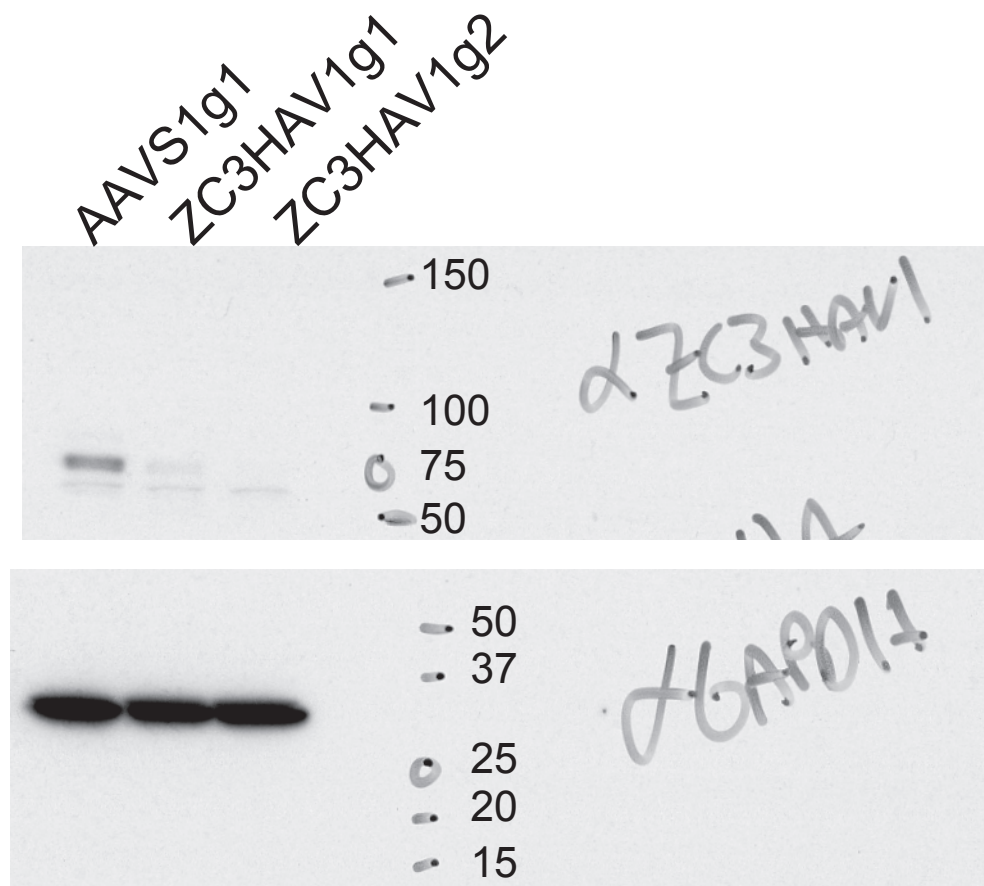

Unedited blots (film) - Figure S8B

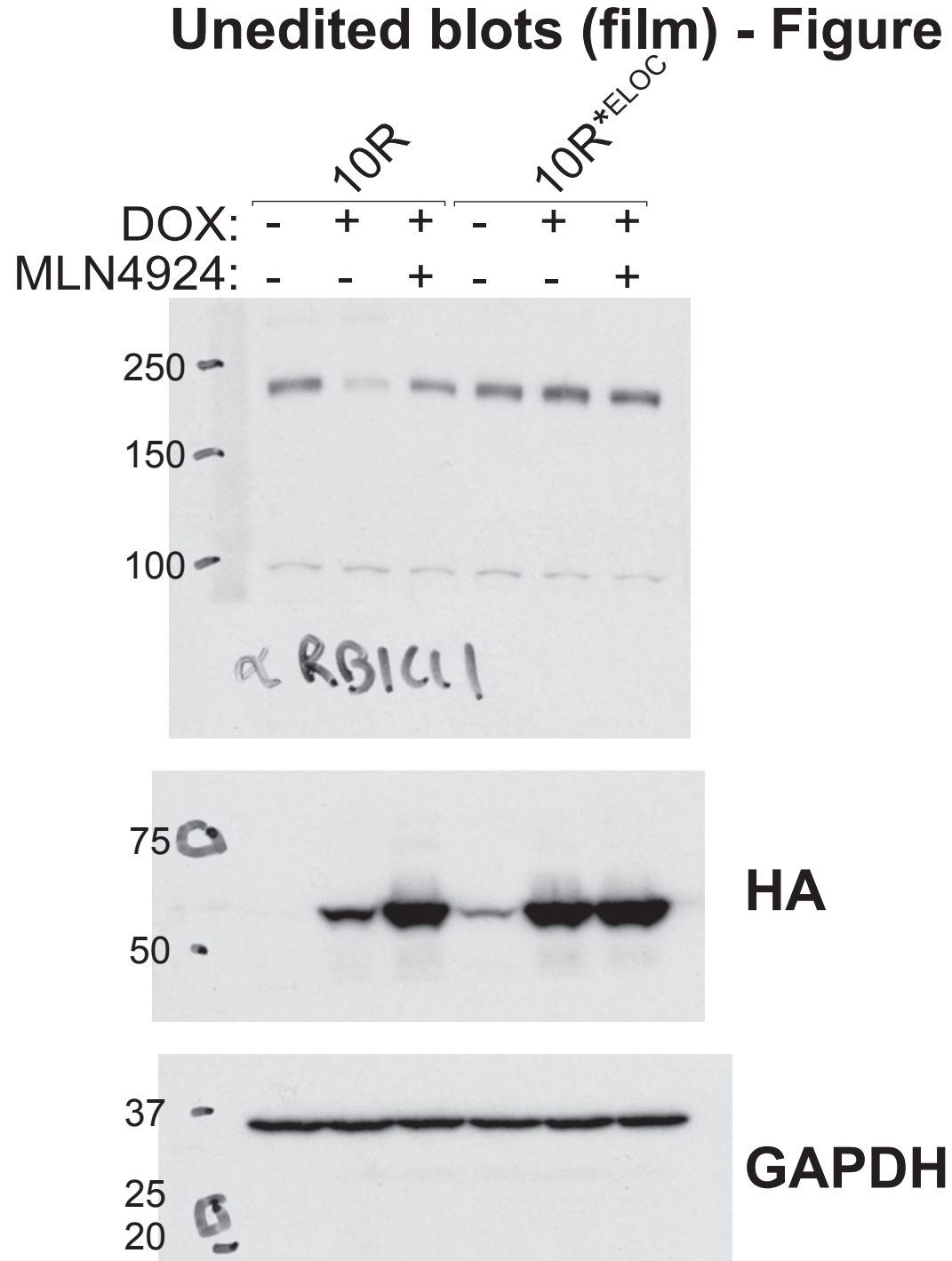

Unedited blots (film) - Figure S8C

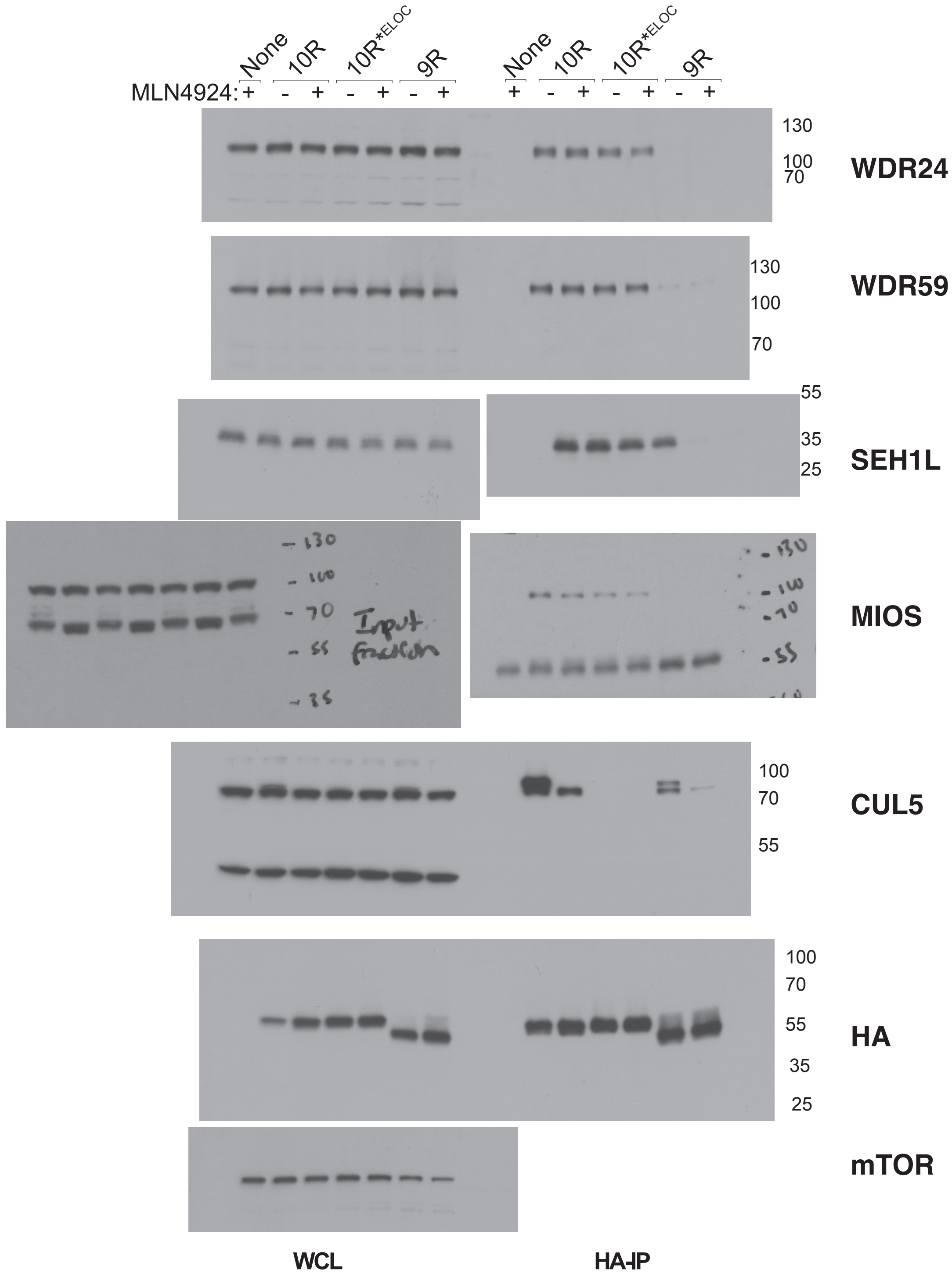

Unedited blots (film) - Figure S8D

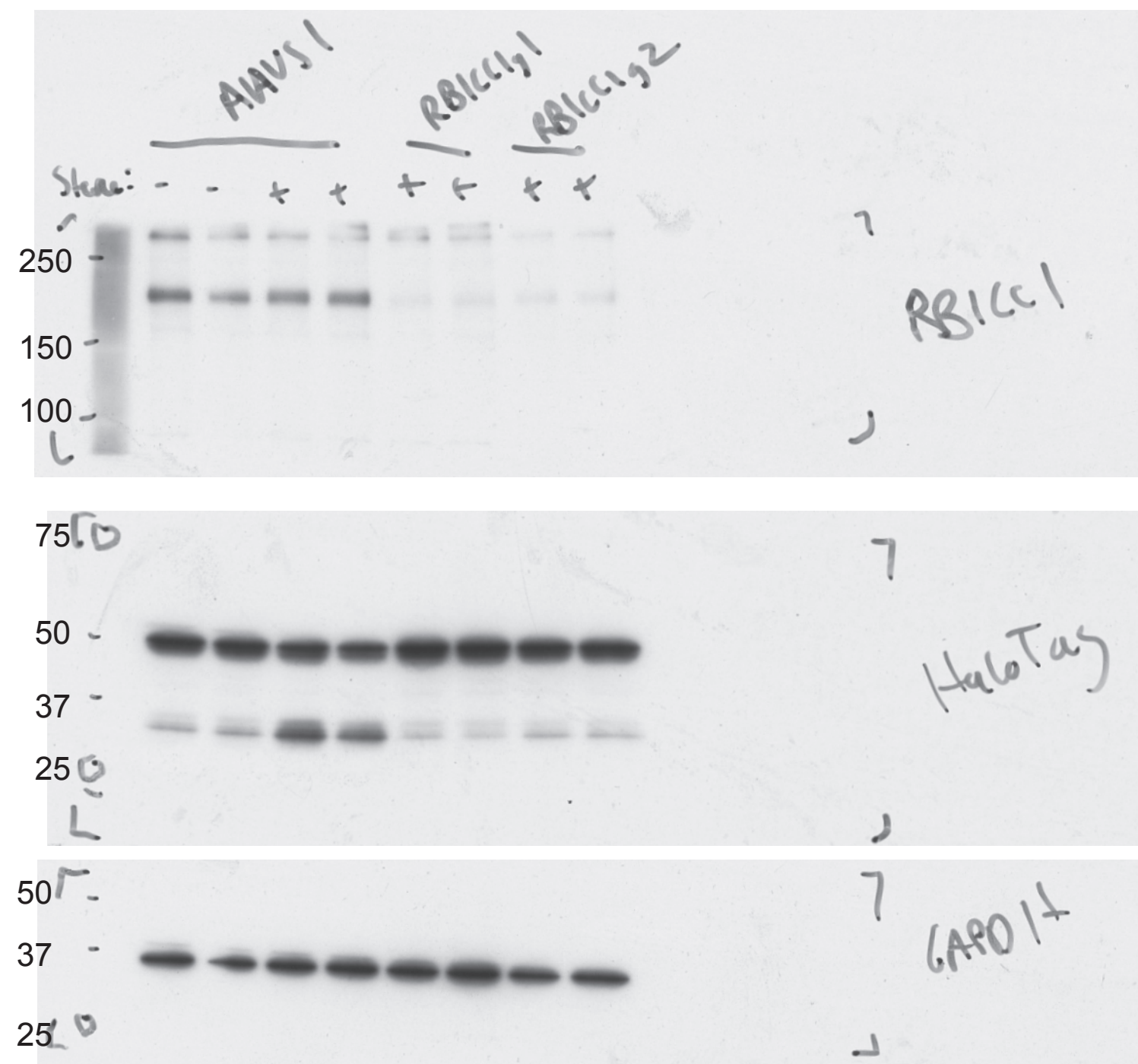

Supplement: Supplementary file 1 [file NIHMS2214919-supplement-DataS8_rawWB.pdf]
