# Supplementary material for: Virome-wide ubiquitin ligase discovery reveals diverse mechanisms of immune evasion
Source: Science. Author manuscript; Available in PMC 2026 Nov 14. (PMC13769053; doi:10.1126/science.aec6299)
Supplement: Supplementary file 2 [file NIHMS2214919-supplement-Glassman_2026_supplement.pdf]

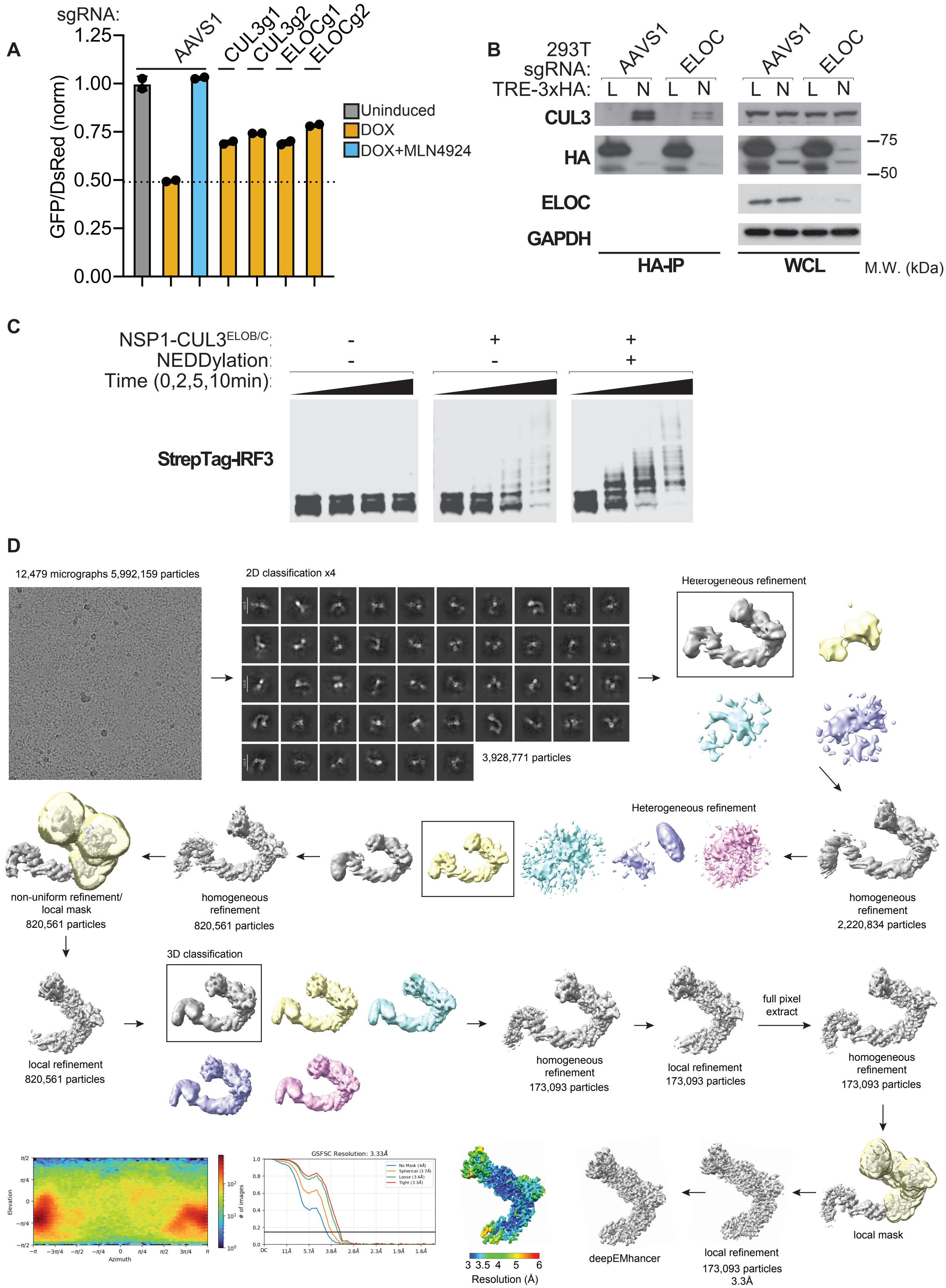
A
sgRNA:
AAVS1
CUL3g1
CUL3g2
ELOCg1
ELOCg2
GFP/DsRed (norm)
1.25
1.00
0.75
0.50
0.25
0.00
Uninduced
DOX
DOX+MLN4924
B
293T
sgRNA:
TRE-3xHA:
AAVS1
ELOC
L N L N L N L N
CUL3
HA
ELOC
GAPDH
HA-IP
WCL
75
50
M.W. (kDa)
C
NSP1-CUL3ELOB/C:
NEDDylation:
Time (0,2,5,10min):
- + +
- - +
StrepTag-IRF3
D
12,479 micrographs 5,992,159 particles
2D classification x4
3,928,771 particles
Heterogeneous refinement
homogeneous refinement
2,220,834 particles
Heterogeneous refinement
homogeneous refinement
820,561 particles
non-uniform refinement/ local mask
820,561 particles
local refinement
820,561 particles
3D classification
homogeneous refinement
173,093 particles
local refinement
173,093 particles
full pixel extract
homogeneous refinement
173,093 particles
local mask
local refinement
173,093 particles
3.3Å
deepEMhancer
Resolution (Å)
3 3.5 4 5 6
GSFSC Resolution: 3.33Å
Elevation
Azimuth
# of images

fig. S1. Characterization of Rotavirus A NSP1, related to Fig. 2.
(A) NSP1-mediated degradation is reduced by Cas9 mutation of CUL3 or ELOC. 293T-pINDUCER20 GPS reporter cells harboring TRE-αGFPnb-NSP1 (ID: Q3ZK61.0.1) were transduced with lentiviral vectors expressing Cas9 and guide RNAs targeting AAVS1 control, CUL3, or ELOC. Cells were treated in duplicate with 200 ng/mL doxycycline (DOX) with or without 1 μM MLN4924 for 16 h prior to fluorescence measurement by flow cytometry. Results are representative of two independent experiments.
(B) ELOC expression is required for NSP1 interaction with CUL3. 293T-pINDUCER20 cells with TRE-3xHA-ffLuc2 (L) or TRE-3xHA-NSP1 (N) were transduced with lentiviral CRISPR constructs targeting AAVS1 control or ELOC and treated with 200 ng/mL DOX for 16 h prior to HA-IP and western blotting. Results are representative of two independent experiments.
(C) NSP1 ubiquitinates IRF3 *in vitro*. CUL3-RBX1-ELOB-ELOC-NSP1 (ID: Q3ZK61.0.1) with or without NEDDylation was added to Strep-tagged IRF3. The reaction was initiated by adding ubiquitin and incubated at room temperature for the indicated time, then quenched in sample buffer. Samples were separated by SDS-PAGE and analyzed by western blot.
(D) Workflow of cryo-electron microscopy data processing.

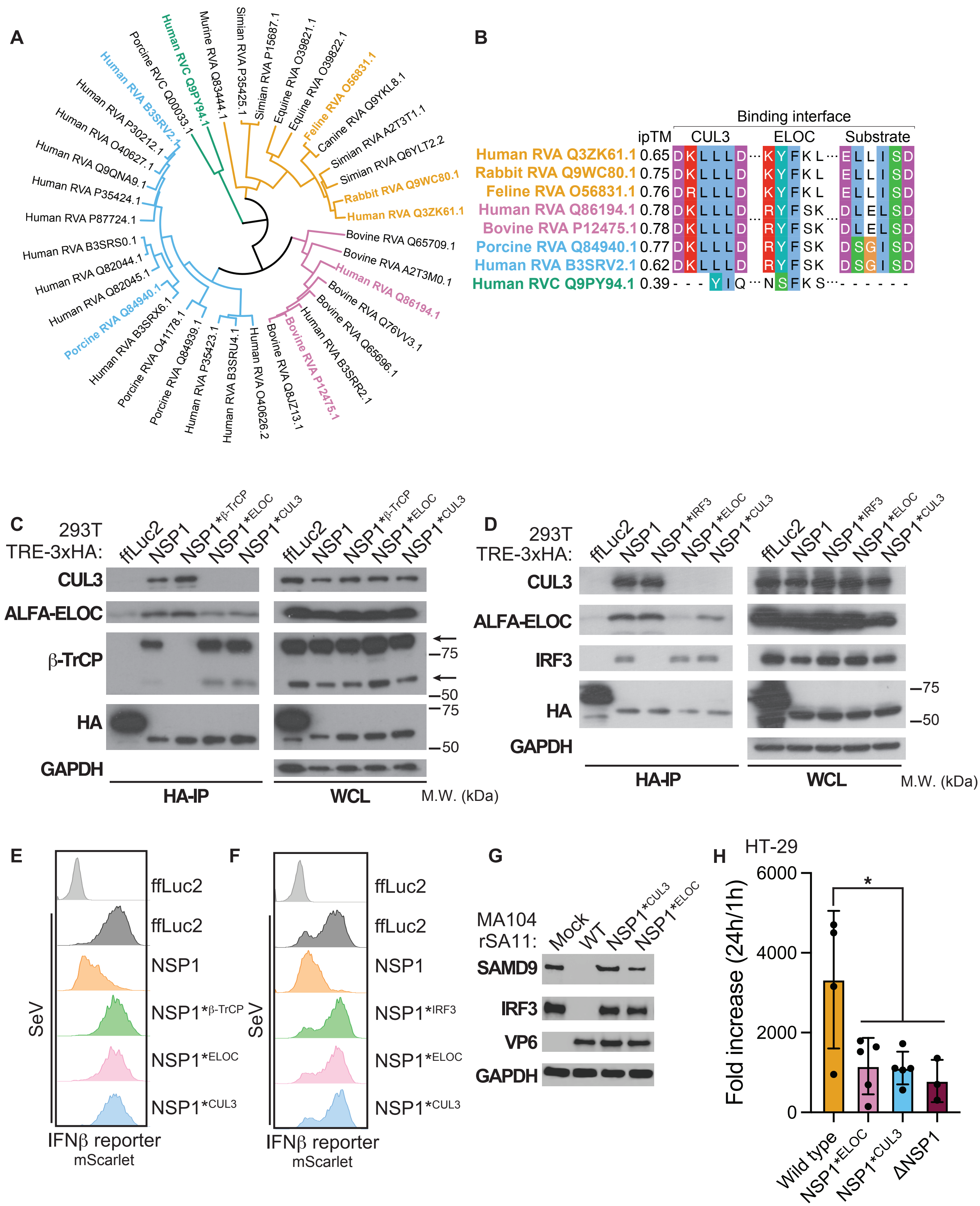

A
Human RVC Q9PY94.1
Porcine RVC Q00033.1
Murine RVA Q83444.1
Simian RVA P35425.1
Simian RVA P15687.1
Equine RVA O39821.1
Equine RVA O39822.1
Feline RVA O56831.1
Canine RVA Q9YKL8.1
Simian RVA A2T3T1.1
Simian RVA Q6YLT2.2
Rabbit RVA Q9WC80.1
Human RVA Q3ZK61.1
Bovine RVA Q65709.1
Bovine RVA A2T3M0.1
Human RVA Q86194.1
Bovine RVA Q76VV3.1
Bovine RVA Q65696.1
Human RVA B3SRR2.1
Bovine RVA P12475.1
Bovine RVA Q8JZ13.1
Human RVA O40626.2
Human RVA B3SRU4.1
Human RVA P35423.1
Porcine RVA Q84939.1
Porcine RVA O41178.1
Human RVA B3SRX6.1
Porcine RVA Q84940.1
Human RVA Q82045.1
Human RVA Q82044.1
Human RVA B3SRS0.1
Human RVA P87724.1
Human RVA P35424.1
Human RVA Q9QNA9.1
Human RVA O40627.1
Human RVA P30212.1
Human RVA B3SRV2.1
B
Binding interface
ipTM CUL3 ELOC Substrate
Human RVA Q3ZK61.1 0.65 D K L L L D ··· K Y F K L ··· E L L I S D
Rabbit RVA Q9WC80.1 0.75 D K L L L D K Y F K L E L L I S D
Feline RVA O56831.1 0.76 D R L L L D K Y F K L E L L I S D
Human RVA Q86194.1 0.78 D K L L L D R Y F S K D L E L S D
Bovine RVA P12475.1 0.78 D K L L L D R Y F S K ··· D L E L S D
Porcine RVA Q84940.1 0.77 D K L L L D R Y F S K D S G I S D
Human RVA B3SRV2.1 0.62 D K L L L D R Y F S K D S G I S D
Human RVC Q9PY94.1 0.39 - - - Y I Q ··· N S F K S ··· - - - - - -
C
293T
TRE-3xHA:
ffLuc2
NSP1
NSP1*β-TrCP
NSP1*ELOC
NSP1*CUL3
CUL3
ALFA-ELOC
β-TrCP
HA
GAPDH
HA-IP
WCL
M.W. (kDa)
75
50
75
50
D
293T
TRE-3xHA:
ffLuc2
NSP1
NSP1*IRF3
NSP1*ELOC
NSP1*CUL3
CUL3
ALFA-ELOC
IRF3
HA
GAPDH
HA-IP
WCL
M.W. (kDa)
75
50
E
ffLuc2
ffLuc2
NSP1
NSP1*β-TrCP
NSP1*ELOC
NSP1*CUL3
SeV
IFNβ reporter
mScarlet
F
ffLuc2
ffLuc2
NSP1
NSP1*IRF3
NSP1*ELOC
NSP1*CUL3
SeV
IFNβ reporter
mScarlet
G
MA104
rSA11:
Mock
WT
NSP1*CUL3
NSP1*ELOC
SAMD9
IRF3
VP6
GAPDH
H
HT-29
Fold increase (24h/1h)
6000
4000
2000
0
*
Wild type
NSP1*ELOC
NSP1*CUL3
ΔNSP1

fig. S2. Characterization of Rotavirus A NSP1, related to Fig. 2.

(A) Dendrogram of NSP1 genes. Rotavirus A NSP1 (ID: Q3ZK61.0.1) was subjected to three rounds of Position-Specific Iterated BLAST (PSI-BLAST) using the Swiss-Prot database. Hits were filtered to remove incomplete sequences and clustered with MMseqs2 to prevent redundancy (98% sequence identity). Unique hits were aligned using Clustal Omega and visualized using Interactive Tree of Life. RVA – Rotavirus A, RVC – Rotavirus C.

(B) Comparison of Rotavirus NSP1 genes. NSP1 genes were analyzed using AlphaFold 3 with CUL3 and ELOC. The maximum interface predicted template modeling (ipTM) score is shown for three independent seeds. Amino acid alignment from Clustal Omega was visualized using Jalview to show conservation of the $CUL3^{ELOB/C}$ binding region and substrate recruitment domain.

(C) NSP1 (ID: B3SRV2.0.1) binding to $CUL3^{ELOB/C}$ depends on residues at the CUL3/ELOC binding interface and can be uncoupled from substrate binding. 293T-pINDUCER20 GPS-β-TrCP1 (DsRed IRES GFP-BTRC) cells were transduced with lentiviral vectors containing TRE-3xHA-ffLuc2, NSP1 wild-type or mutants (∗β-TrCP: S480A/S483A, ∗ELOC: F210R, ∗CUL3: L184R/L185R) and transfected with CMV-ALFA-ELOC. Five hours after transfection, cells were treated with 200 ng/mL DOX and 1 μM MLN4924 for 16 h before lysis and HA-IP. Upper arrow indicates GFP-β-TrCP and lower arrow indicates endogenous β-TrCP.

(D) NSP1 (ID: Q3ZK61.0.1) binding to $CUL3^{ELOB/C}$ is disrupted by mutations at the identified CUL3/ELOC binding interface and can be uncoupled from substrate binding. 293T-pINDUCER20 cells with constructs encoding luciferase control (TRE-3xHA-ffLuc2), NSP1 wild-type or mutants (∗IRF3: L483R, ∗ELOC: F210R, ∗CUL3: L184R/L185R) were transfected with CMV-ALFA-ELOC. 5 h after transfection, cells were treated with 200 ng/mL DOX + 1 μM MLN4924 for 16 h before HA-IP and western blotting with the indicated antibodies.

(E) NSP1 suppression of IFNβ induction depends on the C-terminal β-TrCP degron and CUL3/ELOC interacting residues. A375-pINDUCER20 cells harboring an IFNβ reporter and DOX-inducible vectors described in C were treated for 24 h with 200 ng/mL DOX prior to infection with 1:100 Sendai virus (SeV). Fluorescence was measured 14 h post-infection by flow cytometry.

(F) NSP1 suppression of IFNβ induction depends on interactions with IRF3 and $CUL3^{ELOB/C}$. A375-pINDUCER20 cells harboring an IFNβ reporter and DOX-inducible constructs described in D were treated for 24 h with 200 ng/mL DOX prior to infection with 1:100 SeV. Fluorescence was measured 14 h post-infection by flow cytometry. Experimental results are representative of two or more independent experiments.

(G) NSP1 mutants display attenuated substrate degradation during live virus infection. MA104 cells were infected with Rotavirus SA11 (rSA11) harboring wild-type NSP1 or mutants (∗CUL3: L185R/L186R, ∗ELOC: F211R) at a multiplicity of infection (MOI) of 5 for 8 h prior to harvesting lysates for western blot analysis with the indicated antibodies.

(H) SA11 viral titers, related to Fig. 2H. Fold change was calculated between 1 and 24 h time points and analyzed by one-way ANOVA with Dunnett's post-test (* $p < 0.05$).

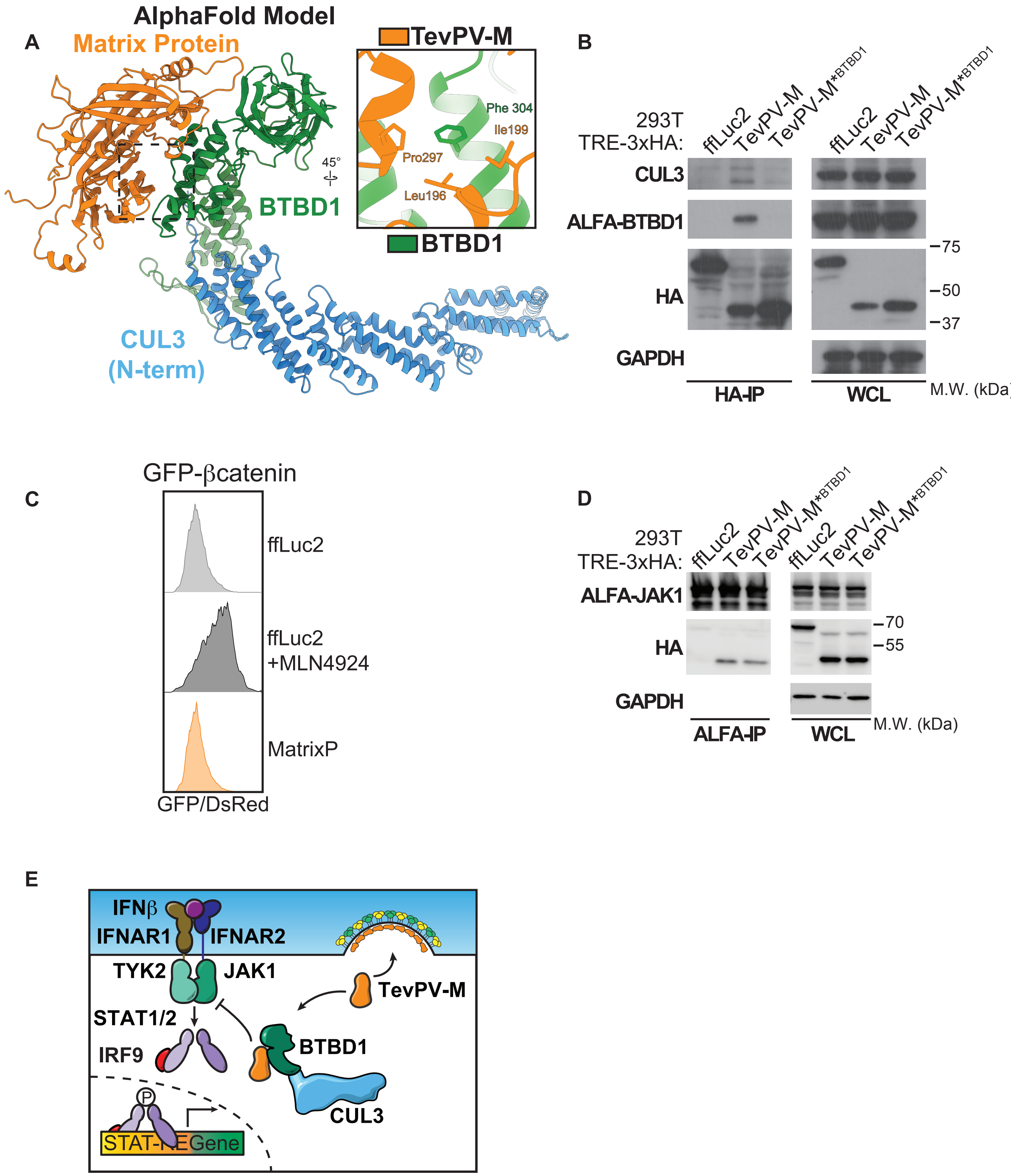

A
AlphaFold Model
Matrix Protein
BTBD1
CUL3
(N-term)
45°
TevPV-M
BTBD1
Phe 304
Ile199
Pro297
Leu196
B
293T
TRE-3xHA:
ffLuc2
TevPV-M
TevPV-M*BTBD1
CUL3
ALFA-BTBD1
HA
GAPDH
HA-IP
WCL
75
50
37
M.W. (kDa)
C
GFP-βcatenin
ffLuc2
ffLuc2
+MLN4924
MatrixP
GFP/DsRed
D
293T
TRE-3xHA:
ffLuc2
TevPV-M
TevPV-M*BTBD1
ALFA-JAK1
HA
GAPDH
ALFA-IP
WCL
70
55
M.W. (kDa)
E
IFNβ
IFNAR1
IFNAR2
TYK2
JAK1
STAT1/2
IRF9
TevPV-M
BTBD1
CUL3
P
STAT-RE
Gene

fig. S3. Characterization of Teviot virus matrix protein (TevPV-M), related to Fig. 3.
(A) AlphaFold 3 prediction of TevPV-M (ID: YP_009176989.1.1), CUL3 N-terminus (1-418), and BTBD1 (ipTM = 0.62).
(B) TevPV-M binding to $CUL3^{BTBD1}$ depends on residues at the predicted binding interface. 293T-pINDUCER20 cells stably expressing PGK-ALFA-BTBD1 were transduced with TRE-3xHA-ffLuc2, TevPV-M wild-type or mutant (∗BTBD1:L196E/I199E/P297F). Expression was induced with 200 ng/mL DOX for 16 h prior to HA immunoprecipitation (HA-IP) and western blotting.
(C) TevPV-M does not impact β-catenin stability. 293T-pINDUCER20 cells expressing GPS-β-catenin (DsRed IRES GFP-CTNNB1) were transduced with TRE-3xHA-ffLuc2 or TevPV-M and treated with 200 ng/mL DOX with or without 1 μM MLN4924 for 16 h prior to analysis by flow cytometry for a single experiment.
(D) TevPV-M interacts with JAK1. 293T-pINDUCER20 cells harboring TRE-3xHA-ffLuc2 or TevPV-M wild-type or mutant were transfected with CMV-ALFA-JAK1. Five hours after transfection, cells were treated with 200 ng/mL DOX + 1 μM MLN4924 for 16 h before ALFA-IP and western blotting with the indicated antibodies. Results are representative of two or more independent experiments unless otherwise noted.
(E) Schematic of TevPV-M function. TevPV-M is incorporated into the $CUL3^{BTBD1}$ complex to regulate IFNβ signaling by promoting JAK1 degradation; however, this interaction reduces TevPV-M stability, limiting its availability for virion assembly.

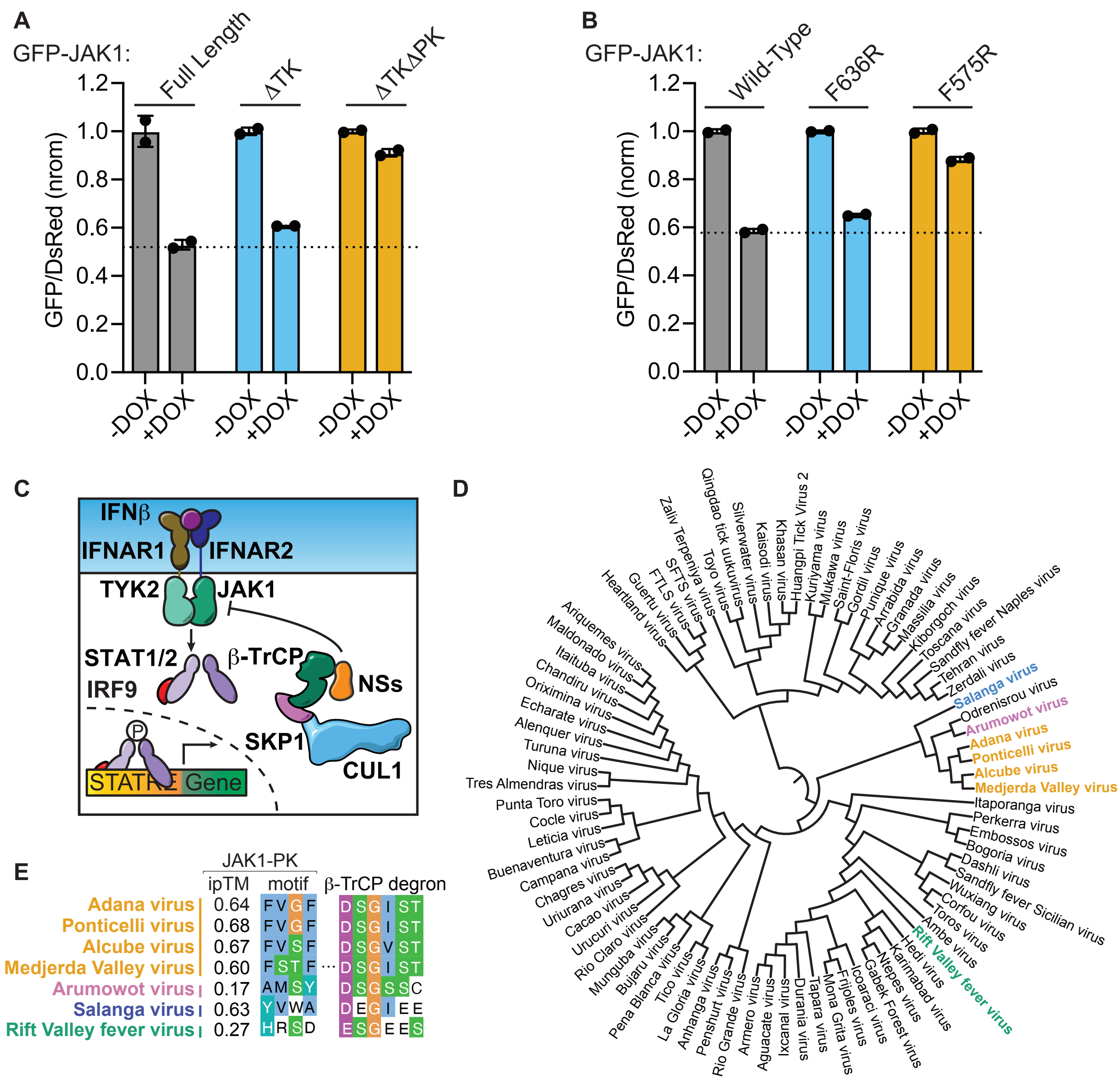
A
GFP-JAK1:
Full Length
ΔTK
ΔTKΔPK
GFP/DsRed (nrom)
1.2
1.0
0.8
0.6
0.4
0.2
0.0
-DOX
+DOX
B
GFP-JAK1:
Wild-Type
F636R
F575R
GFP/DsRed (norm)
C
IFNβ
IFNAR1
IFNAR2
TYK2
JAK1
STAT1/2
β-TrCP
IRF9
NSs
SKP1
CUL1
P
STATRE
Gene
D
Zaliv Terpeniya virus
Qingdao tick uukuvirus
Silverwater virus
Kaisodi virus
Khasan virus
Huangpi Tick Virus 2
Kuriyama virus
Mukawa virus
Saint-Floris virus
Gordil virus
Punique virus
Arrabida virus
Granada virus
Massilia virus
Kiborgoch virus
Toscana virus
Sandfly fever Naples virus
Tehran virus
Zerdali virus
Salanga virus
Odrenisrou virus
Arumowot virus
Adana virus
Ponticelli virus
Alcube virus
Medjerda Valley virus
Itaporanga virus
Perkerra virus
Embossos virus
Bogoria virus
Dashli virus
Sandfly fever Sicilian virus
Wuxiang virus
Corfou virus
Toros virus
Ambe virus
Rift Valley fever virus
Hedi virus
Karimabad virus
Ntepes virus
Gabek Forest virus
Icoaraci virus
Frijoles virus
Mona Grita virus
Tapara virus
Durania virus
Ixcanal virus
Aguacate virus
Armero virus
Rio Grande virus
Penshurt virus
Anhanga virus
La Gloria virus
Tico virus
Pena Blanca virus
Bujaru virus
Munguba virus
Rio Claro virus
Urucuri virus
Cacao virus
Uriurana virus
Chagres virus
Campana virus
Buenaventura virus
Leticia virus
Cocle virus
Punta Toro virus
Tres Almendras virus
Nique virus
Turuna virus
Alenquer virus
Echarate virus
Oriximina virus
Chandiru virus
Itaituba virus
Maldonado virus
Ariquemes virus
Heartland virus
Guertu virus
FTLS virus
SFTS virus
Toyo virus
E
JAK1-PK
ipTM
motif
β-TrCP degron
Adana virus 0.64 F V G F ... D S G I S T
Ponticelli virus 0.68 F V G F D S G I S T
Alcube virus 0.67 F V S F D S G V S T
Medjerda Valley virus 0.60 F S T F D S G I S T
Arumowot virus 0.17 A M S Y D S G S S C
Salanga virus 0.63 Y V W A D E G I E E
Rift Valley fever virus 0.27 H R S D E S G E E S

fig. S4. Characterization of Adana virus NSs, related to Fig. 4.

(A) NSs-mediated degradation of JAK1 requires the pseudokinase domain. 293T-pINDUCER20 cells expressing GPS-JAK1 (DsRed IRES GFP-JAK1) constructs corresponding to full-length JAK1, deletion of the tyrosine kinase (ΔTK) or deletion of the tyrosine kinase and pseudokinase (ΔTKΔPK) were transduced with TRE-3xHA-NSs (ID: YP_009227129.1.1). Expression was induced in duplicate with 200 ng/mL doxycycline (DOX) for 16 h. Following induction, fluorescence was measured by flow cytometry and displayed as mean ± standard deviation.

(B) Mutations in JAK1 at the predicted NSs binding interface attenuate degradation. 293T-pINDUCER20 cells expressing wild-type GPS-JAK1 or mutants as indicated were transduced with TRE-3xHA-NSs. Expression was induced in duplicate with 200 ng/mL DOX for 16 h and fluorescence was measured by flow cytometry. Data are shown as mean ± standard deviation.

(C) Schematic of NSs-mediated JAK1 degradation.

(D) Dendrogram of NSs-related genes. Adana virus NSs was subjected to three rounds of Position-Specific Iterated BLAST (PSI-BLAST) using the non-redundant protein sequence database. *Phlebovirus* hits were filtered to remove incomplete sequences and clustered using MMseqs2 to prevent redundancy (98% sequence identity). Sequences were aligned using Clustal Omega and visualized using Interactive Tree of Life.

(E) Comparison of NSs-related genes. AlphaFold 3 predictions for viral proteins in complex with JAK1 pseudokinase domain. The maximum interface predicted template modeling (ipTM) score is shown for three independent seeds. Amino acid alignment from Clustal Omega was visualized using Jalview. Experimental results are representative of two or more independent experiments.

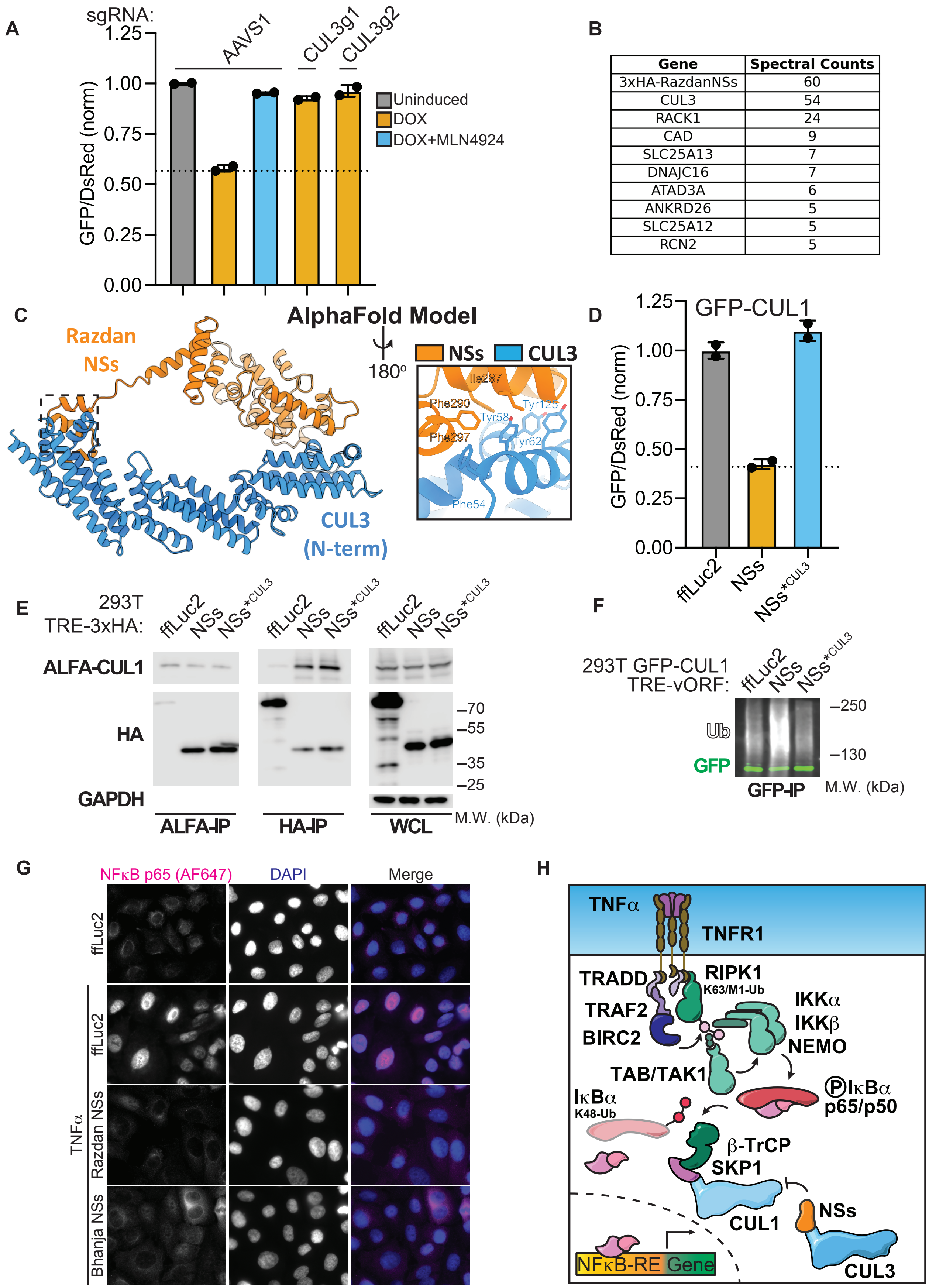

Gene	Spectral Counts
3xHA-RazdanNSs	60
CUL3	54
RACK1	24
CAD	9
SLC25A13	7
DNAJC16	7
ATAD3A	6
ANKRD26	5
SLC25A12	5
RCN2	5

fig. S5. Characterization of Razdan virus NSs, related to Fig. 5.

(A) Razdan NSs-mediated degradation requires CUL3. 293T-pINDUCER20 GPS reporter cells with Cas9 and guide RNA targeting AAVS1 control or CUL3 were treated in duplicate with 200 ng/mL doxycycline (DOX) for 16 h to induce expression of TRE-αGFPnb-NSs (ID: YP_008719919.1.1). Following incubation, fluorescence was measured by flow cytometry and displayed as mean ± standard deviation.

(B) Razdan NSs interacts with CUL3 but not BTB domain containing proteins by immunoprecipitation-mass spectrometry (IP-MS). 293T-pINDUCER20 cells were transduced with TRE-3xHA-NSs, treated with 200 ng/mL DOX + 1 μM MLN4924 for 16 h before performing HA immunoprecipitation (HA-IP), trypsinization, and liquid chromatography-tandem mass spectrometry (LC-MS/MS). Total spectral counts from a single experiment are shown after filtering to remove common contaminants across experiments. See also Data S3D and S3G.

(C) AlphaFold 3 prediction of Razdan NSs and CUL3 N-terminus (1-418) (ipTM = 0.64).

(D) Razdan NSs degradation depends on a critical phenylalanine residue at the predicted CUL3 binding site. 293T-pINDUCER20 GPS-CUL1 (DsRed IRES GFP-CUL1) cells were transduced with lentiviruses containing TRE-3xHA-NSs wild-type or mutant (∗CUL3:F297A) and treated in duplicate with 200 ng/mL DOX for 16 h prior to flow cytometry.

(E) NSs reciprocally interacts with CUL1. 293T-pINDUCER20 cells harboring TRE-3xHA-ffLuc2 or NSs were transfected with CMV-ALFA-CUL1. 5 h after transfection, cells were treated with 200 ng/mL DOX + 1 μM MLN4924 for 16 h before ALFA-IP or HA-IP and western blotting with the indicated antibodies.

(F) NSs ubiquitination of CUL1 depends on interactions with CUL3. 293T-pINDUCER20 cells harboring TRE-3xHA-NSs or ffLuc2 control were transfected with GPS-CUL1 (DsRed IRES GFP-CUL1). Five hours post transfection, media was exchanged for 200 ng/mL DOX for 16 h. In the final hour, 1 μM bortezomib was added to prevent proteasomal degradation. Clarified lysates were subjected to GFP-IP and stringent washing (8 M urea, 1% SDS) prior to western blotting for GFP-CUL1.

(G) NSs expression prevents nuclear translocation of NFκB-p65 (related to Fig. 5G). A375-pINDUCER20 cells were seeded on glass coverslips and treated with 200 ng/mL DOX to induce expression of 3xHA-ffLuc2, Razdan NSs (ID: YP_008719919.1.1), or Bhanja NSs (ID: YP_009141016.1.1) as noted for 16 h followed by stimulation with 100 ng/mL TNFα for 30 min. Cells were fixed, permeabilized, and stained with αNFκB-p65 antibody followed by AF647-labeled secondary antibody and DAPI to identify nuclei.

(H) Schematic of NSs function. NSs binds to CUL3 to regulate NFκB signaling through degradation of CUL1. Experimental results are representative of two or more independent experiments unless otherwise noted.

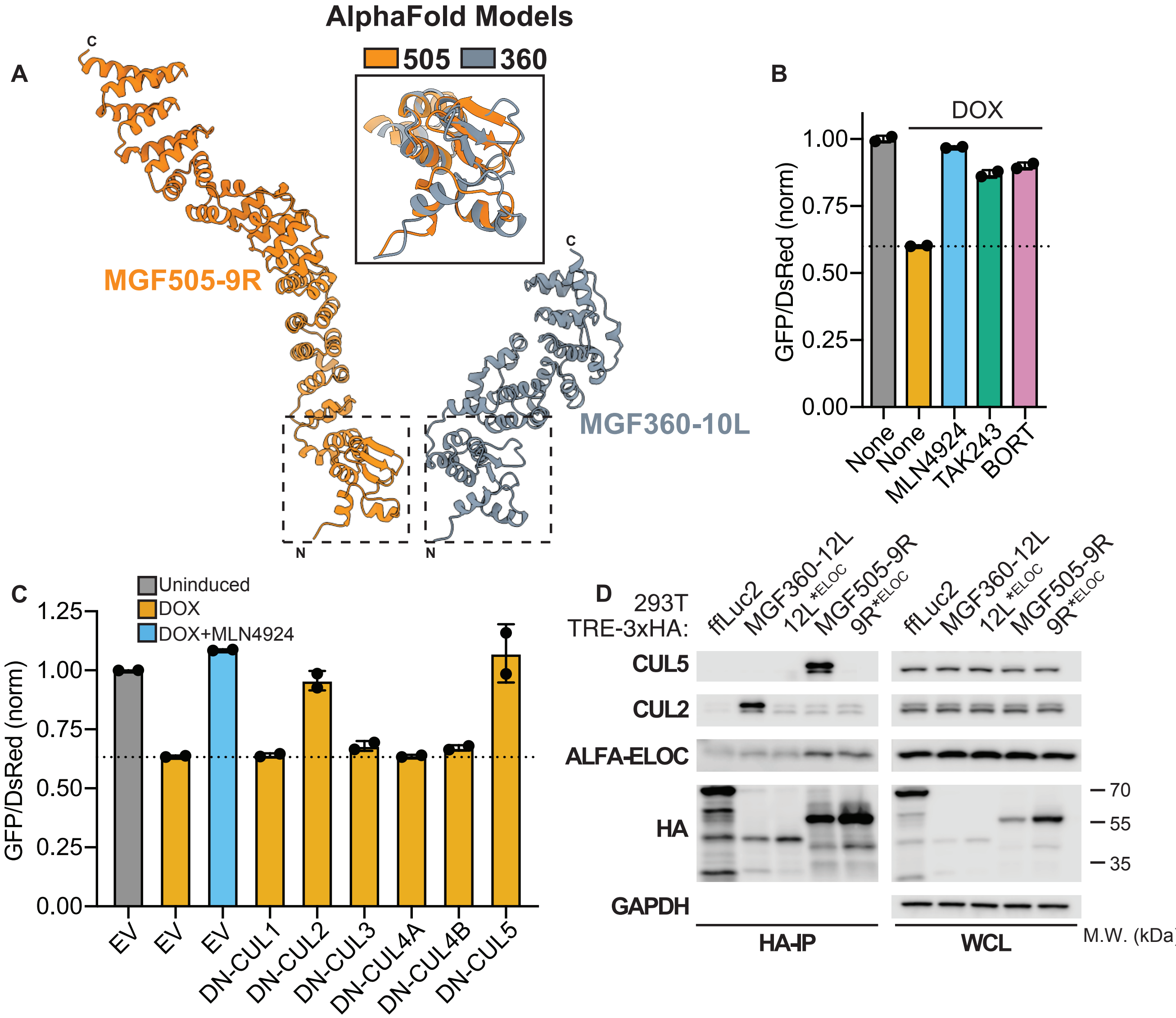

A
AlphaFold Models
505
360
MGF505-9R
MGF360-10L
C
N
B
DOX
GFP/DsRed (norm)
1.00
0.75
0.50
0.25
0.00
None
None
MLN4924
TAK243
BORT
C
Uninduced
DOX
DOX+MLN4924
GFP/DsRed (norm)
1.25
1.00
0.75
0.50
0.25
0.00
EV
EV
EV
DN-CUL1
DN-CUL2
DN-CUL3
DN-CUL4A
DN-CUL4B
DN-CUL5
D
293T
TRE-3xHA:
ffLuc2
MGF360-12L
12L*ELOC
MGF505-9R
9R*ELOC
CUL5
CUL2
ALFA-ELOC
HA
GAPDH
70
55
35
HA-IP
WCL
M.W. (kDa)

fig. S6. Characterization of African Swine Fever Virus MGF505 and MGF360 family proteins, related to Fig. 6.

(A) AlphaFold 3 predictions of MGF505-9R (ID: P0C9U9.0.1) and MGF360-10L (ID: P0C9P3.0.1).

(B) MGF360-10L-mediated degradation requires Cullin-RING ligase activity. 293T-pINDUCER20 GPS reporter cells were treated in duplicate with 200 ng/mL doxycycline (DOX) for 12 h to induce expression of TRE-αGFPnb-MGF360-10L. In the final 8 h, 1 μM inhibitor (MLN4924, TAK243, or bortezomib) was added. Following incubation, fluorescence was measured by flow cytometry and displayed as mean ± standard deviation.

(C) MGF360-10L-mediated degradation is blocked by dominant-negative (DN) CUL2 and CUL5. 293T-pINDUCER20 GPS reporter cells with TRE-αGFPnb-MGF360-10L were transfected in duplicate with the indicated DN-CUL constructs containing a tagBFP marker. Cells were treated with 200 ng/mL DOX with or without 1 μM MLN4924 for 16 h prior to analysis of GFP/DsRed ratio in the tagBFP+ population by flow cytometry. Data show mean ± standard deviation.

(D) MGF505 and MGF360 family members display distinct Cullin binding preferences. 293T-pINDUCER20 cells with constructs encoding luciferase control (TRE-3xHA-ffLuc2), MGF360-12L (ID: YP_009927143.1), 12L∗ELOC (L5A/L8A/T9A), MGF505-9R or 9R∗ELOC (L4A/L7A/C8A) were transfected with CMV-ALFA-ELOC. 5 h after transfection, cells were treated with 200 ng/mL DOX for 16 h before HA-IP and western blotting with the indicated antibodies. Results are representative of two or more independent experiments.

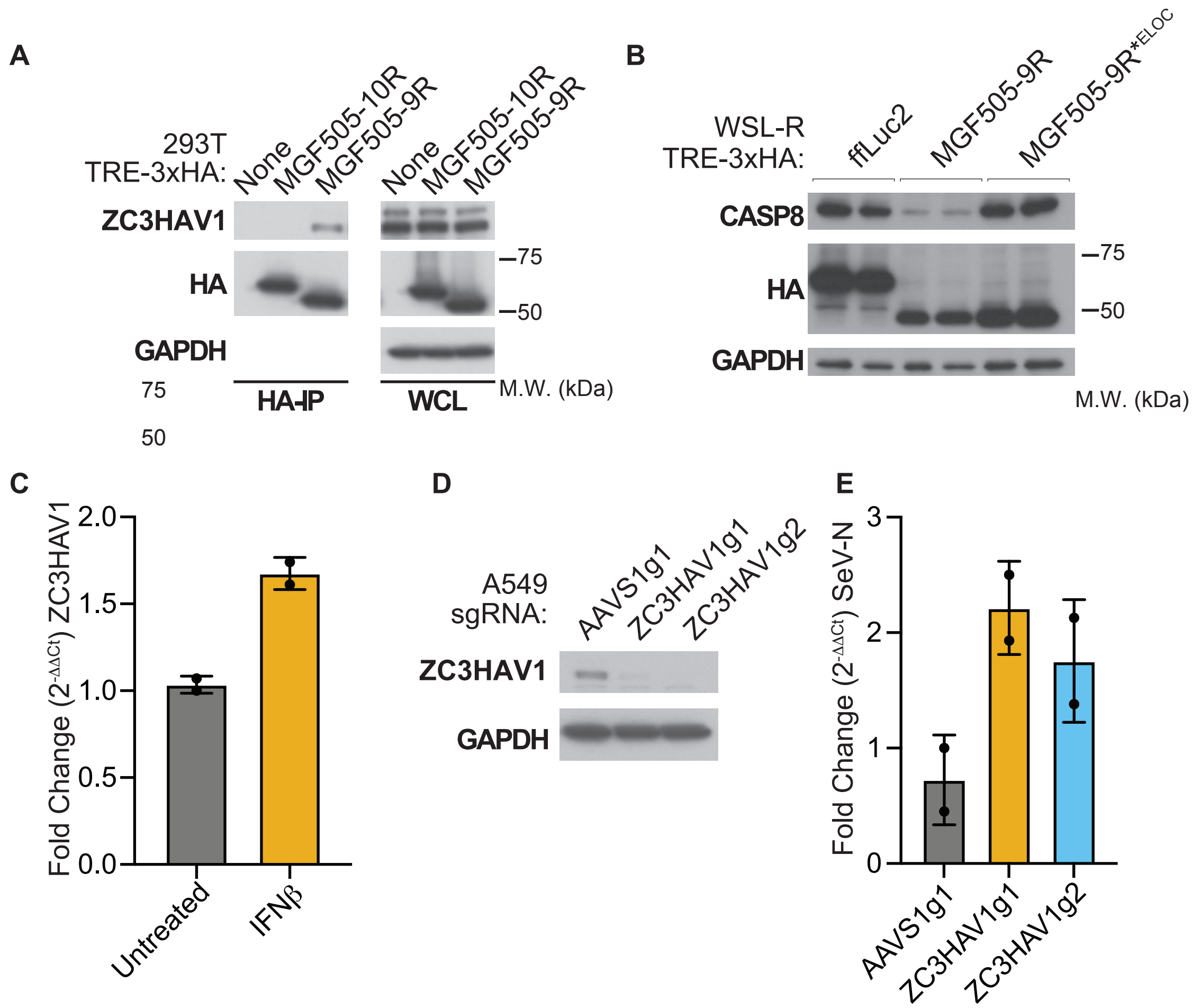
A
293T
TRE-3xHA:
None
MGF505-10R
MGF505-9R
ZC3HAV1
HA
GAPDH
75
50
HA-IP
WCL
M.W. (kDa)
B
WSL-R
TRE-3xHA:
ffLuc2
MGF505-9R
MGF505-9R*ELOC
CASP8
HA
GAPDH
75
50
M.W. (kDa)
C
Fold Change (2-ΔΔCt) ZC3HAV1
2.0
1.5
1.0
0.5
0.0
Untreated
IFNβ
D
A549
sgRNA:
AAVS1g1
ZC3HAV1g1
ZC3HAV1g2
ZC3HAV1
GAPDH
E
Fold Change (2-ΔΔCt) SeV-N
3
2
1
0
AAVS1g1
ZC3HAV1g1
ZC3HAV1g2

fig. S7. Characterization of African Swine Fever Virus MGF505-9R, related to Fig. 7.
(A) Validation of ZC3HAV1 binding. 293T-pINDUCER20 cells with TRE-3xHA-MGF505-10R (ID: P0C9V2.0.1) or MGF505-9R (ID: P0C9U9.0.1) were treated with 200 ng/mL doxycycline (DOX) and 1 μM MLN4924 for 16 h before HA immunoprecipitation (HA-IP) and western blotting. HA and GAPDH blots are shared with fig. S8A as they represent the same lysate.
(B) MGF505-9R degrades Caspase 8 in porcine cells. WSL-R-pINDUCER20 cells were transduced with TRE-3xHA-ffLuc2, MGF505-9R wild-type or mutant (∗ELOC:L4A/L7A/C8A) and treated in duplicate with 200 ng/mL DOX for 16 h prior to harvesting lysates for western blot.
(C) IFNβ treatment upregulates ZC3HAV1. A549 cells were treated in duplicate with 5 nM IFNβ for 24 h followed by 18 h incubation in complete media without IFNβ. Cells were washed with PBS prior to RNA isolation, cDNA generation, and qPCR for ZC3HAV1 and ACTB.
(D) Validation of ZC3HAV1 Cas9-mediated mutation. A549 cells were transduced with lentiviral CRISPR constructs targeting ZC3HAV1 and analyzed for expression by western blot.
(E) ZC3HAV1 Cas9-mediated mutation enhances SeV infection. A549 cells were treated with 5 nM IFNβ for 24 h prior to infection in duplicate with Sendai virus (SeV) for 18 h. Cells were washed with PBS prior to RNA isolation, cDNA generation, and qPCR for SeV-N and ACTB. Results are representative of two or more independent experiments.

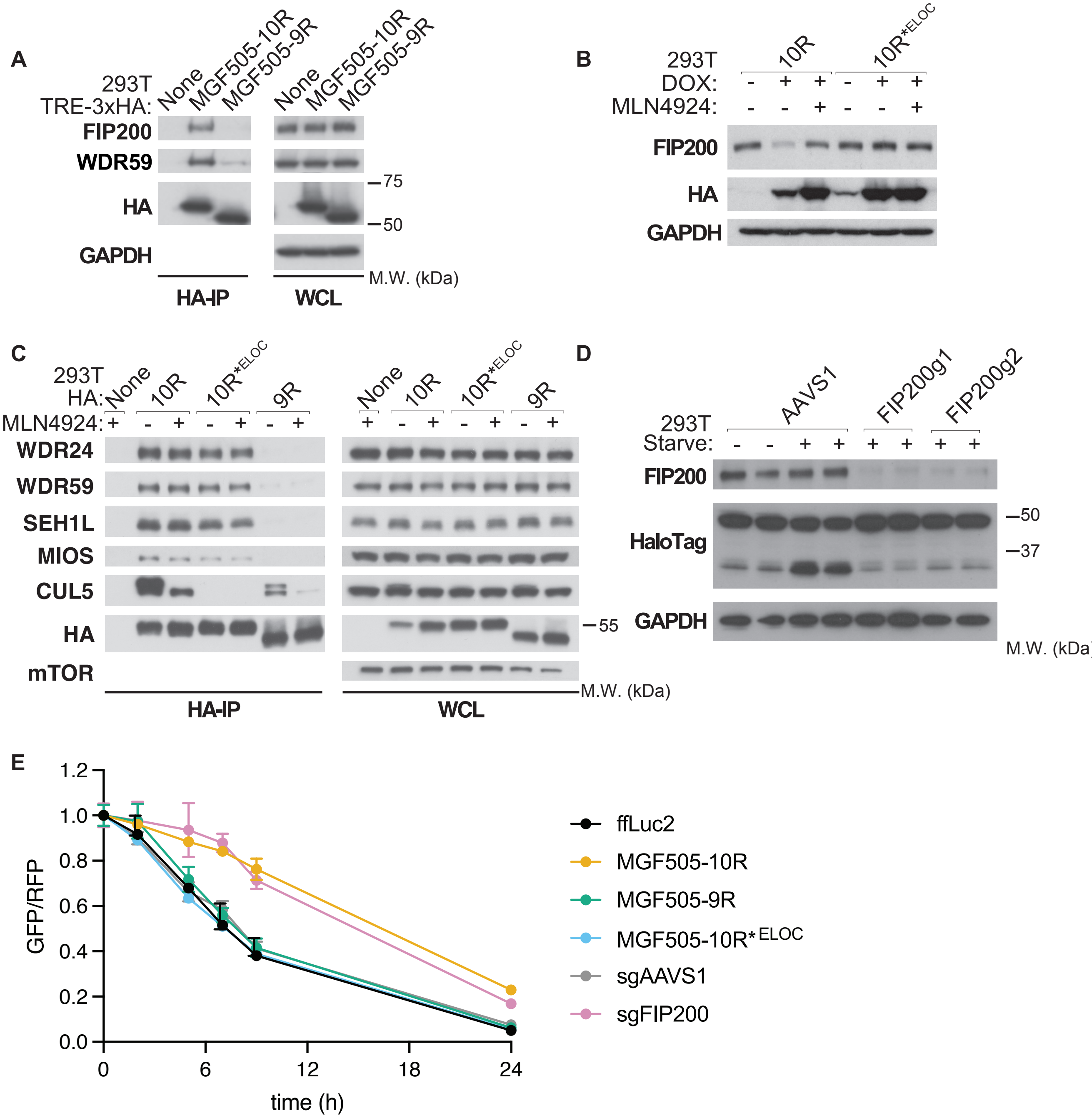

A
293T
TRE-3xHA:
None
MGF505-10R
MGF505-9R
FIP200
WDR59
HA
GAPDH
75
50
M.W. (kDa)
HA-IP
WCL
B
293T
10R
10R*ELOC
DOX:
MLN4924:
FIP200
HA
GAPDH
C
293T
HA:
None
10R
10R*ELOC
9R
MLN4924:
WDR24
WDR59
SEH1L
MIOS
CUL5
HA
mTOR
55
M.W. (kDa)
HA-IP
WCL
D
293T
Starve:
AAVS1
FIP200g1
FIP200g2
FIP200
HaloTag
GAPDH
50
37
M.W. (kDa)
E
GFP/RFP
time (h)
ffLuc2
MGF505-10R
MGF505-9R
MGF505-10R*ELOC
sgAAVS1
sgFIP200

fig. S8. Characterization of African Swine Fever Virus MGF505-10R, related to Fig. 7.

(A) Validation of FIP200 and WDR59 binding. 293T-pINDUCER20 cells with TRE-3xHA-MGF505-10R (ID: P0C9V2.0.1) or MGF505-9R (ID: P0C9U9.0.1) were treated with 200 ng/mL doxycycline (DOX) and 1 μM MLN4924 for 16 h before HA immunoprecipitation (HA-IP) and western blotting. HA and GAPDH blots are shared with fig. S7A as they represent the same lysate.

(B) MGF505-10R degradation of FIP200 is Cullin-RING ligase-dependent. 293T-pINDUCER20 cells were transduced with TRE-3xHA-MGF505-10R wild-type or mutant (∗ELOC:L4A/L7A/C8A) and treated with 200 ng/mL DOX with or without 1 μM MLN4924 for 16 h prior to harvesting lysates for western blot.

(C) MGF505-10R binds but does not degrade GATOR2 complex components. 293T cells were transfected with CMV-HA-MGF505-10R or MGF505-9R. Over the final 16 h, media was exchanged for 1 μM MLN4924 as indicated. Clarified lysates were subjected to HA-IP and western blotting for the indicated antibodies.

(D) FIP200 is required for starvation-induced LC3 degradation. 293T cells harboring HaloTag-LC3 reporter were transduced with Cas9 and guide RNA targeting AAVS1 control or FIP200. Cells were treated in duplicate with 100 nM tetramethylrhodamine (TMR)-HaloLigand for 20 min, washed twice, and cultured in amino acid-free DMEM for 6 h prior to harvesting lysates for western blot. Upper band corresponds to HaloTag-LC3 fusion, lower band represents free HaloTag generated by autophagy.

(E) MGF505-10R attenuates autophagy flux and phenocopies FIP200 loss. 293T-pINDUCER20 cells harboring GFP-LC3-RFP reporter were transduced with TRE-3xHA-vORFs or lentiCRISPR constructs targeting AAVS1 control or FIP200. Following puromycin selection, ORF expression was induced by treatment with 200 ng/mL DOX followed by starvation in amino acid-free DMEM for the indicated time, and GFP/RFP intensity was measured in duplicate by flow cytometry.

Table S1. Data collection and refinement statistics for cryo-EM datasets

NSP1 B3SRV2.0.1 ELOB ELOC CUL3 RBX1

Microscope	Titan Krios
Voltage (kV)	300
Camera	Falcon 4i
Magnification (X)	165,000
Pixel size (Å)	0.737
Total electron exposure ($e^{-}/Å^2$)	50.6
Number of frames	80
Defocus range (µm)	−2.0 to −0.8
Data collection software	EPU
Micrographs collected	12,479
Total extracted particles	5,992,159
EMDB accession code	EMD-72190
PDB accession code	PDB-9Q3E
Final particles used	173,093
Map resolution (Å, FSC 0.143)	3.3
FSC threshold	0.143
Model composition	
Protein residues	785
Ligands	3
Refinement package	phenix.real_space_refine
Model-to-map CC	0.76
Model-to-map FSC (Å, FSC 0.5)	3.3
Mean *B* factors ($Å^2$)	
Protein	64.55
Ligand	100.52
Bond root-mean-square deviation (RMSD)	
Lengths (Å)	0.005
Angles (°)	0.814
Validation	
MolProbity score	1.15
Clash score	2.90
Rotamer outliers (%)	0.16
CaBLAM outliers (%)	1.06
Ramachandran plot	
Favored (%)	97.66
Allowed (%)	2.34
Disallowed (%)	0.00

Data S1. αGFPnb screen data.
(A) Enrichment of viral genes in the bottom 1% based on GFP/DsRed ratio as analyzed by MAGeCK. (B) Read counts and enrichment for individual barcodes. (C) SignalP 5.0 filter file for the vORF library.
Data S2. CRISPR screen data.
CRISPR screen data for the ubiquitin CRISPR library. Gene level and guide level enrichment were calculated using MAGeCK comparing the top 5% based on GFP/DsRed ratio to input. (A-B) Gene level (A) and guide level (B) enrichment for Rotavirus A NSP1 (ID: Q3ZK61.0.1). (C-D) Gene level (C) and guide level (D) enrichment for TevPV-M (ID: YP_009176989.1.1). (E-F) Gene level (E) and guide level (F) enrichment for Adana virus NSs (ID: YP_009227129.1.1). (G-H) Gene level (G) and guide level (H) enrichment for Razdan virus NSs (ID: YP_008719919.1.1). (I-J) Gene level (I) and guide level (J) enrichment for ASFV MGF505-9R (ID: P0C9U9.0.1).
Data S3. IP-MS data.
Spectral counts and intensity data for HA-IP of the indicated vORF in 293T cells. (A) Rotavirus A NSP1 (ID: Q3ZK61.0.1). (B) TevPV-M (ID: YP_009176989.1.1). (C) Adana virus NSs (ID: YP_009227129.1.1). (D) Razdan virus NSs (ID: YP_008719919.1.1). (E) ASFV MGF505-9R (ID: P0C9U9.0.1). (F) ASFV MGF505-10R (ID: P0C9V2.0.1). (G) Filtering list containing common contaminants in 293T HA-IP and LC-MS/MS experiments.
Data S4. Whole-cell proteomics data.
(A) TevPV-M (ID: YP_009176989.1.1). (B) Razdan virus NSs (ID: YP_008719919.1.1). (C) ASFV MGF505-9R (ID: P0C9U9.0.1). (D) ASFV MGF505-10R (ID: P0C9V2.0.1).
Data S5. List of commercial antibodies used in this study.
Data S6. List of oligos and sgRNA sequences used in this study. (A) DNA oligos used for subcloning. (B) Guide RNA sequences for lentiCRISPR cloning.
Data S7. Amino acid sequences of human and viral open reading frames.
Data S8. Raw western blot data.
